# Randomized Trial of Time-Limited Interruptions of Protease Inhibitor-Based Antiretroviral Therapy (ART) vs. Continuous Therapy for HIV-1 Infection

**DOI:** 10.1371/journal.pone.0021450

**Published:** 2011-06-28

**Authors:** Cynthia Firnhaber, Livio Azzoni, Andrea S. Foulkes, Robert Gross, Xiangfan Yin, Desiree Van Amsterdam, Doreen Schulze, Deborah K. Glencross, Wendy Stevens, Gillian Hunt, Lynn Morris, Lawrence Fox, Ian Sanne, Luis J. Montaner

**Affiliations:** 1 Clinical HIV Research Unit, Faculty of Health Sciences, Department of Medicine, University of the Witwatersrand, Johannesburg, South Africa; 2 The Wistar Institute, Philadelphia, Pennsylvania, United States of America; 3 Division of Biostatistics, University of Massachusetts, Amherst, Massachusetts, United States of America; 4 Departments of Medicine (Infectious Diseases) and Biostatistics and Epidemiology, University of Pennsylvania, Philadelphia, Pennsylvania, United States of America; 5 Department of Molecular Medicine and Haematology, University of the Witwatersrand and the National Health Laboratory Services, University of the Witwatersrand, Johannesburg, South Africa; 6 National Institute for Communicable Diseases, Johannesburg, South Africa; 7 Division of AIDS, National Institute of Allergy and Infectious Diseases (NIAID), National Institutes of Health (NIH), Department of Health and Human Services (DHHS), Bethesda, Maryland, United States of America; University of Cape Town, South Africa

## Abstract

**Background:**

The clinical outcomes of short interruptions of PI-based ART regimens remains undefined.

**Methods:**

A 2-arm non-inferiority trial was conducted on 53 HIV-1 infected South African participants with viral load <50 copies/ml and CD4 T cell count >450 cells/µl on stavudine (or zidovudine), lamivudine and lopinavir/ritonavir. Subjects were randomized to a) sequential 2, 4 and 8-week ART interruptions or b) continuous ART (cART). Primary analysis was based on the proportion of CD4 count >350 cells(c)/ml over 72 weeks. Adherence, HIV-1 drug resistance, and CD4 count rise over time were analyzed as secondary endpoints.

**Results:**

The proportions of CD4 counts >350 cells/µl were 82.12% for the intermittent arm and 93.73 for the cART arm; the difference of 11.95% was above the defined 10% threshold for non-inferiority (upper limit of 97.5% CI, 24.1%; 2-sided CI: −0.16, 23.1). No clinically significant differences in opportunistic infections, adverse events, adherence or viral resistance were noted; after randomization, long-term CD4 rise was observed only in the cART arm.

**Conclusion:**

We are unable to conclude that short PI-based ART interruptions are non-inferior to cART in retention of immune reconstitution; however, short interruptions did not lead to a greater rate of resistance mutations or adverse events than cART suggesting that this regimen may be more forgiving than NNRTIs if interruptions in therapy occur.

**Trial Registration:**

ClinicalTrials.gov NCT00100646

## Introduction

Antiretroviral Therapy (ART) is available in resource-constrained countries through governmental and international funding programs, which have begun to impact the survival of communities [1]. Several studies suggest that adherence to ART in sub-Saharan Africa is adequate [2], but may be disrupted by supply chain interruptions, stock outs, power outages affecting medication storage, employment migration, conflict, and significant cultural stigma [3], [4], [5]. For example, rates of therapy interruptions in regular therapy management in Sub-Saharan Africa have been reported at 12.8 per 100 person years [6].

Prior studies of CD4-guided ART interruptions in patients with low CD4 counts have not been promising: the largest study to date (SMART) showed that a strategy of structured treatment interruptions (STI) guided by CD4^+^ T cell count (average time off therapy of 16.8 months) was associated with significant non-HIV related morbidities [7], [8]. The duration of uncontrolled HIV viral replication was associated with higher risk of opportunistic infections (OI) [9]. A second study evaluating structured interruptions in Corte d'Ivoire (TRIVACAN study [10]) assessed three scheduled interruptions of 2 months each followed by 4 months of continuous ART (c-ART) compared with continuous NNRTI-based ART in subjects with a median pre-ART CD4^+^ T cell count nadir of 272 cells/µl. While the interruption arm was considered to be non-inferior, there was a significantly higher rate of resistance to non-nucleotides (NNRTIs) in the experimental arm. Importantly, both SMART and TRIVACAN had low re-treatment thresholds (CD4^+^ T cell count <250 cells/µl), exposing subjects to prolonged periods of immunodeficiency. Results from the CD4-driven STACCATO study [11], with re-treatment threshold of CD4^+^ T cell count <350 cells/µl, suggest that higher CD4^+^ thresholds may minimize the risks of interrupting ART, although even in that study the interrupting ARM had higher frequency of candidiasis.

While open-ended CD4-driven treatment interruptions result in worse outcomes for subjects interrupting ART, trials incorporating fixed-length shorter treatment interruptions have yielded mixed results: the DART study used pre-determined cyclic 12 week ART interruption arms and found this strategy to be inferior due to a higher rate of disease progression than in continuous therapy. However, this study included patients with low CD4^+^ T cell count nadirs (86 cells/µl) and 52% of DART participants had a previous episode of oral or oesophageal candidiasis [12]. The ISS-PART study, conducted in Italy with ART interruptions increasing in length from 1 to 3 months, did not result in virological failures even though resistance mutations and lower frequency of CD4^+^ T cell count >500 cells/µl were noted in interrupting subjects [13]. The France-based ANSR-106 study [14], which used a 2-month on/2-month off treatment strategy, did not result in clinical endpoint differences between arms (CD4^+^ T cell count >300 cells/µl), but the interrupting arm had higher frequency of lymphadenitis and candidiasis. The ACTG-5068 study [15] incorporated 2 ART interruptions of 4 and 8 weeks. In this study, subjects on the interrupting arm were able to maintain a viral load <1000 copies/ml at greater frequency that subjects on the continuous ART arm, indicating a possible advantage for intermittent treatment strategies. Time to rebound to VL>5000 copies/ml upon ART cessation were similar between interrupting and continuous treatment arm in our Philadelphia-based study [16], which also found no significantly different clinical outcomes.

Together, the current literature (reviewed in [17], [18]) suggest that long (e.g. mean 16.8 months in SMART), open ended ART interruption carry an immunological penalty for individuals on NNRTI-based regimens, even when relatively high CD4^+^ T cell count safety thresholds are in place. However, the immunological and clinical consequences of brief (up to 8 weeks) ART interruptions in individuals with higher CD4^+^ T cell count on a PI-based regimen has not been determined, and the present study was initiated with the intent of assessing the immunologic and virologic changes occurring as a consequence of such interruptions.

## Methods

The protocol for this trial and supporting CONSORT checklist are available as supporting information; see Checklist S1 and Protocol S1.

### Ethics Statement

The study was conducted at the Themba Lethu HIV care and treatment clinic located in a secondary government hospital in Johannesburg South Africa.

Written informed consent was obtained from all study subjects; consent procedure included two educational sessions with confirmed understanding by the site investigator. Study protocol and consent form/procedures were approved by the University of Witwatersrand Ethics Committee and the IRB at Wistar Institute.

### Objectives

To test the hypothesis that intermittent short cyclic treatment interruptions would be non-inferior to continuous therapy in maintaining ART-mediated immune reconstitution, we conducted an open labelled randomized controlled study of short ART interruption in patients with CD4^+^ T cell count >450 on therapy as compared with continuous treatment.

### Study participants

Eligible participants were ≥18 years of age, ART naïve with CD4^+^ T cell count between 200–350 cells/µl, no history of opportunistic infection, and laboratory values≤grade 2 (per the DAIDS toxicity table August 2004 [19]). Patients had to be off all immunomodulatory therapy 4 weeks before enrolling and thorough out the study. Pregnant and breast feeding women and those with hepatitis B or C infection were not eligible.

### Study procedures

Patients were started on weight based stavudine, lamuvidine and lopinavir/ritonovir regimens and were allowed to switch to zidovudine for stavudine toxicity. Other ART switches were not allowed and patients were excluded prior to randomization if another ART regimen was needed.

Participants were randomized to either the short term structured interruption arm (Arm 1) or continuous therapy arm (Arm 2) at study week 24 if the HIV viral load was confirmed to be <50 copies/ml and the CD4^+^ T cell count was ≥450 cells/µl. Other criteria necessary for randomization were laboratory values≤grade 2 [19], the absence of an active AIDS –defining diagnosis, opportunistic infection or a condition requiring acute therapy.

Subjects were randomly allocated 1∶1 to either the intermittent or continued therapy using permuted block randomization with random block sizes of 6 to 9, to maintain an equal number of subjects in each group, according to a list generated by the study statistician. A sufficient number of individual envelopes, sealed and sequentially numbered, containing the arm assignment for each subject, were provided by the study statistician, and opened in the same order by an independent individual with no patient contact (site Data Manager) at the time of randomization. Specific site training was provided to ensure the correctness of the procedure.

Participants who were randomized to arm 1 were followed every 4 to 12 weeks for 72 weeks. Three sequential interruption of treatment of 2, 4 and 8 weeks took place with 16 weeks of therapy in between each interruption, a strategy that had proven safe for participants in our prior study [16]. Interruption of ART did not occur if CD4^+^ T cell count was <400 cells/µl, viral load >5,000 copies /ml or if failure to demonstrate a viral load decline of >1 log copies/ml at 12 weeks after a previous interruption. If any of these occurred, the patient continued ART and HIV viral load and CD4^+^ T cell count were rechecked in one month. If the above criteria were not met at 16 weeks after ART, the patient was declared to have reached the primary endpoint and discontinued from the experimental procedure yet continually monitored for suppression. Patients in arm 2 were followed every 8 weeks for 72 weeks after randomization. For both arms, HIV viral load, complete blood count, CD4^+^ T cell count, liver function tests, electrolytes and blood samples were collected at each study visit. Adherence was assessed via pill count and patient self-report. If the HIV viral load was >5,000 copies/ml on ART, treatment adherence counselling was implemented and genotypic resistance testing was performed. If the HIV viral load remained >5,000 copies/ml for an additional 16 weeks the subject was declared to have reached primary endpoint.

#### Genotypic Resistance Testing

Viral RNA was extracted from plasma using the QIAamp Viral RNA Mini kit (QIAGEN Group, Germany). RNA extractions were incubated at 37°C for 2 hours with 1–5 U Heparinase (Sigma Aldrich) and 40 U RNase Inhibitor in a 10 mM Tris pH 7.5, 1 mM CaCl^2^ solution, to a total volume of 140 µl, and then re-extracted to remove the enzymes. Sequencing of the *pol* gene was done using an in-house assay [20] and BigDye Terminators v3.1 on an ABI3100 Genetic Analyzer (Applied Biosystems, Foster City, CA). Consensus sequences were aligned and manually edited using the Sequencher v4.5 software (GeneCodes, Ann Arbor, MI). Genotypic resistance was defined using the Stanford Genotypic Resistance Interpretation Algorithm (http://hivdb.stanford.edu/) and the December 2009 International AIDS Society drug resistance mutation list.

### Primary and Secondary Outcomes

The primary outcome was the proportion of CD4^+^ T cell count measurements >350 cells/µl (“success”) over 72 weeks of follow-up following randomization (i.e. 10 measurements for cART group, 13 measuremnts for intermittent ART group). The time points for measuring CD4^+^ T cell count occurred during and after 3 cyclic interruptions of 2, 4 and 8 weeks in arm 1 and every two months in arm 2. For individuals deemed to have reached the primary endpoint (e.g., opportunistic infection), CD4^+^ T cell count results at all subsequent time points were imputed to be <350 cells/µl. Secondary outcomes of the study included treatment-associated toxicity, adherence and CD4^+^ T cell count change over time.

Monitoring and Interim Analyses: interim safety monitoring reports on CD4^+^ T cell count, HIV viral loads and clinical HIV disease progression and other adverse events were reviewed by the NIH/NIAID DAIDS Therapeutic Data and Safety Monitoring Boards.

### Statistical analysis

#### Primary analysis

A one sided 97.5% confidence intervals for the difference (continuous-experimental) in mean proportion of CD4^+^ T cell count >350 cells/µl over 72 weeks post randomization between the two study arms was constructed. Non-inferiority was concluded if the right-hand limit of the confidence interval was <10%. A sample size of 52 individuals was targeted to achieve 85% power for detecting non-inferiority based on a two-group, one-sided level 0.025 t-test with an expected difference of 4 between the two arms. For individuals reaching the primary endpoint (e.g., opportunistic infection) prior to 72 weeks after randomization, CD4^+^ T cell count at all subsequent time points were treated as <350 cells//µl (“imputed”), as described in Table 1.

**Table 1:** Outcome attribution rules.

Event	Outcome	Arm 1 (n)	Arm 2 (n)
SAE	Impute failure from date of occurrence	2	2
AIDS-defining illness	Impute failure from date of occurrence	1	0
Lack of resuppression*	Impute failure from date of occurrence	3	0
Pregnancy	Censor from occurrence	0	1
Withdrawal of IC	Censor from occurrence	0	0
LTFU	Censor from occurrence	0	1
CD4 failure	Fail visit	22	5
Missing data	Censor visit	8	4

*After 12 weeks on previous ART regimen. All patients achieved VL<50 copies/ml without ART regimen changes as described in results section.

#### Secondary analyses

Adherence over the post-randomization period was defined as 1 minus the difference between the actual and expected pill counts returned, adjusted for dose (number of pills per day) and number of days since drug dispensation. Two-sided, level = 0.05 Wilcoxon rank sum tests were applied to test the null hypothesis of no difference in adherence between the two study arms.

The long-term effect of intermittent ART on the retention of the CD4 recovery benefits derived from 6 months of continuous viral suppression was also considered. Here a mixed effects model was fitted to all on-ART CD4^+^ T cell count measurements with person specific random effects, as well as fixed effects for baseline (pre-randomization) CD4^+^ T cell count, study week, randomization arm and an interaction between study week and randomization arm. Finally, we fitted a series of linear models to determine whether CD4^+^ T cell count or viral load pre-ART were predictive of CD4^+^ T cell count or viral load, respectively, at end of each interruption. For modelling approaches, Wald tests were used to test whether the fixed effects coefficients were equal to 0 and resultant p-values of less than 0.05 were considered statistically significant.

## Results

### Participants' clinical and demographic characteristics

**Figure 1:**
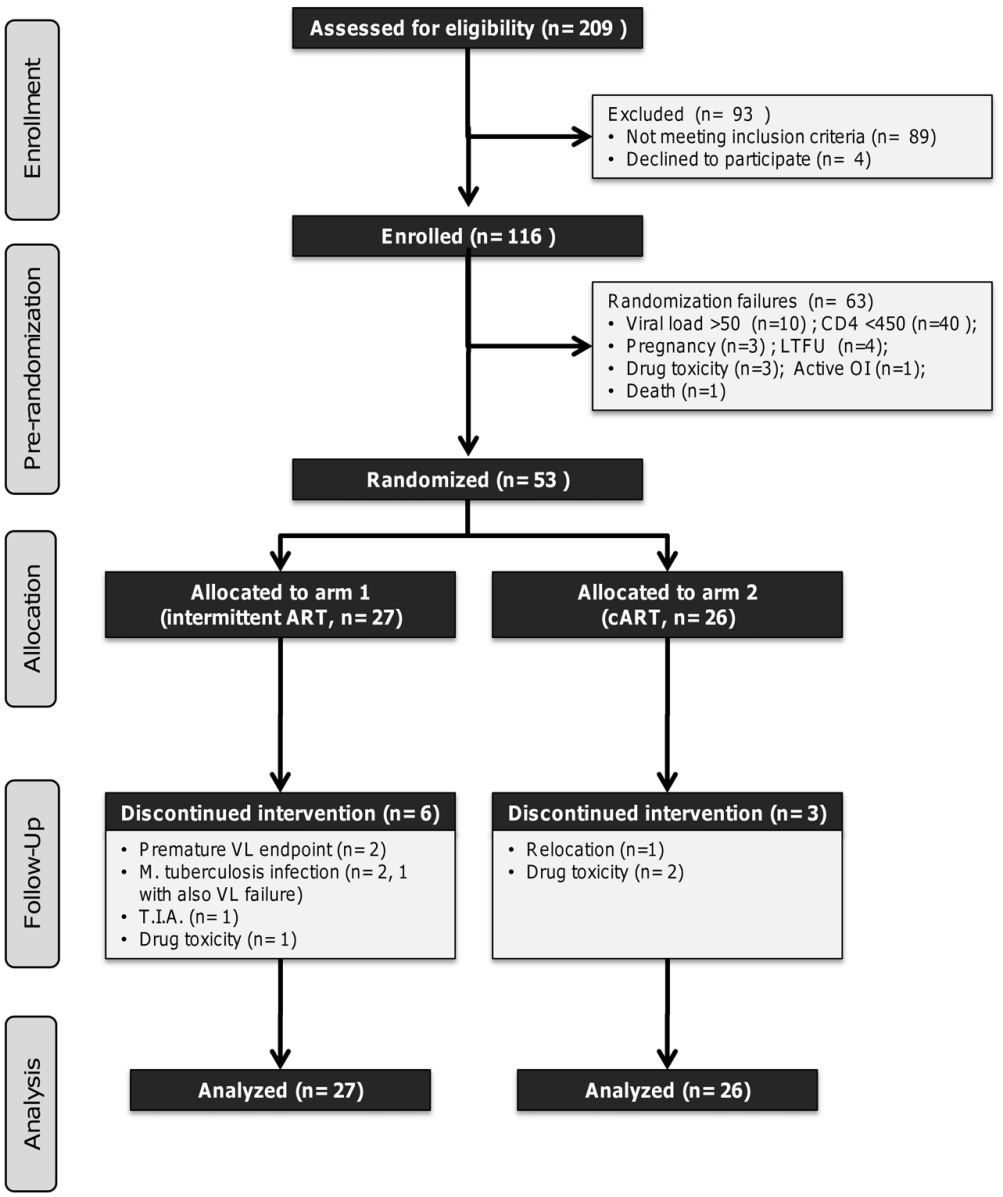
Subject disposition chart. Information regarding subject disposition during the trial is presented according to CONSORT guidelines (http://www.consort-statement.org). Abbreviations used in this table: LTFU: lost to follow-up; O.I.: opportunistic infection; T.I.A.: transient ischemic attacks.

Between July 2005 and July 2009, 116 participants were initially enrolled into the study and initiated ART (stavudine, lamivudine and lopinavir/ritonavir). There was one death pre-randomization due to sepsis and lactic acidosis. 53 subjects were randomized to intermittent treatment (arm 1, n = 27) or continuous treatment (arm 2, n = 26). Subject disposition is summarized in Fig. 1.

The study consisted of 69% women with a mean age of 35 years (SD = 8 years). The mean baseline CD4^+^ T cell count was 270 cells/µl (SD = 60) and log_10_ HIV log_10_ viral load was 4.6 (SD = 0.64) upon enrolment; at randomization after an average of 24 weeks on c-ART CD4^+^ T cell count was 512 cells/µl (SD = 115). The baseline characteristics for the participants randomized to the experimental and c-ART arms were similar (CD4^+^ T cell count: 259 and 282 cells/µl , respectively; p = 0.15. log_10_ Viral load: 4.65 and 4.69, respectively; p>0.5); one subject (arm 2) was lost to follow-up.

### Effect of ART interruptions on HIV viral load

**Figure 2:**
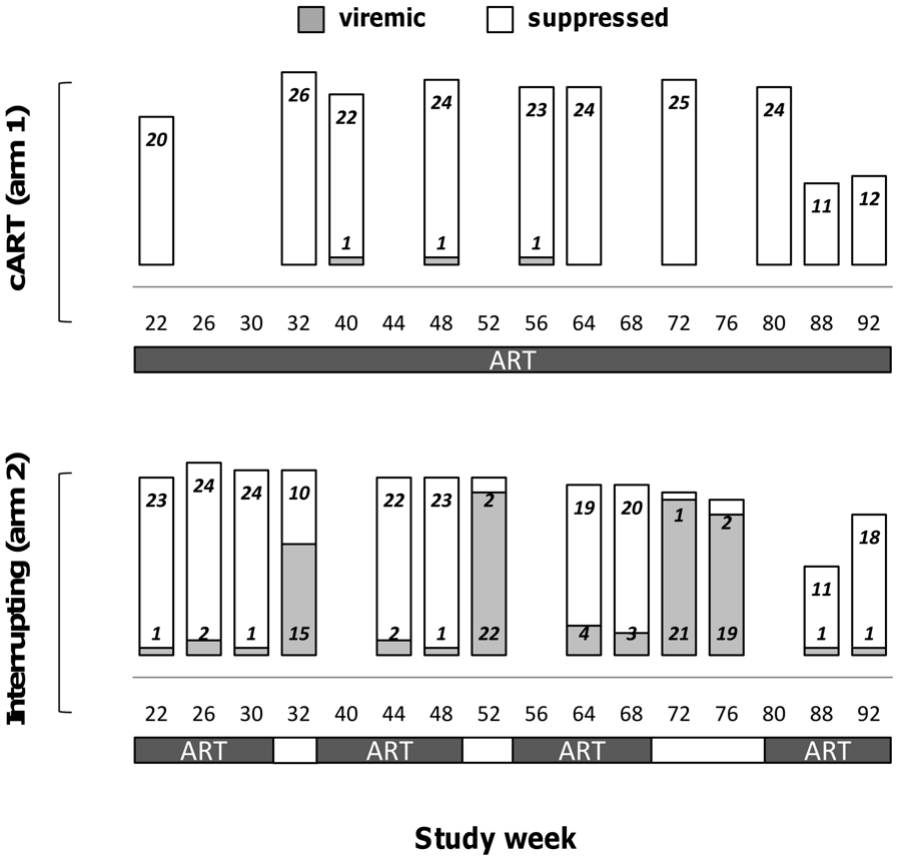
Visit viral load count outcomes. Stacked bars and numbers represent the number of subjects with HIV RNA<50 copies/ml (suppressed, closed bars) and ≥50 copies/ml (viremic, open bars) at each post-randomization visit are reported for the control continuous treatment arm (top panels, cART) and the ART-interrupting arm (bottom panel, interrupting). The treatment line below the graphics represent the time on and off ART for each study.

HIV viral load was monitored at each visit (Fig. 2); as expected, participants in the c- ART predominantly maintained undetectable (<400 copies/ml) HIV viral load throughout the post-randomization period. Treatment interruptions resulted in viral load elevation: 60% (10 of 25) of Arm 1 participants had detectable viral load during the 2-week ART interruption, 91.3% (22 of 24) during the 4-week interruption and 95% (21 of 22) during the 8-week interruption. At week 72 of post-randomization follow up, all subjects in arm 2 and 24 of 27 subjects in arm 1 had undetectable viral load. All study participants had undetectable HIV viral load at the end of the post-trial follow-up.

### Primary study outcomes

**Figure 3:**
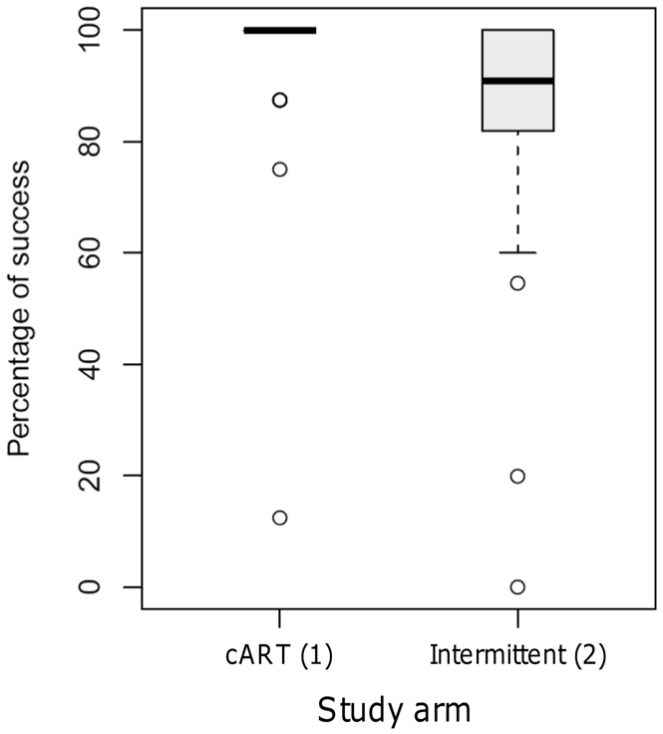
Primary study outcome. The box plot represents the percent of successful outcomes (CD4^+^ T cell count >350 cells/µl)over all post-randomization visits in the two study arms; boxes represent 5^th^, 25^th^, 50^th^ (median), 75^th^ and 95^th^ percentiles. Outliers are represented as individual circles.

Our primary outcome was frequency of CD4^+^ T cell count measurements >350 cells/µl (CD4 success) over multiple measurements in the 72-week period following randomization. As illustrated in Fig. 3, the majority of observed CD4^+^ T cell count measurements were above 350 cells/µl throughout the randomized period of the study (week 24–96) with an overall 6% of measurements (27/453) dropping below 350 cells/µl; the proportion of <350 cells/µl CD4^+^ T cell count measurements for was 22/261 (8.4%) for arm 1 and 5/192 (2.6%) for arm 2.

When post-failure visits were imputed as failed for primary endpoint analysis as listed in Table 1, the mean proportion of CD4^+^ T cell counts above 350 cells/µl was 82.12% (SD = 24.47) for arm 1 and 93.75 (SD = 17.77) for arm 2. The difference between arms in this primary endpoint was 11.6% (upper limit of 97.5% CI, 24.1%) which exceeded the threshold for non-inferiority. A two-sided 95% CI for the difference in means between arms is (−0.16, 23.4). The same conclusion is reached based on observed (non-imputed) data (mean values 91.85% for arm 1 and 97.6% for arm 2; confidence interval limit >10%; two-sided 95% CI: 1.47, 10.04).

Few clinical events occurred with only one opportunistic infection in arm 1 (pulmonary TB) and none in arm 2. Two participants developed serious illnesses (pneumonia and transient ischemic attack symptoms) after randomization thereby contributing to imputed outcomes although occurring before the initiation of any scheduled interruptions. Three other patients in arm 1 were withdrawn due to virological failure at week 16. However, there was no evidence of HIV-1 genotypic drug resistance and upon further adherence counselling off study, HIV viral loads became undetectable in all “failed” subjects. There were 2 clinical diagnoses of lipodystrophy in each arm. Post-randomization, one participant was lost to follow up in arm 2 with no losses to follow-up in arm 1. Two participants in arm 2 were terminated from the study due to withdrawals of consents. No deaths were recorded after randomization.

### Secondary study outcomes

#### Effects of intermittent ART on CD4^+^ T cell count levels

**Figure 4:**
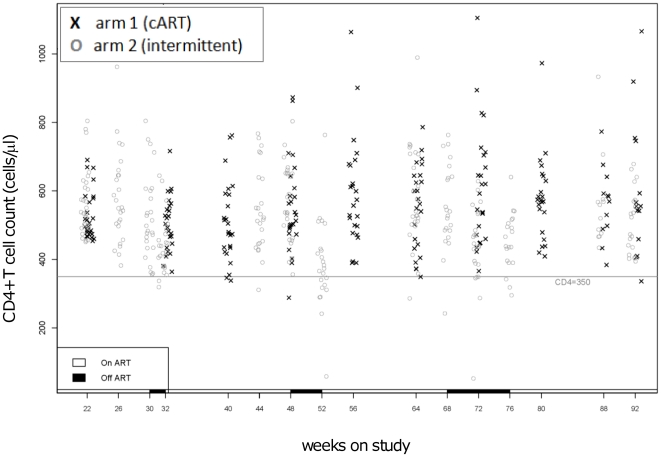
CD4 counts over the study period. The graphic represents individual CD4^+^ T cell counts at each study visit. Crosses = cART; circles = intermittent ART; the continuous line represent the CD4^+^ T cell count cutoff for failure (350 cells/µl).

**Figure 5:**
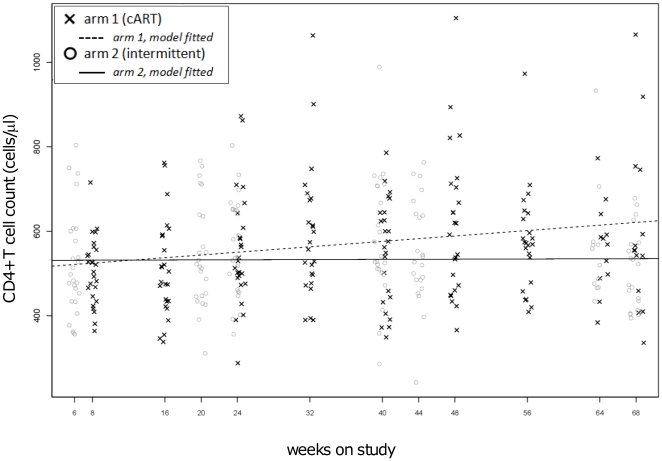
Effect of intermittent ART on CD4 count over time: mixed effect modeling. The effect of time and ARM assignment on CD4+ T cell count was assessed using a Mixed Effect linear model (MEM) as detailed in the text. the continuous lines represent the CD4^+^ T cell count predicted by the application of the model. Crosses = cART; circles = intermittent ART; dashed line: effect of time and assignment to cART arm; solid line: effect of time and assignment to intermittent ART arm.

Fig. 4 illustrates the CD4^+^ T cell count value per study subject for each study week, including those on and off ART for the experimental group. Week 22 (week 0 of randomization) represents the CD4^+^ T cell count distribution of the participants before randomization. Using a mixed-effect model, we estimated the slope of change in CD4^+^ T cell count over the entire study period (Fig. 5). The effect of time (estimate = 1.6 CD4+ T cells/µl/day; p<0.0001), was significantly affected by arm assignment (est. for interaction = −1.54; p = 0.003), indicating that arm 2 had a 1.6 cells/µl /week increase, whereas arm 1 had a 0.1 cells/µl /week change.

Baseline CD4^+^ T cell count had no detectable effect on the CD4^+^ T cell count levels measured at the end any of the interruptions (2, 4 or 8 weeks, p = 0.13; >0.5 and 0.40 respectively), as assessed in arm 1 using linear modelling. Similarly, baseline HIV viral load was also not found to predictive of off-ART viral replication (Viral Load at STI end).

#### Effects of intermittent ART on treatment adherence

**Figure 6:**
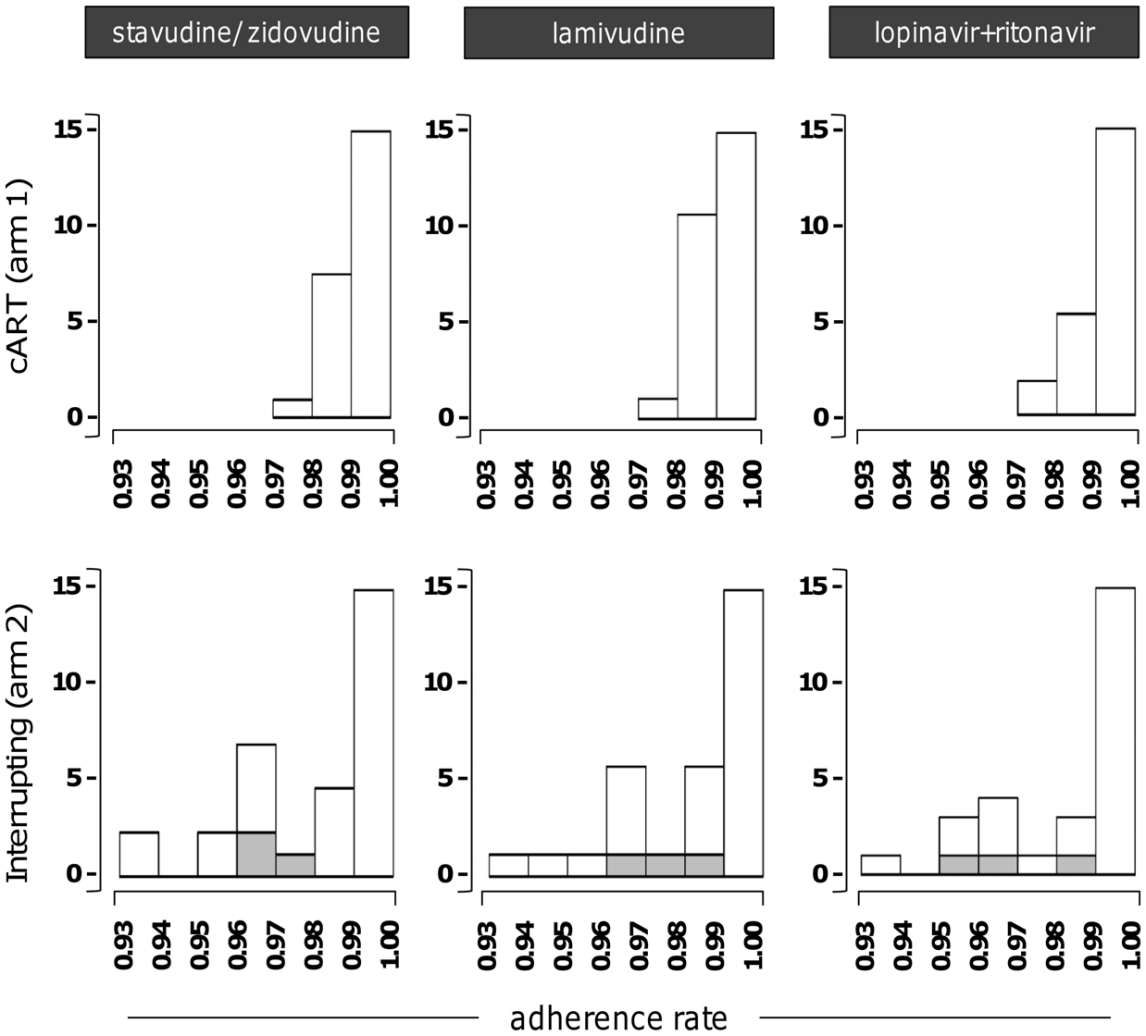
Treatment adherence. Adherence to individual ART regimen components (indicated) was calculated as specified in the text, and is represented in the form of adherence rate class distribution (from 0.93 to 1%) for the cART arm (top panels) and the intermittent ART arm (bottom panels). The contribution of the 3 individuals that failed to re-suppress to HIV RNA<50 copies/ml within 12 weeks from ART resumption is indicated as filled stacked bars.

Pill count-based adherence assessment indicated that adherence was high for all individuals for each of the three study drugs (Fig. 6). Values were 99.2% (arm 1) and 98.1% (arm 2) for stavudine, 99.2% (arm 1) and 98.1% (arm 2) for stavudine/zidovudine fixed dose combination and 99.2% (arm 1) and 98.2% (arm 2) for Ritonavir/Lopinavir. Adherence was highly correlated between drugs within individual (r>0.99, p<0.001 for all correlations). There was no evidence that the intervention had an effect on adherence while subjects were on ART.

#### Resistance testing

Samples from individuals in the intermittent arm after the last 8 week interruption were tested for evidence of genotypic resistance mutations by population sequencing. From these 24 samples, 23 reverse transcriptase (RT) and 21 protease sequences were generated. One sample had the NNRTI mutation K103N which was also present in a sample collected from the same patient 6 months previously. None of the other samples had RT resistance mutations. Another sample had the M46I mutation in the protease gene which decreases susceptibility to some PIs when present with other mutations. None of the other 20 samples had PI mutations. Only sequences from the same patient clustered together but all clustered with HIV-1 subtype C sequences.

## Discussion

We present the results of an STI study using sequential scheduled short cyclic interruption utilizing a PI-based regimen in ART naive patient with CD4^+^ T cell count nadir >200 cells/µl, CD4^+^ T cell count >450 cells/µl on therapy, and no history of OI in a resource limited setting.

According to our conservative definition of non-inferiority, which imputed treatment related adverse events as CD4 failures (see Table 1), our results do not support the hypothesis that an intermittent ART regimen is not inferior to intermittent ART. Interestingly, there was no significant excess of OI or AIDS-defining illness in arm 1 which occurred in other studies [4], [7], [8], [21]. The selection of patients with CD4^+^ T cell count nadirs >200 cells/µl and without previous history of OI or AIDs defining illnesses may have contributed to the absence of clinical events in the intermittent ART arm.

Our modelling analysis indicates that the trajectory of CD4^+^ T cell count rise over time is significantly different for subjects undergoing intermittent ART. As we did not evidence any actual loss of CD4^+^ T cells, we conclude, expanding on prior studies [16], [22], that the immunological cost of interrupting ART may be the loss of CD4^+^ T cell gains observed with continuous ART, rather than a decrease below pre-randomization levels.

Unlike previously reported NNRTI-based ART STI studies [10], [11], [21], [23], we did not observe evidence of genotypic resistance to ART in arm 1, possibly in relation to the use of ritonavir/lopinavir-based regimen. Despite the use of lamivudine, M184V resistance mutation (commonly observed in STI studies) was not found here; the K103N mutation found in one individual may be the result of exposure to ARV at an earlier time, possibly unreported single-dose nevirapine to prevent mother-to-child transmission [20], or transmitted drug-resistance. It is not clear if the M46I mutation that was found in one patient was selected by the current regimen as this mutation also occurs at low frequency in drug-naive individuals [24]; analysis of pre-ART samples would be necessary to determine if this was a naturally occurring polymorphism. The three patients that failed to re-suppress within 12 weeks after ART resumption had no detectable resistance mutations; these subjects achieved viral suppression after adherence counselling without ART regimen changes: thus, the lack of suppression appeared to be due to temporal non-adherence.

Adherence was very high in both arms (consistent with previous studies [2]) and was not significantly affected by treatment interruptions; the 24 weeks on ART before randomized may have further selected the patients with high treatment adherence, as non-adherent patients would have experienced virological failure leading to study termination before randomization [25], [26].

This study had some limitations. First, sample size was limited, making it difficult to compare our clinical outcome data against those of larger studies (e.g. SMART). Second, by incorporating safety endpoints (imputed as primary outcomes counted as loss of CD4^+^ T cells even if observed CD4^+^ T cell count >350 cells/µl), our non-inferiority definition conservatively favoured the continuous ART arm: we justify this approach under the premise that any adverse clinical outcome associated with this strategy should be reflected in primary end-point. Finally, we note that the observed sample standard deviation is much larger than the standard deviation (7.07) assumed for the initial sample size calculations. If indeed, the standard deviation were 18, then this study was powered to detect non-inferiority defined as a difference (in the mean percentage of CD4 counts >350 between groups) of less than 20%. Our conclusion that the results do not support non-inferiority remains appropriate.

While the results from the present and other studies conducted in resource-constrained countries overall do not support intermittent ART as a routine treatment strategy, treatment interruptions remain a reality of medical care in this environment, due to discontinuity in supply chains and healthcare delivery [27], displacement [28], and individual behavioural [29] and economic [30], [31], [32] adherence issues leading to treatment discontinuations and/or modifications [33]. Our data suggest that a PI-based regimen may be less likely to result in clinical failure than NNRTI-based regimens if ART interruptions occur; however, the cost of such interruptions, even in the absence of acquired genotypic resistance to the PI-based regimen or development of OI and AIDS defining illnesses, may be the forfeiture of the long-term gain in CD4^+^ T cell count that would be expected under continuous ART.

## Supporting Information

Checklist S1 CONSORT checklist.
(DOC)

Protocol S1 Study protocol.
(PDF)
