# Supplementary material for: Randomized Trial of Time-Limited Interruptions of Protease Inhibitor-Based Antiretroviral Therapy (ART) vs. Continuous Therapy for HIV-1 Infection
Source: PLoS One. 2011 Jun 28;6(6):e21450. doi: 10.1371/journal.pone.0021450 (PMC3125169; doi:10.1371/journal.pone.0021450)
Supplement: Protocol S1 — Study protocol. (PDF) [file pone.0021450.s002.pdf]

DAIDS-ES ID 10428

A Randomised Clinical Trial Assessing Continuous HAART versus Interrupted HAART in a Resource Poor Clinic (R01 A151986-01)

Sponsored by:

The National Institute of Allergy and Infectious Diseases
Pharmaceutical Support Provided by:
Department of Health, South African Government
The Wistar Institute
Non-IND Protocol

Principal Investigator: Dr. Luis J. Montaner
Clinical Principal Investigator: Dr. Cynthia S. Firnhaber MD
Protocol Statistician: Dr. Andrea Foulkes, Dept Biostatistics, U of Massachusetts
DAIDS Clinical Representative: Dr. Larry Fox
DAIDS Program Officer: Ms. Daniella Livnat

Version 5.0
June 2 2008

TABLE OF CONTENTS

I.	STUDY OBJECTIVE..	9
A.	PRIMARY AIM:	9
B.	SECONDARY AIMS	9
II.	Introduction and Preliminary data	10
A.	Epidemiology of the HIV in South Africa	10
B.	Antiretroviral therapy in South Africa	10
C.	Continuity of Treatment	11
E.	Experience with the treatment regimen Lopinavir/Ritonavir, Lamivudine, Stavudine (I. Sanne, Cynthia S. Firnhaber and M.John)	13
F.	Antiretroviral Drug Resistance in HIV-1 Clade C at AIDS Virus Research Unit, National Institute for Communicable Diseases (L. Morris)	16
G.	HIV-1 Therapy and Adherence Monitoring (R. Gross)	17
H.	Lipid Profiles, Visceral Fat and Insulin Resistance in White and Black South Africans (N.J. Crowther and M.-T. van der Merwe)	18
I.	Immune and Viral Outcomes of Therapy Interruption in Chronic Infection (L.J. Montaner)	20
J.	Outcomes on Viral Resuppression upon Retreatment and Emergence of Viral Mutations.	20
K.	Outcomes on Immune Reconstitution: CD4 T-cell Count Changes	21
L.	Outcomes on Immune Reconstitution: CD4 T-cell Recall Responses.	21
M.	Outcomes on Immune Reconstitution: T-cell subset changes.	22
N.	Outcomes on Quality of Life: Symptoms-Related Questionnaires.	22
O.	Response to Rabies Vaccine in HIV positive individuals.	22
P.	Cardiovascular risk in ART interruption	23
III.	STUDY DESIGN...	24
A.	Protocol overview	24
IV.	Selection and enrollment of subjects	26
A.	Study Site, Population and Recruitment	26
B.	Source of Study populations	26
C.	Inclusion of women and disadvantaged communities:	26
D.	Inclusion on children:	26
E.	Inclusion Criteria	27
F.	Exclusion Criteria	28
V.	REGIMENS, ADMINISTRATION, AND DURATION	29
A:	Product Formulation and Preparation	30
	Table 2 : Product Formulation and Storage Requirements.	30
	Table 3. Study product dosing	31
B.	Product Supply, Distribution, and Pharmacy	31
C.	Concomitant Medications	32
VI.	CLINICAL AND LABORATORY EVALUATION..	35
A.	Consent procedure	35
B.	Screening procedure Week – 16 visit (prior to treatment initiation) (I. Sanne, M. John, C. Firnhaber)	35
C.	Prior and concomitant medication:	35
D.	Documentation of antiretroviral therapy:	36
E.	Treatment initiation visit (enrollment) (-14 to -12) weeks	36
F.	Follow-up period weeks -8 weeks to 0 weeks:	36
G.	Follow-up period weeks 0 to +24	37

H.	Additional Inflammatory Markers	37
I.	Off treatment/ On study follow-up	37
J.	Rabies Vaccination Schedule	37
K.	Randomization	39
L.	Post-Randomization: Control Arm	38
M.	Post-Randomization: Experimental Arm	39
N.	Duration of subject participation	41
O.	Total sampling of blood volumes:	41
P.	Adherence Assessment and Feedback	41
Q.	Primary care provision	41
R.	Pregnancy	41
S.	Concomitant Medications	42
T.	Medication History	42
U.	Clinical Assessments	42
V.	Laboratory Evaluations	44
VIII.	STUDY TREATMENT TOXICITY MANAGEMENT	45
A.	Antiretroviral Therapy Dosage Reductions	45
B.	DOSAGE REDUCTION (TABLE 8)	45
C.	Toxicity Management – Grading	46
D.	Dose interruption:	47
E.	Specific Management of Laboratory Abnormalities and Clinical Syndromes	47
IX. CRITERIA FOR SUBJECT DISCONTINUATION FROM RANDOMIZATION WITH RETENTION OF FOLLOW-UP		51
IX.	CRITERIA FOR PERMANENT SUBJECT DISCONTINUATION FROM STUDY	51
X.	STATISTICAL CONSIDERATIONS	51
A.	Randomization Procedure	51
B.	Sample Size and Power Considerations	52
C.	Impact of Intermittent Therapy On Outcomes: Analysis Overview	53
D.	Primary End Point Analysis: Comparison of the Relative Maintenance of Immune Reconstitution via Maintained CD4 Recovery	53
E.	Secondary End Point Analysis: Comparison of the Relative Maintenance of Immune Reconstitution via Response to Neoantigen	55
F.	Secondary Aim Analysis: Safety/Toxicity Comparisons between groups	55
G.	Secondary Aim Analysis: Immune Reconstitution	56
H.	Secondary Aim Analysis: Resistance Mutations	57
XI.	Data collection and data management	58
A.	Clinical Data Collection: Biological Samples (I. Sanne/W. Stevens/L. Montaner)	58
B.	Clinical Data Collection: Measurement of Metabolic Variables, Adipose Tissue Distribution and Bone Mineral Density (N.J. Crowther)	60
D.	Data Collection: T cell Subsets (W. Stevens/L. Montaner)	62
E.	Data Collection: Immune Response Against Rabies (H. Ertl/ L.J. Montaner)	63
F.	Data Collection: Pre-existing T-cell Recall Responses (L.J. Montaner)	63
G.	Data Collection: Viral Genotypes (L. Morris)	64
H.	Data collection: communication and storage	66
I.	Quality Assurance/Quality Control of Data	67
J.	Clinical Site Monitoring and Record Availability	67
K.	Safety reporting	67

	Expedited Adverse Event (EAE) Reporting	67
	Procedures for Reporting of Serious Adverse Events:	68
XII.	HUMAN SUBJECTS	70
A.	Recruitment, Institutional Review Board (IRB) Review and Informed consent:	70
B.	Subject Confidentiality	71
C.	Potential risks & Precautions to minimize risks:	71
D.	Risk-benefit ratio:	72
E.	Uninfected donor standard samples:	72
F.	Study Discontinuation	73
G.	Publication of Research Findings	73
H.	BIOHAZARD CONTAINMENT	73
I.	Department of Health Support	73
XIII.	REFERENCES..	74
XIV	APPENDIX APARTICIPANT INFORMATION LEAFLET AND INFORMED CONSENT FORM..	77
XV.	PREGNANCY INFORMED CONSENT	96
XVI	APPENDIX B:	100
	..MANUAL FOR EXPEDITED REPORTING OF ADVERSE EVENTS TO DAIDS.	100

List of Investigators:

Protocol Chair:	Protocol Co-Chair
Name and Title: Luis J. Montaner, DVM, PhD<br>Director, Immunopathogenesis Laboratory<br><br>Medical Specialty: Immunopathogenesis<br><br>Institution: The Wistar Institute<br><br>Address: 3601 Spruce St., Rm. 480<br><br>City: Philadelphia State: PA, 19104 Country: USA<br><br>Telephone Number: 215-898-9143<br><br>Fax Number: 215-573-7008<br><br>E-mail/Internet Address: Montaner@wistar.org	Name and Title: Dr. Cynthia S Firnhaber<br>Visiting Professor University of Witwatersrand<br><br>Medical Specialty: Clinical HIV research<br><br>Institution: University of the Witwatersrand<br><br>Address: Helen Joseph Hospital, Perth Road, Westdene<br><br>City: Johannesburg, S. Africa<br><br>Telephone Number: +27 11 276 88 00<br><br>Fax Number: +27 11 482 2130<br><br>E-mail: cfirnhaber@witshealth.co.za
**Co-Investigator:**	**Co-Investigator:**
Name and Title: Dr. Wendy Stevens<br>Head, Molecular Diagnostic Division<br><br>Medical Specialty: Haematology<br><br>Institution: University of the Witwatersrand<br><br>Address: Medical School, York Road<br><br>City: Parktown : Johannesburg, S. Africa<br><br>Telephone Number: +27 11 717-2519<br><br>Fax Number: +27 11 484 5812<br><br>E-mail/Internet Address: wendy.stevens@nhls.ac.za	Name and Title: Nigel Crowther, Ph.D.<br>Head, Academic Research<br><br>Medical Specialty: Chemical Pathology<br><br>Institution: University of the Witwatersrand,<br>National Health Library<br>Address: 7 York Rd.<br><br>City: Parktown : Johannesburg, S. Africa<br><br>Telephone Number: +27 11 489 8525<br><br>Fax Number: +27 11 489 8451<br><br>E-mail/Internet Address: nigel.crowther@nhls.ac.za

Co-Investigator:	Co-Investigator:
Name and Title: Robert Gross, M.D., M.S.C.E.<br>Assistant Prof. of Medicine and Epidemiology<br><br>Medical Specialty: Clinical Epidemiology<br><br>Institution: The University of Pennsylvania Medical Center<br>Address: 804 Blockley Hall, 423 Guardian Dr.<br><br>City: Philadelphia State: PA, 19104<br>Country: USA<br><br>Telephone Number: 215-898-2437<br><br>Fax Number: 215-573-5315<br><br>E-mail: rgross@cceb.med.upenn.edu	Name and Title: Lynn Morris, Ph.D.<br>Professor<br><br>Medical Specialty: HIV Drug Resistance Studies<br><br>Institution: National Institute for Communicable Disease<br><br>Address: Private Bag X4, 2131<br><br>City: Sandringham: Johannesburg, S. Africa<br><br>Telephone Number: +27 11 386 6362<br><br>Fax Number: + 27 11 385 6453<br><br>e-mail: lynnm@nicd.ac.za
**Co-Investigator**	**Co-Investigator:**
Name and Title: Ian M. Sanne, M.D.<br>Clinical Director, Clinical HIV Research Unit<br><br>Medical Specialty: Clinical HIV Research<br><br>Institution: University of the Witwatersrand<br><br>Address: Helen Joseph Hospital, Perth Road, Westdene<br><br>City: Westdene: Johannesburg, S. Africa<br><br>Telephone Number: +27 11 276 8800<br><br>Fax Number: +27 11 482 2130<br><br>E-mail/Internet Address: isanne@witshealth.co.za	

Statistician:	Head of Data Management:
Name and Title: Andrea Foulkes, Ph.D.<br>Assistant Professor, Division of Biostatistics<br><br>Institution: University of Massachusetts<br>Address: 404 Arnold House, 715 North Pleasant St.<br><br>City: Amherst State: MA, 01003 Country: USA<br><br>TelephoneNumber: 413-545-1881<br><br>Fax Number: 413-545-1645<br><br>E-mail: foulkes@schoolph.umass.edu	Name and Title: Livio Azzoni, Ph.D.<br>Staff Scientist<br><br>Institution: The Wistar Institute<br><br>Address: 3601 Spruce St.<br><br>City: Philadelphia State: PA, 19104 Country: USA<br><br>Telephone Number: 215-898-1752<br><br>Fax Number: 215-573-9272<br><br>E-mail/Internet Address: azzoni@wistar.upenn.edu

Clinical Sites Participating in the Study & Study Coordinators:

Clinical Site	Local Institutional Review Board (IRB)<br>(S. Africa – clinical)
Site: Clinical HIV Research Unit (Ian Sanne)<br><br>Institution: University of the Witwatersrand<br><br>Address: Helen Joseph Hospital, Perth Road, Westdene<br><br>City: Johannesburg, S. Africa<br><br>Telephone Number: +27 11 276 8800<br><br>Fax Number: +27 11 482 2130<br><br>E-mail/Internet Address: isanne@witshealth.co.za	Chairman Name and Title: P. Cleaton-Jones<br>Chairperson, Independent Ethics Committee<br><br>Institution: Medical School, Rm 3E16, 3RD<br>University of the Witwatersrand<br><br>Address: Senate House, 1 Jan Smuts Ave.<br><br>City: Braamfontein Province: Johannesburg, S. Africa<br><br>Telephone Number: +27 11 717-2229<br><br>Fax Number: +27 11 717-2121<br><br>E-mail/Internet Address: cleatonjonesp@wits.ac.za

Study Coordinators (S. Africa):	Study Coordinator (Wistar):
Name and Title: Jennifer Segale, Desiree van Amsterdam<br><br>Medical Specialty: Nursing Sister<br><br>Institution: University of the Witwatersrand<br><br>Address: Helen Joseph Hospital, Perth Road, Westdene<br><br>City: Johannesburg, S. Africa<br><br>Telephone Number: +27 11 276 8800<br><br>Fax Number: +27 11 482 2130<br><br>E-mail/Internet Address dvanamsterdam@witshealth.co.za; jsegale@witshealth.co.za	Name and Title: Livio Azzoni, Ph.D.<br>Staff Scientist<br><br>Medical Specialty: Immunopathogenesis<br><br>Institution: The Wistar Institute<br><br>Address: 3601 Spruce St.<br><br>City: Philadelphia State: PA, 19104 Country: USA<br><br>Telephone Number: 215-898-1752<br><br>Fax Number: 215-573-9272<br><br>E-mail/Internet Address: azzoni@wistar.upenn.edu

Pharmacist	Pharmacy
Name and Title: Ms Coreen Barker<br>Rightmed Pharmacy<br><br>Institution: University of the Witwatersrand<br><br>Address: Helen Joseph Hospital, Perth Road, Westdene<br><br>City: Johannesburg, S. Africa<br><br>Telephone Number: +27 11 276 8800<br><br>Fax Number: +27 11 482 5554<br><br>E-mail/Internet Address: cbarker@witshealth.co.za	Name of Pharmacy: Rightmed Pharmacy<br><br>Institution: University of the Witwatersrand<br><br>Address: Helen Joseph Hospital, Perth Road, Westdene<br><br>City: Johannesburg, S. Africa<br><br>Telephone Number: +27 11 276 8800<br><br>Fax Number: +27 11 482 2130<br><br>E-mail/Internet Address: cbarker@witshealth.co.za

Community Advisory Board (CAB)	Data Manager (S. Africa)
Name and Title: Ms Nombuyiselo Tshandu<br><br>Address: #4 Seth Acres<br>Marula Place<br>Winchester Hills 2009<br>City: Johannesburg, South Africa<br><br>Telephone Number: 082-727-9331<br>011-882-8463<br>Fax; 27-11-882-9152<br>E-mail: nombuyiselo@workmail.co.za	Name and Title: Ms Doreen Schulze<br><br>Institution: University of the Witwatersrand<br><br>Address: Helen Joseph Hospital, Perth Road, Westdene<br><br>City: Johannesburg, S. Africa<br><br>Telephone Number: +27 11 276 8800<br>Fax Number: +27 11 482 2130<br>E-mail/Internet Address: dschulze@witshealth.co.za

I. Study Objective

Intermittent treatments cycles decrease drug exposure and may therefore reduce ART-related toxicity; however, resulting viremic periods may reduce immune competency and/or elevate metabolic risk factors as a result of inflammation. We propose to study the outcomes of short-term therapy interruptions in treatment naïve HIV-1-infected subjects achieving viral suppression on antiretroviral therapy (ART) with drugs in clinical use in sub-Saharan Africa. The treatment interruption component of the strategy proposed in our study is based on preliminary data by the proposing investigators on the safety and immune outcomes of a similar cyclic treatment strategy tested in Philadelphia under RO148398. We propose to test the hypothesis that intermittent ART is not inferior to continuous therapy in maintaining ART-mediated immune reconstitution (i.e. CD4 count recovered on ART) in HIV-infected subjects. We also hypothesize that 1) there will be no difference in the sustained cellular and humoral immune response to a de novo antigen; and 2) that repeated viremic episodes may lead to endothelial stress responses, which may lead to an elevation of overall cardiovascular risk.

A. PRIMARY AIM:

We will test our primary hypothesis by comparing endpoint CD4 counts in two groups of HIV-1-infected individuals who, after successful viral suppression (> 24 weeks at <50 copies HIV RNA/ml) on a regimen of lamivudine, stavudine and lopinavir/ritonavir, will be randomized to either remain on continuous ART (26 control subjects) or undergo cyclical ART interruptions (26 experimental subjects).

B. Secondary Aims:

- To prospectively evaluate treatment-associated toxicity (metabolic adverse events) and safety of 2-8 weeks incremental interruptions of therapy vs. continuous treatment, in newly HAART-treated patients
- To determine outcome of immune reconstitution (before and after intermittent strategy) by monitoring T-cell subsets changes and the ability to maintain cell-mediated responses against a *de novo* antigen (Rabies vaccine, administered prior to randomization) and recall antigens.
- To determine occurrence of viral evolution and genotypic changes, including minor variants, conferring resistance to lopinavir/ritonavir, lamivudine and stavudine in the HIV-1 protease and reverse transcriptase regions during intermittent and continuous treatment.
- To assess the effect of treatment interruption on cardiovascular AE risk factors.

II. INTRODUCTION AND PRELIMINARY DATA

A. Epidemiology of the HIV in South Africa

The impact of AIDS on the economic, social and political stability of resource constrained nations threatens global security. Sub-Saharan Africa is the epicenter of the AIDS epidemic, with 71% of the world's infected individuals, 83% of AIDS-related deaths and 95% of AIDS orphans [1]. The characteristics of the African epidemic (virus genotypes, transmission routes etc.) and the limitations of health care delivery reduce the applicability of knowledge acquired in clinical and laboratory studies based in the US and Western Europe on clade B virus.

According to data extrapolated from an annual sentinel site survey performed in the antenatal clinics throughout the country since 1991, an estimated >100,000 South Africans will die from HIV-AIDS in 2003, with the death rate rising to 500,000 per year by 2010 in the absence of treatment, as the prevalence of the infection is expected to rise from 13% to 27% by 2010 in the general population [2].
The changes in the antenatal clinic prevalence findings over the past 10 years have demonstrated an ongoing epidemic with high incidence of transmission, with about 4.7 million infected individuals in a country of 40 million inhabitants. The geographic distribution of HIV is varied from 8% prevalence in the Western Cape and up-to 33% in KwaZulu-Natal. The peak prevalence amongst men is consistently 25% less than that of women. By the year 2007 the average life expectancy will have declined by more than 20 years, to 30 years for women and 34 years for men [3].

The Gauteng province, inclusive of Johannesburg where this study will take place, has the highest increase in HIV prevalence in South Africa (1999=22.4% vs. 2000=29.3%) from the annual antenatal clinic sentinel surveys)[3-5].

B. Antiretroviral therapy in South Africa

The management of HIV and opportunistic infections is currently placed at a primary care level. Primary care guidelines for the prevention and treatment of opportunistic infections, and palliative care guidelines have been issued by the Department of Health, with training and implementation across the country.

The use of Highly Active Anti-Retroviral Therapy (HAART) has dramatically improved morbidity and mortality for patients with HIV/AIDS in the developed world. In resource poor countries such as South Africa, data are limited to specific research sites, two of the most experienced being the sites at which this trial will be conducted. It is inevitable, given the increasing pressure on access to treatment, that antiretroviral agents will become more widely accessible, and will need to be administered in the long-term. Using current market pricing, models of the economic viability of treatment provide employers

such as the Anglo American Corporation with a compelling argument to introduce antiretroviral therapy treatment for their employees.

A 2001 landmark lawsuit victory by the South African government versus the pharmaceutical industry has facilitated the rapid decrease in antiretroviral therapy pricing through access pricing initiatives and the introduction of generic alternative medications through voluntary licensing agreements in line with the World Trade Organization agreements and patent law.

In August 2003 the South African Cabinet announced support of a nation-wide treatment program. This has made the implementation of antiviral therapy imminent. To implement antiviral therapy on a wide-scale it is essential to develop and test regimens that are maximally effective at decreasing the burden of disease while at the same time remain simple and minimally toxic for patients.

National Department of Health antiretroviral therapy treatment guidelines have been completed and are in press. Further a tender process for the procurement of antiretroviral therapy was initiated by the Department of Health on 13 February 2004. The guidelines and tender process provide for the drugs that we are proposing to use in this study ensuring the access to the same regimen to participants under the National Roll-out program.

C. Continuity of anti-HIV-1 treatment and interruption

Table: Common causes of ART interruption in resource-constrained settings

- Unreliable drug supply
- Storage failure due to power outages
- Physician-directed ART interruption as part of AE management
- Discontinuation of health insurance coverage (seasonal and migrant workers)
- Seasonal leaves of absence with travel away from HIV clinic
- Avoidance of drugs during family visits (due to HIV stigma)
- "Pill fatigue"
- Mental illness
- Hospitalization in non-ID units

The current clinical consensus is that, once initiated according to treatment guidelines, ART should be provided as continuous treatment. However, a number of practical obstacles to continuous treatment exist, particularly in resource-constrained environments (see Table); while many situations underlying ART interruption could be addressed with infrastructural and behavioral interventions (e.g. access to "out of network" clinics, access to public clinics after termination of private healthcare coverage, better coordination of patient care, public education campaigns to battle stigma etc.), ART interruptions remain a recognized and recurring fact in the management of HIV-infected subjects. However, little is known in regards to their safety and their effect on immune reconstitution and the development of drug resistance in HIV-infected (clade C) sub-Saharan African subjects. While the current protocol does not include remote rural clinical settings, our safety assessments are expected to contribute preliminary data to conduct further research or assess the potential outcomes of interruptions of treatment in such settings.

A specific treatment interruption strategy using CD4 guided interruptions was shown to be undesirable in a large multicenter clinical trial because of increased risk of disease

progression and both AIDS and non-AIDS related events [32]. Thus, ART interruption strategies in which resumption of treatment is triggered by low CD4 thresholds should be avoided as posing excessive risk to participants.

The treatment interruption strategy proposed here is significantly different from the SMART and other CD4-guided strategies in the following points:

a- The duration of the interruptions is shorter, and ART resumption is scheduled, rather than guided by CD4 decline. In fact, in a short interruption of two weeks it is not known whether CD4 counts would decline significantly, or not. Therefore there is still uncertainty about whether or not these short interruptions would result in the same longer term outcomes as the SMART study.

b- Based on our experience with similar trials in Philadelphia, it is actually quite unlikely that CD4 decline be observed during the shortest interruptions (2 and 4 weeks). Thus, it is not expected that the outcomes of these short interruptions would be the same as the longer term outcomes of the SMART study.

c- Intense CD4 monitoring remains part of the study design, with the intent of minimizing the chances that CD4 counts be allowed to reach the threshold that has been demonstrated to increase the risk for adverse outcomes in the SMART trial (200 CD4/ul). In this revised protocol, CD4 counts will not be allowed to drop below 350 cells/ul, and viral load to increase above the lowest of a. >250,000 or b. >1.5 log above baseline viral load (see also section M)

d- In an effort to further reduce risk to participants in this study, treatment was initiated at a higher CD4 count (200 to 350) than what is recommended in national treatment guidelines for South Africa (CD4 < 200). Therefore, patients in this study are receiving treatment at earlier stages of the disease, and presumably are at less risk of AIDS-related complications than patients who initiate treatment at a (typical) CD4 count < 200.

One of the main scientific objectives of this revised study is an exploration of metabolic parameters in patients in the two study arms, to identify whether or not there is a trend towards increased expression of cardiovascular risk factors in those patients in the interrupted arm. Given the reality that many patients experience treatment interruption for various reasons, a greater understanding of metabolic changes could lead to further research to develop treatment algorithms to minimize risk in standard clinical care. Furthermore, a thorough assessment of safety issues in short term interruptions will be valuable, again, as an exploration of issues that could be addressed in larger clinical studies highly relevant to management of patients in resource-limited settings. Thus, although the strategy for CD4 guided treatment interruptions has been shown to be disadvantageous, this study will use a much more limited interruption strategy to investigate important biological and clinical parameters that can generate knowledge particularly important for resource-limited settings and be used to develop further research on improved patient care. In conclusion, although prior CD4 guided treatment interruption strategies have proved be disadvantageous, this study will use a much more limited interruption strategy to investigate important biological and clinical parameters that can be used to develop research on improved patient care.

D. Clinical HIV Research Unit (University of the Witwatersrand ACTG Site) (I. Sanne, M John and C Firnhaber)

The site at which this study will be conducted is a recognized and registered ACTU site (No. 11101 - PI Dr. Ian Sanne). As part of the ACTU the site has been registered with a clinical management plan, pharmacy plan, and laboratory plan (Contract Laboratory Services and the Department of Haematology at the NHLS under director Dr Wendy Stevens). The clinical site establishment plans for this study will use those registered under the ACTU.

The University of the Witwatersrand ACTU is a self-funded, multidisciplinary unit attached to the Medicine Department of the Medical School. Doctors in the unit have been involved in industry-funded antiretroviral trials since 1994, and the unit currently is one of the biggest HIV clinical sites in the Country having completed over 35 phase II/III trials including all the currently registered drugs in South Africa.

The laboratory site at which the T cell subsets, viral load assays and safety blood that will be conducted for this study has at least 14 years experience in the field of HIV laboratory monitoring and is affiliated to the University of the Witwatersrand and the National Health laboratory Service. This site has recently become the local central laboratory for a number of HIV related trial networks including : 1) HIV Vaccine Trial Network (HVTN), 2)International HIV/AIDS Vaccine Initiative (IAAVI) South African HIV/AIDS Vaccine Initiative (SAAVI IAAVI/SAAVI 3)AIDS Clinical Trials Group (ACTG), 4) Pediatric AIDS Clinical Trial Group (PACTG) and the 5) CIPRA project. To date the project unit supported by this unit has completed over 1000 clinical projects to date.

E. Experience with the treatment regimen Lopinavir/Ritonavir, Lamivudine, Stavudine (I. Sanne, Cynthia S. Firnhaber and M.John)

The proposed regimen for this protocol is a protease inhibitor and two nucleoside analogues reverse transcriptase inhibitors, includes antiviral agents supported by the department of health in the national roll-out plan. This study regimen has been chosen for the following reasons: (1) medication available under the UNAIDS access pricing agreement – particularly relevant to the resource poor setting, (2) a proven fully suppressive treatment combination with limited short-term toxicity (most common adverse events were abnormal stools, diarrhea and nausea [6, 7]), (3) a regimen with a low pill burden and low dosing interval, (4) preference given to a protease inhibitor containing regimen versus non-nucleoside reverse transcriptase inhibitors as the NNRTI class of medication is associated with a rapid onset of resistance, long-half life and probably inappropriate for interruption treatment strategies, (5) study medication approved by the Food and Drug Administration (FDA) and the South African Medicines Control Council (6) provide women who have received a single dose of Nevirapine as part of prevention of mother child transmission, with the risk of resistance, an opportunity to be included in the study.

The study regimen for this study is Stavudine + Lamivudine + Lopinavir/Ritonavir.

The Clinical HIV Research Unit was one of the three sites in South Africa that participated in the Kaletra® 863 protocol, "A Randomized, Double Blind, Phase III study of aBT-378/Ritonavir Plus Stavudine and Lamivudine in Antiretroviral-Naïve HIV Infected Subjects", comparing Nelfinavir bid with Kaletra bid with a background of Lamivudine and Stavudine. A total of 24 patients were followed on this protocol at our site. The drug regimen was well tolerated with only one significant drug related serious adverse event of pancreatitis. The white patients recruited demonstrated significant increases in lipid profiles, with at least one patient developing lipid related pancreatitis. The black patients, from predominantly indigent social circumstances, tolerated the regimen extremely well, with >95% treatment adherence. Due to the experience of the clinic with this regimen, the tolerance of the regimen in white and black patients and its effectiveness in suppressing viral load to <50 copies/ml within 10 weeks in >90% of therapy naïve patients, Lopinavir/Ritonavir, Lamivudine, and Stavudine is selected as the regimen to be used in this study. Furthermore, the study regimen was not associated with resistance mutations to lopinavir/ritonavir in patients who demonstrated virologic failure (0/37) and significantly lower evolution of 3TC associated M184V mutation in the LPV/r treated arm, in contrast to the Nelfinavir treated arm.

Lamivudine (3TC)

3TC is a potent nucleoside that is widely used in the management of HIV-1-infected subjects. Although 3TC is an effective antiviral reverse transcriptase inhibitor, virus with a resistance mutation at codon 184 rapidly emerges within 2 weeks of monotherapy and is also seen with dual nucleoside regimens.
3TC is one of the best-tolerated nucleoside analogues. Adverse events occur in less than 5% of subjects. Toxicities include headache, nausea and vomiting, malaise, fatigue and sleeplessness, anorexia, dizziness, rash, depression, anemia, neutropenia, and increased amylase.

Subjects who are co-infected with hepatitis B may experience increased liver function tests and exacerbation of hepatitis symptoms when 3TC is stopped. Usually these symptoms are self-limiting; however, death has been reported. The causal relationship to 3TC discontinuation is unknown. Subjects should be followed closely for the first several months following 3TC discontinuation. For more information concerning 3TC, please refer to the Lamivudine package insert. Although 3TC is an effective antiviral reverse transcriptase inhibitor, virus with a resistance mutation at codon 184 rapidly emerges within 2 weeks of monotherapy and is also seen with dual nucleoside regimens the rate of evolution of AZT resistance is decreased in the presence of M184V.

Stavudine (d4T)

Stavudine is an approved NRTI, which has been evaluated extensively as monotherapy and in combination with other NRTIs and PIs. Reductions in HIV-1 RNA and improvements in clinical status have been observed with monotherapy and combination therapy. The most common toxicity associated with d4T is peripheral neuropathy.

Decreased sensitivity has been reported by a number of mutations, including thymidine analogue mutations (TAMs) which are selected for by both Stavudine and Zidovudine. This mutation has been shown to produce some cross-resistance to other dideoxynucleoside analogs but these results are inconsistent. A mutation at codon 151, and insertions at codon 69 have been reported to produce cross-resistance to all nucleoside analogs, including stavudine**.**

There is no drug interaction with antituberculous drugs or oral contraceptives.

Lopinavir/RTV

Lopinavir/Ritonovir is available in two formulations: Kaletra BD (400/100) or Alluvia 400/100 BD: Side effects associated with lopinavir/ritonavir include diarrhea, loose stools, nausea, vomiting, headache, and rash. Elevated cholesterol, triglyceride or liver enzyme levels also are reported. Increases in blood glucose, redistribution of body fat and pancreatitis also may be associated with lopinavir/ritonavir use.

LPV is a potent inhibitor of HIV protease. When co-formulated with LPV, RTV inhibits the CYP3A-mediated metabolism of LPV, thereby providing increased plasma levels of lopinavir. LPV/RTV (LPV/r) has been evaluated for use in combination with other antiretroviral agents for the treatment of HIV-infection.

A Phase III randomized study (M98-863) evaluated the safety and efficacy of LPV/r with stavudine (d4T) and lamivudine (3TC) versus NFV with d4T and 3TC. Subjects had to have no more than 14 days of any antiretroviral therapy and no prior d4T or 3TC treatment. The primary efficacy analyses included the proportion of subjects with HIV-1 RNA < 400 copies/mL at week 24 and the duration of virologic response through week 48. Overall, 326 subjects were assigned to the LPV/r group and 327 to the NFV group. Baseline HIV-1 RNA was 4.9 $\log_{10}$ for each group. Baseline CD4+ cell counts were approximately 260 cells/μL for each group. At 48 weeks, the proportion of subjects with HIV-1 RNA levels < 400(<50) copies/mL by intent to treat (ITT) (missing value = failure) analysis were 75%(67%) for the LPV/r group compared with 63%(52%) for the NFV group ($p < 0.001$) (Proportion <400[<50] copies/mL on treatment was 93%[83%] vs. 82%[68%]). Mean change in CD4+ cell count was 207 cells/μL for the LPV/r group and 195 cells/μL for the NFV group. Overall, 2% of subjects in the LPV/r group and 4% in the NFV group discontinued therapy at or before week 48 because of study drug-related adverse events. No significant differences between the two treatment groups were noted in adverse events, except for increases in triglycerides. Moderate and severe adverse events for the LPV/r and NFV groups, respectively, were as follows: diarrhea (16% and 17%), nausea (7% and 5%), asthenia (4% and 3%), abdominal pain (4% and 3%), SGPT/ALT > 5 times ULN (4% each), total cholesterol > 300 mg/dL (9% and 5%), triglycerides >750 mg/dL (9% and 1%, $p < 0.001$), and amylase > 2 times ULN (3% and 2%).

Durability of response has been demonstrated in ART-naive subjects in Study M97-720 with viral load <400 copies/mL in 99% of subjects on treatment (ITT M = F: 80%) through 208 weeks of follow-up.

LPV/r has also demonstrated antiviral activity and tolerability in combination with NNRTIs in subjects with prior PI experience. Further details regarding LPV/r are available in the package insert.

E.1 Zidovudine as alternative treatments in protocol.

Within this protocol one treatment switch is allowed. For Stavudine drug related adverse events a switch to zidovudine is allowed. Zidovudine is generally well tolerated, particularly in subjects with CD4+ count > 200 cells/mm^3. The major side effects include headache, fatigue, malaise, nausea, anemia and neutropenia. Long-term Zidovudine therapy is associated with myopathy and rare cases of steatosis, with hepatic failure and death.

E.2 Treatment of TB during STI Trial

If a patient develops TB, the patient will be treated with a Rifibutin containing regimen instead of Rifampicin. The standard trial ARV drugs of Lopinavir/Ritonovir, Stavudine and Lamivudine will be continued. Rifabutin will be dose reduced to 150 mg 3x a week aslopinovir/ritonovir increases the drug levels of Rifabutin. The side-effects of Rifabutin are rash, gastrointestinal intolerance, headache, anaemia, thrombocytopenia and leucopenia.

F. Antiretroviral Drug Resistance in HIV-1 Clade C at AIDS Virus Research Unit, National Institute for Communicable Diseases (L. Morris)

Although we hypothesize no difference between groups in our study will be observed, our research plan will determine if different rates of failures associated with resistance are present through resistance tests to be performed at the The National Institute for Communicable Diseases (NICD). The NICD is approximately 8 miles from the Clinical Trials Unit. Prof Morris's Virology laboratories comprises ten thousand (10,000) square feet subdivided into areas for cellular and molecular biology work. This includes 2 cell culture laboratories, 1 clean laboratory including a pre-PCR area, 1 transfected cell laboratory, 2 post-PCR laboratories, 1 protein purification laboratory, 1 DNA sequencing laboratory, 1 Gel Doc Room, an equipment room and freezer storage areas. The 2 cell culture laboratories are used for sample processing and culture work involving infectious HIV. These laboratories have secure key-pad controlled access, HEPA-filtered biohazard hoods fitted with extractors, centrifuges with sealed buckets and separate incubators for uninfected and HIV-infected cultures. There are strict safety guidelines for those working with infectious materials. Biohazard waste is placed in sealed containers and autoclaved and then incinerated.

Due to the limited use of antiretroviral therapy in South Africa, little drug resistance is to be expected at the onset of therapy. Studies in South Africa among drug naïve HIV-1 infected individuals showed no evidence for mutations associated with primary drug resistance to both reverse transcriptase (RT) and protease (PR) inhibitors [8-10]. All loci in the RT gene associated with resistance to AZT, 3TC, d4T, ddI and NVP were found to be wild-type. However, similar to other studies we found that the subtype C PR consensus sequence encodes M36I and I93L [10] that function as secondary drug

resistance loci for Ritonavir and Lopinavir in subtype B infection. The subtype C PR consensus differed by 8 amino acids from the subtype B consensus. Whether these accessory mutations will influence the time to failure with the regimen to be tested here is unknown. Overall, these data indicate that untreated HIV-infected individuals in South Africa harbor viruses that would be expected to be fully sensitive to ARV drugs.

Studies performed on mother and infants participating in clinical trials of short-course ARV to prevent mother-to-child transmission have shown that the use of AZT/3TC (the PETRA study sponsored by UNAIDS) or ddI/d4T (BMS study A1455-094) was not associated with the development of resistance mutations ([11]and unpublished data). However the use of NVP was shown to result in the appearance of resistance mutations in mothers and infants 6 weeks after birth. The predominant mutations were K103N, Y181C, Y188C and G190A seen in approximately 50% of women analyzed (unpublished data). This work is ongoing and includes follow-up of 800 women and infants receiving single-dose NVP to determine the prevalence and persistence of these mutations. This work is being performed using both the ViroSeq and an in-house resistance assay.

Additional studies performed on 31 patients failing ARV therapy have also been done. Most patients had received at least 2 NRTI's (AZT, 3TC, ddI, d4T or ABC) and either one NNRTI (NVP or EFZ) or a PI (LPV, NFV or RTV). Assays were performed using the ViroSeq genotyping system. Twenty-nine patients were infected with subtype C viruses, while 2 were infected with subtype B. The most frequent mutation detected was M184I/V (13/31, 42%), followed by D67N (11/31, 35%), T215Y(9/31, 29%), K70R (8/31, 26%), M41L (6/31, 19%) and K65R (4/31, 13%) reflecting the heavy use of 3TC and AZT in the cohort. K103N (6/31, 19%), G190A (6/31, 19%) and Y181C (4/31, 13%) were found among patients failing NVP or EFV containing regimens. Of the fourteen patients that received a PI, four had evidence of resistance mutations including D30N, M46I, I54V, V82A, N88D/S and L90M. The M36I and I93L polymorphisms were present in all except one or two subtype C PR sequences and approximately half were polymorphic at position 63. The presence of subtype C mutations associated with failing continuous therapy underscores the importance of monitoring for these changes between continuous and short-term interruption groups as proposed in our research plan.

G. HIV-1 Therapy and Adherence Monitoring (R. Gross)

Adherence to therapy is a crucial factor for the interpretation of the effects of as antiretroviral therapy. In addition, when testing the efficacy of interventions such as short cycles of intermittent therapy, measuring adherence to the intervention is magnified. Several methods of measuring adherence exist, including self-reports, electronic monitoring systems, and pill counts. All are believed to be valid measures of adherence. However, they each have potential strengths and limitations. Self-reports are thought to be accurate when an individual admits non-adherence, yet much less so when they report perfect adherence and the self-report instruments have not been translated into any of the indigenous languages of South Africa (e.g., Zulu). The Medication Event Monitoring System (MEMS -APREX Corporation, Menlo Park, CA), is a commercially available electronic recorder of the time and day of each opening that resides in the cap of a pill bottle. While these devices have been shown to provide a valid measure of adherence [12, 13], they are limited by their known failure rate and the potential for loss of the cap. In

addition, they may be considered cumbersome by individuals carrying their medications with them on a daily basis, especially if used for long periods of time [14]. Their cost is a further barrier to their use in a resource constrained setting.

Pill counts provide direct evidence that a medication is not taken if the pills remain in the bottle at the time of the study visit. Although in practice this approach can be circumvented by pill dumping, this technique has been successfully been used in the South African setting [15] and the Johannesburg clinical research staff have used this measure successfully in the past. Furthermore, pill dumping can be detected by providing excess drug for the monitored period and determining if adherence rates exceed 100%. If the extra doses are missing at the time of the study visit, an individual can be queried in a more detailed manner regarding their adherence, which may reveal the pill dumping. Based on issues of feasibility, cost, and convenience to the study subjects, we have chosen to use pill counts as the measure of adherence in this study.

H. Lipid Profiles, Visceral Fat and Insulin Resistance in White and Black South Africans (N.J. Crowther and M.-T. van der Merwe)

Studies carried out by our group (NJ Crowther and M-T van der Merwe et al.) in South Africa using the euglycemic, hyperinsulinemic clamp technique, have shown that, obese black females are more insulin resistant than BMI-matched obese white females [16]. This observation was further confirmed using in vitro analysis of adipose tissue isolated from the same two populations. This investigation demonstrated that subcutaneous adipose tissue from obese black females was more insulin resistant than that from obese white females [17]. The lipid profile of black South African subjects is known to be less atherogenic (i.e. lower triglyceride, total cholesterol and LDL-cholesterol but higher HDL-cholesterol levels) than that of white subjects and this has been confirmed in our studies [18-20].

Variables	Lean Black	Lean White	Over-weight Black	Over-weight White	Obese Black	Obese White
N number	24	38	20	32	25	18
Age	31±10	40±17*	44±13	51±17	42±8	48±14*
BMI	22.5±1.6	22.6±1.4	26.8±1.3	27.2±1.5	32.2±1.5	31.9±1.6
Glucose	4.5±0.6	4.9±0.4*	4.8±0.1	5.0±0.1	4.8±0.1	5.0±0.1
Insulin	79±9	47±3*	90±20	52±5*	97±6	63±5*
Proins.	3.0±1.0	2.4±0.4	3.5±0.8	2.6±0.8	2.8±0.4	2.1±0.3
Trigs.	0.80±0.09	1.08±0.06*	0.92±0.09	1.49±0.12*	1.19±0.15	1.59±0.19
Cholest.	4.2±0.2	5.0±0.1*	4.8±0.2	5.5±0.1*	4.5±0.2	5.6±0.2*
LDL	2.5±0.2	3.0±0.1*	2.8±0.1	3.4±0.1*	2.7±0.1	3.5±0.2*
HDL	1.4±0.1	1.5±0.05	1.5±0.1	1.4±0.1	1.2±0.1	1.4±0.1
HOMA	2.3±0.2	1.5±0.1*	2.7±0.6	1.7±0.2*	3.1±0.2	2.0±0.1*
Waist:hip	0.79±0.07	0.77±0.07	0.80±0.09	0.86±0.09*	0.81±0.08	0.86±0.09*

Table 1. Anthropometric and metabolic indices in lean, overweight and obese black and white South African subjects Means ± SEM (except age, BMI and Waist-to-hip [Mean±SD]). *p<0.05 vs black of appropriate BMI group. Statistical test was ANCOVA correcting for age. Proins.=proinsulin, Trigs.=triglycerides, Cholest.=total

Visceral abdominal fat depot is known to have effects on lipid metabolism leading to raised triglyceride and LDL-cholesterol and lowered HDL-cholesterol levels [21]. Investigations by our group using computerized tomography (CT) have now shown that the visceral adipose tissue depot is smaller in obese black than white females, matched for BMI [18-20, 22]. In an unpublished study (van der Merwe et al.) these findings have been confirmed using magnetic resonance imaging (MRI) in 52 obese white females (BMI 38.5±0.8) and 18 obese black females (BMI 38.1±0.9). Visceral fat area in the white females was 103±10 cm2 compared to 74±10 cm2 in the black females (p<0.01). A positive correlation was found between the size of the visceral fat depot and the serum level of triglycerides[18, 20] and a negative association with serum HDL levels [20]. A recent study (unpublished) carried out in collaboration with Dr N Chetty and Mr NH Naran from the Department of Molecular Medicine and Haematology, National Health Laboratory Service and University of the Witwatersrand, Johannesburg has confirmed these findings. Fasting levels of insulin, proinsulin, glucose, total cholesterol, HDL-cholesterol and LDL-cholesterol (calculated using the Friedwald formula [23]) were measured in randomly selected healthy, white and black South African subjects. Waist-to-hip ratio was also measured. Lipid and glucose levels were measured in the Department of Chemical Pathology's routine service laboratories and insulin and proinsulin in the Department of Chemical Pathology's research laboratory (Dr. Crowther's unit). Insulin resistance was calculated from the fasting glucose and insulin levels using the HOMA formula [24]. Both population groups were split into 3 groups according to BMI: lean group, BMI 20-24.9; overweight group, BMI 25-29.9 and obese group, BMI 30-34.5 (BMI=weight (kg) / height (m)2). Within each of these 3 groups the white and black subjects were matched for BMI and age and data is shown in Table 1 The above data demonstrate that black subjects are more insulin-resistant and have more favorable lipid profiles than white subjects in all BMI groups. Additionally waist-to-hip ratio is higher in white than black subjects in overweight and obese groups. Waist-to-hip ratio is a good indicator of abdominal fat mass. Importantly, the data produced in Johannesburg shows that black subjects, who are not on anti-retroviral treatment, have a more favorable lipid profile, lower visceral fat mass but higher insulin resistance than white subjects.

Anti-retroviral therapy is known to produce increased abdominal fat deposition, dyslipidemia and insulin resistance [25-28] and therefore black subjects on anti-retroviral treatment may be more susceptible to the diabetogenic properties of the treatment but less susceptible to the atherogenic properties. The present study is therefore very important in that it will test this hypothesis and also show whether intermittent treatment with anti-retrovirals can reduce their toxic side effects, particularly the increase in insulin resistance that has been reported in other studies [26, 27].

I. Immune and Viral Outcomes of Therapy Interruption in Chronic Infection (L.J. Montaner)

The Wistar Institute is an independent nonprofit biomedical research institution dedicated to discovering the causes and cures for major diseases, including cancer, cardiovascular disease, autoimmune disorders, and infectious diseases. Founded in 1892 as the first institution of its kind in the nation, The Wistar Institute is also a National Cancer Institute-designated Cancer Center - one of only eight focused on basic research. Discoveries at Wistar have led to the development of vaccines for such diseases as rabies and rubella, the identification of genes associated with breast, lung, and prostate cancer, and the development of monoclonal antibodies and other significant research technologies and tools.

During the previous 40+ months, Dr. Montaner's group at the Wistar Institute have followed the immune and viral outcomes of over 57 monitored therapy interruptions (TI) in a subset of 58 HIV infected subjects (all of these with over 100 days of follow-up) representing an observational cohort that interrupts treatment under the supervision of their providers and patients enrolled in a randomized, single site trial on short cycles of treatment interruption in Philadelphia. In our current study of sequential TIs of 2,4, and two 6 weeks, which is similar to the proposed design we have not observed any study-related SAEs and only one cases of a lack of viral suppression following re-treatment in the interruption arm. In regards to viral load, we have observed mean rises in viral load from 50 to 5098 copies/ml during 2 week TIs (n=21), 50 to 118,606 copies/ml during 4 week TIs (n=21) and 50 to 65331 during 6 week TIs (n=20). Interestingly, if we analyze viral loads at 2, 4 or 6-week intervals of interruption in sequence (between TIs containing similar amounts of time off therapy), they do not show a trend to increase between similar time-points. For example, the mean viral load at 4 weeks of interruption between three successive interruptions is respectively 118,606 (n=21), 49,761.8 (n=20), and 32,986.1 (n=17). Taken together, data support that sequential treatment interruption does not increase non-response to therapy nor increases viral rebounds.

J. Outcomes on Viral Resuppression upon Retreatment and Emergence of Viral Mutations.

Viral replication has been re suppressed to <400 copies/ml within 8 weeks following re-initiation of therapy after interruptions of 2, 4 or 6 weeks while <16 weeks has been required to achieve <50 copies/ml. Based on this data we chose a fixed interval of 16 weeks between each therapy interruption period in the protocol. Viral mutations associated with treatment resistance were assessed at the first viral load >1,000 copies/ml time point after sequential TI and the last >1,000 copies/ml time point after re-initiation of treatment. Of 24 TIs studied with 67 collective time-points analyzed to date, the lamivudine-associated M184V and the K219E mutations have been detected during sequential TIs in patients on lamivudine-containing regimens while K103N has been detected in Efavirenz- and Nevirapine-containing regimens. Interestingly, the detection of M184V and K219E mutations have been transient, mutations are not detected in subsequent interruptions, suggesting an overgrowth by the wild type virus. This

interpretation is best exemplified by subject S23, where mutations were detected at the 2-week STI against drugs that were not in his current regimen and were not subsequently detected at the subsequent 4 or 6 week STI. The detection of the K103N was detected in association with poor adherence while on therapy in subjects under continuous treatment and in one case after therapy interruption. To date, the history of drug exposure has limited the ability to answer whether STIs accelerate viral resistance as opposed to amplify archived sequences. We expect our present study on therapy naïve subjects receiving lamivudine and a series of short STIs to provide the answer to this question.

K. Outcomes on Immune Reconstitution: CD4 T-cell Count Changes

To date, we have observed a viral rebound in two thirds of subjects during the first 2-week interruption. No significant drop in CD4 cell count has been observed upon treatment reinitiation. CD4 cell counts have risen following re-initiation of therapy (analyzed as a whole or taking all 2 week, 4 week and 6 week STIs as single groups, Wilcoxon Rank Sums, all $p>0.05$). In our ongoing STI trial, an overall analysis between initial baseline and the baseline of the 4th STI (the final comparison interruption of the study, n=17) shows a mean change from 630.8 to 627.8 cells/ul (33.3% to 30.8 CD4 %) in spite of fluctuations of CD4 cell count during previous STIs from 658.4 to 599.9 (33.6 to 32.9 CD4%) at the end of 2 week STI, 657.1 to 542.3 (33.0 to 30.8 CD4%) in the 4 week STI, and 676.6 to 511.2 (33.1 to 29.1 CD4%) in the 6 week STI. Our data supports that changes in CD4 cell counts observed after treatment interruption can be largely explained by redistribution.

L. Outcomes on Immune Reconstitution: CD4 T-cell Recall Responses.

We have observed that treatment interruption in chronically suppressed patients can result in the increase of CD4 and CD8 HIV-specific responses following 4 or 6 week duration STIs which we now interpret as associated with antigenemia and immune reconstitution-related ability by the host to amplify recall responses as it is not significantly correlated to the degree of viral suppression off therapy in our cohort. In accordance with the results of the SSIT study[29, 30], we have also documented increases in IFN-γ secreting HIV-specific CD4 and CD8 T-cell responses in subjects with four sequential therapy interruptions, but no difference ($p>0.05$) in viral replication when comparing viral set-points (three sequential viral loads within 6 weeks with less than 0.3-5 log difference) to a randomized control group that interrupts only once. The retained ability to expand recall responses is also supported by the retention of recall responses against Candida sp and CMV throughout the follow-up period. Importantly, only 1 of 21 subjects showed a transient loss of the Candida sp recall response (SI<3) during the second of four interruptions, supporting the hypothesis that short therapy interruptions are not associated with a loss of immune reconstitution and the host's ability to expand memory responses and combat pathogens. These observations together with the recovery of CD4 T cell counts on therapy justify our focus to determine whether subjects will be

able to maintain the perceived benefits of immune reconstitution in time while decreasing exposure to drugs through short therapy interruptions cycles.

M. Outcomes on Immune Reconstitution: T-cell subset changes.

We have assessed T-cell immunophenotype at entry for the expression of a set of cell surface markers associated with disease progression (CD4/CD45RA), immune activation (CD95, TNFRII, HLADR), and function (CD28) which are known to be modulated following long-term viral suppression and immune reconstitution[31]. A history of viral suppression to <400 copies/ml was associated with a significant increase in $CD4^+/CD28^+/HLA\text{-}DR^-$ (Wilcoxon Rank Sum, p=0.047) and decreases in $CD8^+/CD28^+/HLA\text{-}DR^+$ (Wilcoxon Rank Sum, p=0.016) and $CD3^+/CD95^+$ (Wilcoxon Rank Sum, p=0.047) T-cell subsets as compared to long-term viremics. Interestingly, monitoring T-cell changes during 2-4-6 week STIs revealed the reversal of significant (p>0.05, Wilcoxon Rank Sum) increases in CD38 and HLA-DR expression on T-cell subsets during therapy interruption to baseline levels once viral replication was <50 copies/ml. Due to our experience in analysing the expression of these molecules and to (1) document immune reconstitution changes associated with viral suppression, (2) control for immune activation changes that may be unrelated to viral replication, and (3) assess the overall relation between immune activation and viral suppression, in the present application we propose to measure T-cell phenotype data on activation (CD38, HLA-DR) and functional (CD28, CD45RA/CD62L) molecule expression.

N. Outcomes on Quality of Life: Symptoms-Related Questionnaires.

A questionnaire (modified from ACTG Symptoms Distress Module, MOS-HIV Health Survey) was administered to a subset of control and STI patients to evaluate the impact of therapy interruption on the patients' quality of life (drug toxicity). A preliminary analysis of the answers based on a sample of 19 participants indicated that the STI approach is well tolerated, with patients interrupting treatment (n=12) reporting better overall well being (86.5% reporting excellent to very good health, as compared to 54.5% of patients on continuous treatment at corresponding time points), and lower incidence of G.I.-related symptoms, such as diarrhea (14% in STI patients vs. 35% in controls) and bloating (41% vs. 63%).

O. Response to Rabies Vaccine in HIV positive individuals.

The available data in regards to rabies vaccination in HIV^+ patients focus on post-exposure vaccination, which is usually carried out with 5 subcutaneous injections (1ml IMI injections given immediately after exposure, then Day3,7,14 and 28), unlike the pre-exposure vaccination (3 doses IM) as proposed in our experimental plan. In general, it has been reported that patients with CD4 counts < 200 cells/mm^3 do not respond well to rabies vaccination (Tantawichien et al., Clin. Inf. Dis. 33:122, 2001) which is not applicable to our cohort based on entry criteria off therapy above this threshold. Indeed, Jaijaroensup et al. (Clin. Inf. Dis. 28:913, 1999) confirmed that HIV^+ patients with CD4

counts higher than 300 cells/mm^3 maintained significant titers of neutralizing Ab for 90 days post vaccination indicating that the anticipation that patients on therapy in our study will respond to vaccination is sound. Additional studies in children infected with HIV report differential responses between subjects with high and low CD4 percentages in support of observations on adults (Thisyakorn et al. Clin. Inf. Dis. 30:218, 2000). Specifically, protective Ab titers (≥0.5 IU/ml) could be detected in HIV$^+$ children with CD4 >15% at the 90 day time point, and the only patients that failed to respond to the vaccination were in the <15% CD4 group. In summary, because our patients will be selected based on a CD4 count > 200/mm^3, and will be vaccinated following a minimum of 16 weeks after reaching suppression to <50 copies/ml on ART, we expect that responses to the vaccination will be detected in most individuals. However, in anticipation that detection of memory responses could decay over time, we have added a booster vaccination to the study design at the end of follow-up to amplify our detection of differences between groups if present.

P. Metabolic risk and ART interruption

A number of large multicenter clinical trials have been recently conducted to assess the safety and effectiveness of drug-sparing strategies based on periodic interruption of ART. Preliminary results from the Strategies for Management of Anti-Retroviral Therapy Trial [SMART (http://www.smart-trial.org/default2.htm)] indicate that interrupting subjects were at an increased risk of disease progression (relative risk of 2.15) or AIDS-related events (relative risk of 5.82), as well as cardiovascular AE (relative risk of 1.62) [32] . This observation is partially supported by results from the Development of Anti-Retroviral Therapy in Africa trial [DART (http://www.ctu.mrc.ac.uk/dart/default.asp)], which found an increased rate of HIV-related OIs (8.6 events per 100person years in the interrupting arm, as opposed to 2 in the continuous treatment arm), but an overall *lower* rate of adverse drug events (ADEs) in the interrupting arm (see DART press release, March 1, 2006 on the study's web site). While the increased incidence of HIV-related OI is not unexpected given the design of these trials, the increased rate of cardiovascular AE was unexpected, as ARV medications, in particular the PI class, are known to induce increased LDL cholesterol and triglycerides, as well as lipodystrophy, known risk factors for cardiovascular disease. That is, drug discontinuation would have been expected to reduce rather than increase the cardiovascular risk. However, observational data have also suggested a protective effect of HAART on cardiovascular risk (Bozzette SA, Ake CF, Tam HK, Chang SW, Louis TA. Cardiovascular and cerebrovascular events in patients treated for human immunodeficiency virus infection. N Engl J Med 2003;348:702-710)

For the purpose of clinical management, cardiovascular risk factors have been recently grouped into the so-called "metabolic syndrome." This is characterized by abdominal/trunk obesity, atherogenic dyslipidemia (high LDL cholesterol, low HDL cholesterol, high triglycerides), hypertension, insulin resistance or glucose intolerance, a prothrombotic state (e.g., high fibrinogen or plasminogen activator inhibitor–1) and a proinflammatory state (e.g., elevated C-reactive protein). HIV viremia results in increased endothelial activation, as measured by serum levels of von Willebrand factor

(vWF) and tissue-type plasminogen activator (t-PA) [33], increasing the risk for cardiovascular AE (see also reviews [34, 35]). In addition to soluble factors, cardiovascular risk is inversely associated with the number of circulating endothelial precursor cells [36-38], characterized by the surface expression of CD34, CD133, VEGF-R2 and vWF.
Based on this evidence, we postulate that the increased risk for cardiovascular AE observed in ART interrupting patients is related to a combination of a pre-existing dyslipidemic state with an acute raise in viremia-driven inflammatory and prothrombotic soluble factors. We therefore hypothesize that the occurrence of a cardiovascular AE during ART interruption can be predicted by A) the existence and extent of "metabolic syndrome" in the patients while on treatment and B) the intensity of the inflammatory state observed in the early viremic phase of the ART interruption. We will test this hypothesis by assessing lipid profile, insulin resistance, and a series of inflammatory and endothelial activation markers (vWF, Apo-Lipoprotein B, VCAM-1, Thrombomodulin, tPA, TNF-α, ICAM-1 and IL-6, see section XI.A.). To more accurately predict the risk of cardiovascular events, we will also monitor the number of circulating endothelial progenitor cells (see section XI.D).

III. Study Design

A. Protocol overview

This Protocol is centered on addressing virologic, immunologic and therapy-related toxicity outcomes of initiation and cyclic intervals of treatment interruption in a randomized, non-blinded, "intent to treat "analysis study in subjects naïve to treatment. A total of 52 HIV-1 infected subjects naïve to therapy with CD4 T-cell counts between 200-350 cells/mm^3 and treated with lopinavir/ritonavir, lamivudine and stavudine will be needed to respond to therapy with VL <50 copies/ml (+ time required to get to <50 copies/ml as a maximum of 24 weeks where required) and a CD4+ count > 450 cells/mm^3 (inclusive of rabies vaccination as a de novo antigen starting at week16 of the 24 week period at <50 copies/ml) for randomization. Subjects will be randomized to either (1) three successive treatment interruptions of 2, 4 and 8 weeks, each interruption followed by 16 weeks of therapy, or (2) uninterrupted HAART. Our strategy of cycles of 2-8 weeks off therapy followed by 16 weeks on therapy leads up to a maintenance strategy (8 weeks off, 16 weeks on) decreasing drug exposure by 33%. Primary analysis will be an "intent to treat analysis" and will address the hypothesis that intermittent treatment is not inferior to continuous therapy over the same period, as defined by the proportion of observations during which an individual's CD4 count is above their entry CD4 count. Secondary analysis will also address sustained immune response to rabies (de novo antigen), an indication of the host's retained potential to initiate and sustain responses gained under the benefits of immune reconstitution, and a booster response to rabies on therapy as an additional correlate of the retention of responses between groups. We also hypothesize that no difference in the frequency of "on treatment" viral suppression or in CD4 cell count differential from pretreatment levels will be present between the two groups. Specifically, functional end-points of retained immune reconstitution will be evaluated in conjunction with suppression <400 copies/ml and

retention of CD4 cell count above baseline at the final observation, when both arms are on therapy. During study, re-initiation of therapy will occur any time a CD4 cell count drops below 350 cells/mm^3 off therapy or evidence of clinical disease progression is observed such as a >15% drop from entry CD4 count. Criteria for interrupting therapy will be a CD4 count >400 and viral load <5,000 copies/ml. An NIH-sponsored Data and Safety Monitoring Board will also monitor safety outcomes and patient follow-up at yearly intervals during the study. Differences in immune reconstitution following randomization between groups will be assessed on treatment by evaluating CD4 cell count, changes in cell surface expression of activation markers, and response to recall antigens. The sample size proposed is also powered to test moderate effect sizes on whether the STI arm achieves a lower frequency of therapy-associated toxicities.

HAART is known to cause body fat re-distribution, hyperlipidemia and reduced insulin sensitivity [26-28]. The methods and analysis have therefore been designed to measure all these aspects of the metabolic effects of HAART and to also assess the ability of treatment interruption to attenuate these effects. A secondary aim of this project will be to measure the incidence of HAART-associated lipodystrophy in African subjects. Initial studies by Dr Ian Sanne and colleagues have suggested that while present, lipodystrophy may be less common in African subjects on HAART than Europeans. However, it is possible that this finding is a consequence of limited follow-up and the present study will be able to address this question via extensive follow-up over a 3-year period. While little is known regarding the effect of HAART on body fat re-distribution and its metabolic sequelae in this population, it is known that African subjects tend to be more insulin resistant than Europeans but to have a less atherogenic lipid profile [16-18, 40]. This may lead to more diabetes and hypertension and less ischemic heart disease. The consequences of HAART on insulin sensitivity and ß-cell function will be carefully studied by monitoring the serum levels of insulin, C-peptide, proinsulin and glucose. Indeed, one of the major potential advantages of short-term therapy interruptions, irrespective of retained virological or immune benefits, is the possibility that the time off therapy results in lower rates of accumulated chronic toxicites. These benefits may not be seen immediately, but are more likely with increasing time on therapy. We will assess metabolic variables between arms to include: insulin, proinsulin, C-peptide, glucose, total cholesterol, HDL and LDL cholesterol - and triglycerides. The anthropometric variables that will be measured are weight, height, peripheral fat (DEXA scan at start of study), abdominal fat (MRI scan at start and end of follow-up), waist-to-hip ratio and skinfold thicknesses. Bone mineral density will also be analysed using DEXA scans. Virological outcomes will be measured by viral load and viral genotyping at each rebound during STI and if viral load is not decreased by 1 log after 12 weeks of re-initiated therapy.
All laboratory testings will be available to the study personnel, and can be used to inform clinical decisions in regards to subject monitoring, AEs and other clinical management issues

IV. SELECTION AND ENROLLMENT OF SUBJECTS

A. Study Site, Population and Recruitment

The participants to be recruited are expected to reflect the demographics of South Africa based on previous recruitment history at the site. Specifically, the patient population is expected to be 82.9% black participants, 10.5% white, 2% Asian, 4.4% other, 65% women, 30.3% between 18-30 years of age, 64.7% between 30 and 50, and 4.8% older than 50. The numbers of HIV positive individuals in each age group and demographic population varies, therefore recruitment is anticipated to be representative of the HIV prevalence in each group.

B. Source of Study populations

In addition to the University of Witwatersrand health system, participants will be identified for referral to the study at one of two clinical sites where research is active: 1) The HIV Clinic of the Johannesburg General Hospital where over 3000 patients are followed and 10 newly identified patients are entered into the clinic weekly, 2) The Helen Joseph Hospital where over 2000 patients are followed and 10 newly identified patient are entered into the clinic weekly. At all of the clinics CD4 cell count testing is a routine, with patients who would be eligible for clinical trials or antiretroviral therapy having access to viral load testing. At each of the two academic institutions there is an active primary health care clinic for HIV positive patients. Active VCT and MTCT prevention programs at each of the hospitals identify HIV positive women and their partners. Positive patients are followed in the primary health care clinics.

C. Inclusion of women and disadvantaged communities:

This protocol addresses the considerations of the NIH relevant to human subjects. Recruitment of subjects will be focused on disadvantaged communities in a resource poor setting. The subjects will be predominantly recruited from the public sector in a country where the majority of the population is black. The Clinical HIV Research Unit has recruited predominantly women (60%) to antiretroviral therapy studies completed to date. This trend will continue as women are more likely to present for voluntary counseling and testing in mother to child transmission prevention programs. The participants to be recruited are expected to reflect the demographics of South Africa based on previous recruitment history at the site.

D. Inclusion on children:

We will recruit subjects aged >18 years. Therefore, children, defined as “individuals under the age of 21 years,” (NIH requirements for human subjects) will be included in this project. Along with the other inclusion criteria outlined in the application, individuals who are 18 years of age or over will be encouraged to participate. Individuals younger than 18 years will not be enrolled for multiple reasons. Our limited sample size, limited knowledge of the safety of therapy interruption in adults and the requirement for

very specific conditions requiring recruited participants to alternate times on and off therapy would limit our ability to interpret data from children if different from adults. While therapy interruption in children under 18 years of age is scientifically relevant to their disease management, it is beyond the scope of this proof-of-concept project and should be investigated separately.

E. Inclusion Criteria

1. HIV-1 infection, as documented by any licensed ELISA test kit or Determine® rapid test; and confirmed by plasma HIV-1 RNA at any time prior to study enrollment. HIV-1 culture, HIV-1 antigen, or a second antibody test by a method other than the technique used in the first ELISA is acceptable as an alternative confirmatory test. For source documentation of Determine® rapid testing a signed note by a qualified HIV testing nurse or a doctor is acceptable.
2. Age greater than ≥18yrs
3. Presenting with one occurrence of CD4+ cell count 200-350 cells/mm^3 in the absence of therapy obtained within 60 days of initiating study antiretroviral therapy.
4. Antiretroviral naïve with the exception of post-exposure prophylaxis and short course therapy for mother to child transmission. The previous exposure may not exceed 6 weeks of any nucleoside analogue reverse transcriptase inhibitors, and/or a protease inhibitors or/and a non-nucleoside analogue reverse transcriptase inhibitors therapy.
5. Subjects must be willing to abstain from all immunomodulatory drugs (defined below) during the study period.
6. Subjects must be willing to adhere to the treatment, drug interruption schedule, HAART re-initiation regimen and schedule according to the study protocol. The latter confirmation of willingness will not impact the patients' right to withdraw from the study at any point
7. Negative serum or urine pregnancy test within 3 days before initiating antiretroviral therapy. In addition, subjects must agree not to participate in a conception process (e.g., active attempts to become pregnant or to impregnate, sperm donation, or in vitro fertilization), and if participating in sexual activity that could lead to pregnancy, the female subject and partner must use a form of contraception as listed below while on study drugs and for 60 days after stopping study drugs. Female subjects without reproductive potential (i.e., have reached menopause or undergone hysterectomy, bilateral oophorectomy, or tubal ligation) or female subjects whose sole male partner has undergone successful vasectomy with documented azospermia or has documented azospermia for any other reason, are eligible without requiring the use of contraception. NOTE: Subject-reported history is acceptable documentation of sterilization (hysterectomy, bilateral oophorectomy, tubal ligation, or vasectomy). At least one of the following methods must be used appropriately with or without a hormonal-based method: Condoms (male or female) with or without a spermicidal agent. Condoms are recommended because their appropriate use is the only contraception method effective for preventing HIV

transmission. Diaphragm or cervical cap with spermicide. An IUD is an adequate method of birth control, but increases the risk of pelvic inflammatory disease.

8. Ability and willingness of subject or legal guardian/representative to give written informed consent.
9. Subjects should be available for follow-up for a minimum of 3.5 years.
10. Laboratory values [Grade ≤ 2] obtained within 45 days prior to study entry.

    - liver enzyme (AST,ALT,GGT) < 5 times upper limit of normal
    - Hemoglobin, neutrophil count or platelet count must be <grade 3 (ACTG toxicity grading)

F. Exclusion Criteria

1. Patients who have a history of an AIDS-defining illness (CDC category C) with the exception of a history of pulmonary Tuberculosis.
2. Presence of a newly diagnosed AIDS-defining (CDC classification C) opportunistic infection or condition requiring acute therapy at the time of enrollment. Active tuberculosis at baseline will require anti-tuberculous treatment for 2 months with negative sputum smear/or if extra-pulmonary then considered by the clinician to be responding to treatment, before enrollment. Delayed enrollment will require re-screening once 2 months of TB treatment has been completed. (In line with the WHO consensus guidelines for ART treatment in a resource poor setting.)[24]
3. Previous therapy with agents with significant systemic myelosuppressive, neurotoxic, pancreatotoxic, hepatotoxic or cytotoxic potential within the previous 30 days.
4. History of immunomodulatory therapy within the last 4 weeks of screening, but not limited to: systemic corticosteroids; systemic cancer chemotherapy/irradiation; cyclosporin; tacrolimus (FK-506); OKT-3; any interleukin, including IL-2; any interferon; cyclophosphamide; methotrexate; IVIG (gamma globulin); G/M-CSF; hydroxyurea; thalidomide; pentoxifylline; thymopentin; thymosin; dithiocarbonate; polyribonucloside.
5. Prior history of Rabies vaccine inoculation
6. Active alcohol or substance abuse that in the opinion of the investigator will prevent adequate compliance with study therapy or to increase the risk of developing toxicity complications.
7. Intractable diarrhea (>6 stools/day for seven consecutive days) within 30 days prior to study entry.
8. Pregnancy or breast feeding at screening
9. Proven or suspected acute hepatitis within 30 days of study entry. Chronic active hepatitis as documented with Hepatitis B eAg, or sAg positive serology OR Hepatitis C positive PCR. The presence of Hepatitis B surface antibody positive is acceptable for inclusion in the study.
10. Signs or symptoms of bilateral peripheral neuropathy of grade 2 or greater at the time of screening.
11. Inability to tolerate oral medication

12. Any clinical condition that in the opinion of the investigator would make the subject unsuitable for study or unable to comply with the dosing requirements.

V. Regimens, Administration, and Duration

During this study the provision of the antiretroviral therapy regimen will be from donor funding for the duration of study over 3.5 years. Subjects on study drug whether in or out of protocol, will be followed clinically. Study subjects that can not remain on study drug will be monitored quarterly for criteria of post-study provision through the National ARV Roll-out Plan of the Department of Health. Provision of treatment will be provided by the National Roll-out Plan.

The choice of treatment regimens will be guided by principals of treatment including but not exclusive to:

• Southern African HIV Clinicians Society Guidelines for the use of antiretroviral therapy in a resource poor setting, both adults and children

• Registration or submission for registration of the medicines by FDA and South African Medicines Control Council

• Access to drugs for on trial and post-trial treatment including access to an alternative regimen of AZT, didanosine and Efavirenz administered continuously for patients who demonstrate virologic failure.

• Community access to treatment after the completion of the study – facilitated by the use of access priced or locally manufactured drugs

Preferred regimens are presented below.

The study regimen stavudine (D4T) 30-40mg bid, lamivudine (3TC) 150mg bid, and lopinavir/ritonavir 400mg/100mg bid. The treatment should be taken with a meal to ensure optimal absorption of the lopinavir/ritonavir. The dose of D4T is modified according to weight, for weight < 60kg the dose is 30mg bid, for weight >60kg the dose is 40mg bid. Treatment is initiated and continued for six months. Then patients who have maintained undetectable viral load and are stable on therapy will be randomized to study groups.

A Treatment switch will be allowed for toxicity related intolerance of D4T to AZT 300mg bid where required.

A: Product Formulation and Preparation

Table 2 : Product Formulation and Storage Requirements.

MEDICATION	FORMULATION	STORAGE
Zidovudine	100 mg capsules<br>250 mg capsules<br>300 mg tablets<br>50 mg/5 ml syrup	Store at 15°-30ºC (59° - 86°F) and protect from light
Lamivudine	150 mg tablets	Store at 15°-30ºC (59° - 86°F) and protect from light
Lamivudine	5 mg/ml oral solution<br>10 mg/ml oral solution	Store at 15°-25ºC (59° - 77°F) and protect from light
Stavudine	20 mg capsule<br>30 mg capsule<br>40 mg capsule<br>1 mg/ml powder for oral solution	Stavudine capsules and powder for solution should be stored at room temperature, not to exceed 25°C, in tightly sealed containers, protected from light. After constitution, store Stavudine solution in tightly sealed bottles under refrigeration (2-8°C) for up to 30 days.
Lopinavir/RTV (LPV/r) (Kaletra®)	133.3 mg lopinavir/33.3 mg ritonavir soft gel capsules and oral solution 80mg lopinavir and 20mg ritonavir per ml.	Store at 2°-8°C (36°-46°F). Avoid exposure of Kaletra capsules and solution to excessive heat. Subjects should be instructed to store LPV/r capsules and solution under refrigeration. Once brought to room temperature (up to 25°C [77°F]), LPV/r capsules and oral solution should be used within 42 days.
Lopinavir/RTV (LPV/r) (Aluvia)	200mg lopinavir/50mg ritonavir film-coated tablet.	Store at 15°-30°C (59°-86°F).

Table 3. Study product dosing

Study Population	Product	Dosing
Study regimen for adults >18 years	Stavudine	Adults $\geq$ 60 kg: 40 mg twice daily<br><br>Adults < 60 kg: 30 mg twice daily
	Lamivudine	Adults: 150 mg twice daily
	Lopinavir/ritonavir	Adults: 400/100mg twice daily
Alternative substitution for Stavudine	Zidovudine	Adults 300mg BID

Description of Rabies Vaccine (Aventis)

The Verorab® vaccine (Purified Vero Cell Vaccine, PVCR) is a freeze dried vaccine, one vial contains 1 immunizing dose. One immunizing dose has a protective activity equal to or greater than 2.5 international units. The PVCR rabies virus used is the Wistar rabies PM/W138-150303M strain. Diluent: Solution of 0.4% sodium chloride 0.5ml is supplied. Once reconstituted to a pale yellow solution, the injection of vaccine is given by intramuscular route strictly.

Side effects and special precautions include minor local reactions like redness and slight induration at the injection site. Rare febrile reactions occur.

B. Product Supply, Distribution, and Pharmacy

1. Study Product Acquisition

The study drugs for this study, Zidovudine, Lamuvidine, Stavudine, and Lopinavir/ritonovir, will be acquired in the form of brand name drugs from the pharmaceutical companies distributors. Verorab® will be supplied from Aventis South Africa directly or via their distributor. The products will be received by the Pharmacist Coreen Barker, who is responsible for the maintenance and storage of study drug,

dispensing and retention of returns. Logs will be maintained according to the SOP's of the pharmacy.

2. Study Product Accountability

The study pharmacist is required to maintain complete records of all study products received from the whole sale distributor appointed by the department of health, or the pharmaceutical company sponsoring study drug, and subsequently dispensed. All unused study products will be retained at the site. Dispensed returns will be destroyed according to the destruction SOP of the pharmacy. The procedures to be followed are provided in the manual, Pharmacy Guidelines in the section Study Product Control.

Drug labeling for patient use will contain the following information:

- Patient study initials
- Patient Identification Number
- Drug name
- Quantity of tablets
- Batch number
- Expiry Date

3. Study product destruction

All returns will be received by the site coordinators, with counting and accountability completed at the site. Once the site monitors have completed the site visits, and permission has been given by the monitor to destroy the returns, the sites will then submit the drugs for destruction to Sanumed medical supply disposals for destruction. A certificate of destruction will be placed into the site file.

C. Concomitant Medications

To avoid adverse events caused by drug interactions, sites must refer to the most recent package inserts of study drugs and concomitant agents whenever a concomitant medication is initiated or a dose is changed.

Sites must also refer to the most recent study medication's package insert to access additional current information on prohibited and precautionary medications.

1. Required Medications

No concomitant medications are required for this protocol.

2. Rifampicin with Protease Inhibitor

Rifampicin may not be given as concurrent medication in a patient on lopinavir/ritonavir. Patients who require treatment with rifampicin for TB during this study will use Rifabutin instead so lopinavir/ritonovir can still be used.

3. Prohibited and Precautionary Medications

Package inserts of antiretroviral and concomitant agents should be referred to whenever a concomitant medication is initiated or dose changed to avoid drug interaction adverse events.

3.1 Agents prohibited with lopinavir/ritonavir (Table 4):

**Agent by Class**	**Prohibited Concomitant Agents with Lopinavir/Ritonovir**
Antiarrhythmic agents	Fleicanide<br>Propafenone
Anti-histaminics	Astemizole
	Terfenadine
Anti-infectives	Rifampcin
Cholesterol lowering agents	Lovastatin
	Atorvostatin
	Simvastatin
GI Motility	Cisapride
Psychiatric Medications	St. John's Wort (*Hypericum perforatum*)
Sedative/hypnotics	Midazolam
	Triazolam
Other	Dihydroergotamine
	Ergonovine
	Ergotamine
	Methylergonovine

3.2 Agents Prohibited Medicines with Ritonavir (table 5):

**AGENT BY CLASS: DRUG NAME**	**PROHIBITED CONCOMITANT AGENTS WITH RITONAVIR**
ANTIARRHYTHMIC AGENTS	AMIODARONE

AGENT BY CLASS: DRUG NAME	PROHIBITED CONCOMITANT AGENTS WITH RITONAVIR
	FLEICANIDE<br>PROPAFENONE<br>QUINIDINE
ANTI-HISTAMINICS	ASTEMIZONE<br>TERFENADINE
CALCIUM CHANNEL BLOCKER (CARDIAC)	BEPRIDIL
GI MOTILITY	CISAPRIDE
SEDATIVE/HYPNOTICS	MIDAZOLAM<br>TRIAZOLAM
OTHER	PIMOZIDE<br>ERGOT DERIVATIVES

3.3 Precautionary Medicines with Lopinavir/ritonavir

Precaution may be required for the following medicines:
Dose adjustment is required for combinations of Lopinavir, Ritonavir with the following medicines:

- Rifabutin: reduce the dose of Rifabutin to 150 mg 3 times a week.
- Methadone: AUC decrease by 53%; monitor for withdrawal.
- Ketoconazole: do not exceed a maximum dose of 200 mg daily.
- Oral contraceptive: ethinyl estradiol AUC is decreased by 42% rendering this form of contraceptive potentially ineffective. Do not use oral contraceptives with Lopinavir, Ritonavir combination; an alternative contraception should be discussed with the patient.
- Sildenafil levels increased; do not exceed 25 mg/48 hours.
- Anticonvulsants: Anticipate a large decrease in the LPV levels and monitor the anticonvulsant level.
- Atovaquone: levels may decrease and dose adjustment is required.

3.4 Precautionary medicines with DDI, D4T:

- When INH is prescribed with DDI or D4T, pyridoxine will be administered.

In addition to the prohibited medications, use of the agents listed below while on study, may require additional monitoring of drug levels for adverse events. These PRECAUTIONARY MEDICATIONS include, but may not be limited to:

Immunomodulating agents:
Immunomodulating such as systemic corticosteroids, interleukins, thalidomide, interferons, cytotoxic chemotherapeutic agents, and radiation therapy (with the exception of local KS treatment) should be avoided where possible. Alternatives for these medications and/or therapies should be considered. The above-mentioned medications and/or therapies, as well as other medications and/or therapies, can be used when a medical indication necessitates the use of these, and if no alternatives are available

VI. CLINICAL AND LABORATORY EVALUATION

A. Consent procedure

Patients identified as eligible for screening in this study will be provided with education on HIV and treatment benefits. The consent procedure will be according to the consent SOP of the Clinical HIV Research Unit. During consent process, a detailed understanding of study procedures and anticipated follow-up and visits will be reviewed with each subject.

B. Screening procedure Week – 16 visit (prior to treatment initiation) (I. Sanne, M. John, C. Firnhaber)

A symptom-directed history and physical exam will be documented at the time of screening. These will be repeated on the day of treatment initiation and monthly thereafter. Past history including HIV-related and non-HIV-related diagnoses, prior anti-HIV therapies, immunomodulatory therapies and vaccines, current prescription medications and lab reports concerning year of diagnosis and available viral loads or CD4 cell counts will be obtained. History of opportunistic infections will be obtained. After successful screening and consent, subjects who meet all the inclusion and exclusion criteria will begin their triple combination therapy after completion of a baseline DEXA and MRI scans. A visit window of up to +/-30 days from treatment initiation to the baseline DEXA and MRI scans is permissible.

C. Prior and concomitant medication:

Concomitant administration of therapies listed in the exclusion criteria is prohibited throughout the trial. Other than these immunosuppressive agents, particular attention will be given to avoid treatments which are neurotoxic or immunizations other than rabies at

16, 17 and 22 weeks of therapy. A full list of prohibited concomitant medications for each of the three drugs will be available to the investigator.

D. Documentation of antiretroviral therapy:

Antiretroviral therapy prescribed will be listed on antiretroviral therapy pages in the CRF.

E. Treatment initiation visit (enrollment) (-14 to -12) weeks

Treatment initiation visit (enrollment) can occur from 2-4 weeks after the screening visit.. The visit procedures at this time including the collection of metabolic samples and radiology including radiology visit window of +/- 30 days from the treatment initiation visit. Fasting lipogram will be performed before or at drug initiation. The time to achieving <50 copies/ml is anticipated to occur within 12 weeks of starting therapy thus this visit is identified as –14 to-12 weeks to a week 0 representing a viral load <50 copies/ml. However, week 0 will be assigned to the first viral load documented at <50 copies/ml after initiation of therapy with a maximum time of 24 weeks given to each subject to achieve this level of suppression before discontinuation from the study due to therapy failure. If failing to suppress, subjects will be referred for antiretroviral therapy through alternative programs of the Clinical HIV Research Unit or National Roll-Out Plan for Antiretroviral therapy. All visits will be performed with a tolerance of ±7 days to allow for scheduling problems.

F. Follow-up period weeks -8 weeks to 0 weeks:

During this period the patient will be required to visit the site at week -8, and 0 for the collection of safety blood specimens, CBC/CD4+, PBMC's, and metabolic blood specimens. The anticipation is that the majority of patients recruited will achieve an undetectable viral load <50 c/ml by the 0 week visit. Once undetectable the patient is now completely recruited onto the study and followed for an additional period of 24 weeks with an undetectable (<50c/ml) viral load. Randomization takes place at week 24 after the first viral load <50 copies/ml. Should the patient not achieve undetectable viral load at week 0, an additional window of 12 weeks is allowed for patients to achieve undetectable viral load. During this period a repeat week 0 visit may be conducted or subject will be discontinued as already described in section VI. E describing the –12 week visit. All visits will be performed with a tolerance of ± 7 days to allow for scheduling problems.

G. Follow-up period weeks 0 to +24

During this period the patients are monitored for the safety and adherence to antiretroviral therapy. The visit schedule is designed to include the monitoring of treatment at week 8, 16 and 24, and the initiation of rabies vaccines at weeks 16, 17, and 22. During the required 24 weeks of maintaining viral load under 50 copies/ml, subjects will be removed from study if viral load is >400 copies/ml on more than two sequential visits more than 2 weeks apart. Any viral load >400 copies/ml will require a repeat viral load more than 2 weeks later. Discontinued subjects due to therapy failure will be referred for alternative treatment as described in section VI. E. Where the subject's viral was detectable during the period between 0 and +24 weeks, randomization to a treatment arm may only proceed if the viral load <50c/ml and CD4 count is >450 at week +24 visit. Monitoring blood work will be done at week 22 for week 24 randomization (see SOE). An 8 week extension of the 0-+24 week period is permitted to achieve <50 c/ml provided that the subject has not demonstrated viral loads in excess of 400c/ml on two visits. An 8 week extension of the 0-+24 week period is permitted provided that the subject has not demonstrated CD4 count >450 at week 24. All visits will be performed with a tolerance of ± 7 days to allow for scheduling problems.

H. Additional Inflammatory Markers

Upon entry into the study, baseline visit, randomization visit, before and after treatment interruption visits and exit visit there will be an additional assays performed on stored plasma samples (no additional blood draws will be required.) Inflammation markers associated with cardiovascular risk will be measured by ELISA and will include: Von Willebrand Factor (vWF), Tissue Plasma activator (t-PA), Interleukin -6 (IL-6), Tumor Necrosis Factor-a (TNF-a), Vascular Cell Adhesion Molecule-1 (VCAM-1) and Intercellular Adhesion Molecule-1 (ICAM) ELISA testing will be performed at the Wistar Institute. Additionally, we will evaluate the number of circulating endothelial precursor cells in the peripheral blood by flow cytometry.

I. Off treatment/ On study follow-up

Patients who, for any reason, stop taking study medications, will continue to be followed-up by the investigators at the Clinical HIV Research Unit until the end of the study period (3.5 years); unless the patient withdraws consent to remain in the study. Clinical visits, laboratory evaluation (CD4 count and viral load) and assessment of work ability and performance analysis will be completed at each of the quarterly scheduled visits.

J. Rabies Vaccination Schedule

The primary outcome will be defined by de novo responses to rabies following inoculation with the VERORAB® (PVRV) vaccine. Rabies vaccinations have been shown to be immunogenic in HIV infected subjects [41-43]. Specifically, once an undetectable viral load <50 c/ml has been achieved, PVRV will be administered to study subjects on

weeks 16, 17 and 22 of this <50 copies/ml suppression period. A booster PVRV injection will also be administered at week 92 (controls and experimentals). The intramuscular pre-exposure injection method will be used since it is known to result in higher neutralizing antibody titers [44]. PBMC and serum will be collected as described for determination of lymphoproliferative responses and titers of rabies-neutralizing antibodies [45].

K. Randomization

Criteria for randomization are:

1. a period of 24 weeks with viral load <50 copies/ml at the beginning and end of this period without more than 2 viral loads above 400 copies/ml. Should viral load not be at <50, an additional 8 weeks will be allowed to reach this level before discontinuation from study.

2. a CD4 count at the end of the 24 week period greater than 450 cells/ul. Should CD4 count not be at 450, an additional 8 weeks will be allowed to reach this level before discontinuation from study.

3. a negative pregnancy test.

4. Laboratory values [Grade ≤ 2] obtained within 45 days prior to study entry.

- liver enzyme (AST,ALT,GGT) < 5 times upper limit of normal
- Hemoglobin, neutrophil count or platelet count must be <grade 3 (DAIDS toxicity table)

5. Presence of an active diagnosed AIDS-defining (CDC classification C) opportunistic infection or condition requiring acute therapy.

6. Active alcohol or substance abuse that in the opinion of the investigator will prevent adequate compliance with study therapy or to increase the risk of developing toxicity complications.

7. Proven or suspected acute hepatitis within 30 days of study entry. Chronic active hepatitis as documented with Hepatitis B eAg, or sAg positive serology OR Hepatitis C positive PCR. The presence of Hepatitis B surface antibody positive is acceptable for inclusion in the study.

L. Post-Randomization: Control Arm

After randomization, those individuals randomized to continuation of HAART will be followed every 8 weeks for an additional 72 weeks. HIV-1 viral load, CBC, CD4 cell count test, metabolic tests as well as PBMC/serum sample collection for this group are listed in the table 6 below. During the study period, data on adherence to antiretroviral therapy will be obtained every eight weeks through the use of pill counts. At each visit, data on any changes in clinical status will be obtained. If during this 72 week period HIV RNA rebounds to >greater than 5000 copies/ml, the subject will be evaluated for resistance, more intensive treatment adherence counseling and support will be offered. If viral load remains above> 5000 copies/ml after an additional 16 weeks, subject will be discontinued as a therapy failure and alternative treatment addressed as described in

section VI. E. All visits will be performed with a tolerance of ± 7 days to allow for scheduling problems.

Table 6. Listing of biological sample collection in control group. Example of sample distribution for control group over a projected 96 week time period.

Stage	Week	Rabies Vaccine	CBC/CD4/VL	PBMC/Serum	Metabolic	DEXA	MRI
Screening Visit/Consent	-16		X		X		
Start Tx.	-14 -12		X	X	X	X	X
Tx.	-8		X	X	X		
Tx.	0		X	X	X		
Tx	0 + 8		X	X	X		
Tx	0 + 12		X (viral load only)				
Start of <50 Treatment Period							
Treatment	8		X	X			
Tx.	16	X	X	X	X		
Tx.	17	X					
Tx.	22	X	X	X	X		
Tx	22+8		X				
Tx.	24		X	X	X		
Randomization	26						
Monitor-	32		X	X	X		
Monitor	40		X				
Monitor	48		X	X	X		
Monitor	56		X				
Monitor	64		X	X	X		
Monitor	72		X				
Monitor	80		X	X	X		
Vaccine Booster visit+	92	X	X	X	X (no DEXA)		X
Study end visit	96		X	X	X		

M. Post-Randomization: Experimental Arm

After randomization, those individuals randomized to the interrupting arm will be followed every 4 to 12 weeks for 72 weeks. The schedule for assessment of HIV-1 viral load, CBC, CD4 cell count, metabolic and PBMC/serum sample collection for this group is in the following Table 7. Individuals randomized to the interrupting group will have three sequential interruptions of treatment of 2, 4 and 8 weeks, followed in each case by 16 weeks of therapy. The duration of the first two STIs of 2 and 4 weeks are based on safety considerations, to assess the outcome of a short treatment interruption in each patient before progression to longer 8 week STI.

Safety parameters during ART interruption:
During the 8 week interruption of antiretroviral therapy (ART), CD4 and HIV viral load will be checked at week 4 of the interruption. If the CD4 count is < 350 or the viral load

is a.) >250,000 or b.) >1.5 log above baseline viral load, ART will be restarted immediately. Response to therapy will then be monitored as described below.

Response to therapy re-initiation criteria after any STI:
At 12 weeks on therapy following an STI, patients who fail to re-suppress with viral load decline < 1 log; have a viral load >5,000 copies/ml; and/or a CD4 count <400 will not proceed to interrupt and will be re-tested for these safety criteria after one month. If the re-testing fails to meet these criteria, the subject will be discontinued from the experimental arm and included in analysis as a therapy failure end-point. All visits will be performed with a tolerance of ± 7 days to allow for scheduling problems.

Table 7. Listing of biological sample collection in experimental group.
Sample distribution for experimental group over the projected 96 week time period.

Stage	Week	Rabies Vacc.	CBC/CD4/VL	PBMC/Serum	Metabolic	DEXA	MRI
Screening Visit/Consent	-16		X		X		
Start Tx.	-12		X	X	X	X	X
Tx.	-8		X	X	X		
Tx.	0		X	X	X		
Tx	0 +8		X	X	X		
Tx	0 +12		X	X	X		
Start of <50 Treatment Period							
Treatment	8		X	X			
Tx.	16	X	X	X			
Tx.	17	X					
Tx.	22	X	X	X	X		
Tx.	22+8		X	X	X		
Tx.	24		X	X	X		
Randomization							
Tx	26		X				
Start- 2wk TI	30		X	X	X		
End TI: Start 16 wk. Tx..	32		X	X	X		
Tx (12 wks)	44		X				
Start - 4 wk. TI	48		X	X	X		
End TI: Start 16 wk. Tx.	52		X	X	X		
Tx (12 wks)	64		X				
Start – 8wk TI	68		X	X	X		
Interrupted Tx week 4	72		X				
End TI: Start 16 wk. Tx.	76		X	X	X		
Vaccine booster visit	92	X	X	X	X	(no Dexa)	X X
Study end visit	96		X	X	X		

N. Duration of subject participation

The duration of follow-up between the experimental and control is the same. Both arms will be followed for a period of 96 weeks from viral suppression.

O. Total sampling of blood volumes:

Clinical data regarding viral load, CD4 cell count, CBC, metabolic tests bloods and PBMC/serum collection is summarized by visit in both control and experimental arms in blood draw tables 6 and 7. Time-points listed below are derived from the above referred tables. In summary, a minimum of 5 mls (CD4, CBC and flow cytometric analysis) and a maximum of 90 ml (15 mls for CD4, CBC, viral load and Liver/Kidney tests; 12 mls for insulin and fat metabolism tests, and 64 mls for PBMC isolation) of blood are to be collected at any one time-point with a maximum of 184 mls of blood to be collected within a 3 month interval (12 weeks).

P. Adherence Assessment and Feedback

Adherence will be assessed at each visit during the experimental phase using pill counts for each drug, while on therapy. The proportion of prescribed doses taken will be calculated for each drug separately. Subjects will be required to bring their remaining pills with them to each visit. The study nurse will then count the pills remaining in the presence of the individual. Subjects will then be re-supplied with the subsequent month's refill with 10 days supply excess. If an individual has suboptimal adherence (i.e., <95%), they will be queried as to reasons for missing doses and counseled on how to avoid missing doses.

Q. Primary care provision

The site will provide the current minimum Department of Health standard of care, with the provision of primary and secondary prophylaxis. Patients requiring hospitalization can be referred to Helen Joseph Hospital. At the site medications listed on the in-country Essential Drug List (EDL) will be available (available at http://www.hst.org.za/edl.edlinfo.htm). The site is linked to facilities providing TB program services. Investigation facilities available include x-rays, and full laboratory testing through the NHLS.

R. Pregnancy

Female participants will be monitored for pregnancy by urine test at each visit. Subjects who become pregnant while on study will be monitored by the study team for safety of antiretroviral therapy during pregnancy. Patients on the continuous treatment arm who elect to stay on treatment will be included in study analysis. Patients on the interruption arm will be removed from the arm and if they choose, monitored on continuous therapy for the period of study yet their data will be censored for analysis. Patients who become pregnant will be offered a consent with the option of continuing treatment throughout

pregnancy, interrupting treatment during the first trimester or discontinuing treatment for the pregnancy. All three options will be provided for without discontinuing access to antiretroviral therapy.

S. Concomitant Medications

All concomitant medications taken since the last report will be recorded in the source documentation. For targeted medications (Schedule 2 or more according to the South African Medicines Control Council), the source document must include start dates, stop dates, and dose changes.
Please note that only > Schedule 4 medicine (those requiring a doctor prescription) and any medication used to treat a Grade 3 or more adverse event will be recorded on the CRFs.
Please refer to the most recent study medication's package insert to access additional current information on prohibited and precautionary medications.

T. Medication History

All modifications to study drug(s) including initial doses, patient-initiated and/or protocol-mandated interruptions, modifications, and permanent discontinuation of treatment will be recorded on the CRFs at each visit. Subject-initiated and protocol-mandated interruptions will be identified as inadvertent or deliberate interruptions of study drug(s) dose(s) for≥5 days.

At baseline all current medications and taken within 30 days of the visit will be included as medication history. Nonprescription medications and alternative therapies [and/or dietary supplements], taken within 30 days of entry/since the last clinic visit will be recorded in the source document only. Include actual or estimated start and stop dates. Please note that only prescription medication (South African Schedule 4) will be recorded on the case report forms (CRFs). The medication history as described above is required for the source document.

U. Clinical Assessments

a) Targeted Physical Exam

A targeted physical examination, to be driven by any signs or symptoms previously identified that the subject has experienced within [30 days of entry/since the last visit]. In addition to the above targeted examination, the team may specify other required evaluations in the Schedule of Events as part of the Targeted Physical Exam.

b) Complete Physical Exam

Complete physical examination is required at screening visit and permanent discontinuation visits. A complete physical examination includes the examination of the head and neck, pulmonary, cardiac, abdomen and neurologic examination.

c) Height

Height at entry is required at the screening visit, for the purposes of calculating body mass index.

d) Weight

Weight is required at all visits to the site.

e) Signs and Symptoms

All signs and symptoms will be recorded in the source documentation regardless of severity. However a more limited assessment of signs and symptoms is required in the CRF and database collection.
Any signs or symptoms that led to a change in treatment, regardless of grade, will be recorded on the CRFs.

At baseline, record all Grade 3 or more signs/symptoms. For post-baseline assessments, record all Grade 3 or higher signs/symptoms.
All signs, symptoms, and toxicities will be documented in the subject's record, but only signs and symptoms Grade ≥ 3 will be recorded on the CRFs.
All signs, symptoms, HIV-related and AIDS-defining events, deaths, and toxicities will be documented. in the CRFs within 7 days throughout the course of the study.
Refer to the Division of AIDS Table for Grading Adult Adverse Experiences, which can be found on the Regulatory Compliance Center (RCC) Web site: http://rcc.tech-res-intl.com

f) Diagnoses and adverse events

All confirmed and probable diagnoses made since the last visit will be recorded in the source documentation, including current status at the time of study visit.
Please note that only team-determined diagnoses will be recorded on the CRFs.
For each team-determined diagnosis, the source document must include:

1. date of diagnosis, date of resolution

2. method of confirmation of diagnosis or evidence for probable diagnosis

3. Severity graded as mild, moderate and severe or life threatening

g) Vital Signs

Weight, temperature, pulse rate, respiratory rate and blood pressure collected at all visits.

V. Laboratory Evaluations

Any laboratory toxicities, regardless of grade, will be recorded on the CRFs. Refer to the Division of AIDS Table for Grading Adult Adverse Experiences, which can be found on the Regulatory Compliance Center (RCC) Web site: http://rcc.tech-res-intl.com

i.Hematology:

Hemoglobin, hematocrit, red blood cells (RBC), mean corpuscular volume (MCV), white blood cell count (WBC), differential WBC, absolute neutrophil count (ANC), and platelets.

ii Liver & Kidney Function Tests

Total bilirubin, AST (SGOT), ALT (SGPT), alkaline phosphatase, creatinine, indirect bilirubin, γ-glutamyl transaminase (GGT).

iii Blood Chemistries

U and E Glucose, triglycerides, cholesterol (HDL, LDL, TG), lactate dehydrogenase (LDH), creatinine, total protein, albumin,

iv Urinalysis

A urine dipstix may be used for the estimation of urine protein, glucose and blood. Abnormalities of grade 3 or more, not attributable to menses will be recorded in the CRF.

v. Pregnancy Test and Chest X-Ray

For women with reproductive potential: Urine β-HCG (urine test must have a sensitivity of 25-50 mIU/mL) will be performed at every visit.

Chest X-Ray may be performed at the discretion of the investigator for signs and symptoms related to the chest. Abnormalities on the x-ray will be recorded as diagnosis at each visit.

vi Stored Plasma/PBMC/Serum/Tissue

Samples for plasma and PBMC will be collected as listed in Tables 6 and 7 and described in blood collection section VI.M

VII. Study Treatment Toxicity Management

A. Antiretroviral Therapy Dosage Reductions

Dose modification and treatment modification will be allowed without prejudice to the primary efficacy outcome variable. Dose modification for weight is only required for stavudine in adult patients (<60kg = 30mg bid; >60kg = 40mg bid). Subjects who are <60kg and initiated on 30mg bid, who gain weight to more than 70kg, may switch during the study to 40mg bid. Per the investigator decision, if a trial participant's is at high risk for developing toxicity from D4T (per risk profile such as but not exclusionary such BMI >28 and women of African descent) this drug may switch to AZT at or after 6 months. No dose modification for Lamivudine and lopinavir/ritonavir is considered.
Dosage reductions in the table below are permitted as specified in the toxicity management section below.

B. DOSAGE REDUCTION (TABLE 8)

DRUG	INITIAL DOSE	DAILY DOSE	REDUCED DOSE	DAILY REDUCED DOSE
3TC	150 mg BID	300 mg	None	None
3TC	300 mg QD	300 mg	None	None
d4T	40 mg BID	80 mg	20 mg BID	40 mg
d4T[2]	30 mg BID	60 mg	15 mg BID	30 mg
AZT	300mg BID	600mg	200mg BID	400mg

DRUG	INITIAL DOSE	DAILY DOSE	REDUCED DOSE	DAILY REDUCED DOSE
LPV/r	133.3 mg lopinavir/33.3 mg ritonavir<br>3 capsules BID	800 mg/200 mg	None	None

[1]For subjects weighing ≥60 kg.

[2]For subjects weighing <60 kg.

Dose reduction for zidovudine may be considered if subjects develop Grade 2 or more haematological complications as a result of bone marrow suppression with zidovudine. Such a dose reduction will be at the discretion of the site investigator. As zidovudine is a second line drug, dose reduction will be encouraged before subjects are removed from the study.

C. Toxicity Management – Grading

Grade 1 or 2
Subjects who develop a Grade 1 or 2 adverse event or toxicity may continue study drugs without alteration of the dosage except as stated in the following sections. Subjects experiencing Grade 1 or 2 toxicities will be managed at the discretion of the study clinicians.

Grade 3
If there is compelling evidence that the adverse event has NOT been caused by the study drug(s), dosing may continue. Except as stated in the following sections, subjects who develop a Grade 3 adverse event or toxicity thought secondary to study medications or of unknown etiology will have all their antiretroviral study drugs withheld, and at the investigator's discretion substitutions allowed after reaching Grade 2. Investigators are encouraged to discuss toxicity management with the Protocol Chair and other Investigators. The subject should be reevaluated weekly until the adverse event returns to Grade ≤ 2, at which time the study drugs may be reintroduced at the discretion of the investigator or according to standard practice

Grade 4
Subjects who develop a symptomatic Grade 4 adverse event or toxicity not specifically addressed below will have all study drug(s) withheld until resolution of the adverse event to a Grade ≤ 2. Alternative study-provided medications should be considered.

Subjects with Grade 4 asymptomatic laboratory abnormalities, not specifically addressed below, may continue study drug therapy if the study clinician has compelling evidence that the toxicity is NOT related to the study drug(s).

D. Dose interruption:

Toxicities will be graded according to the DAIDS Grading Scale 1-4 scale (Appendix B). If a toxicity is encountered, the dose administration to the subject may require interruption. Subjects who develop a study drug-related Grade 3 or 4 adverse event or laboratory abnormality (with the exception of hyperglycemia, hypertriglyceridemia, hypercholesterolemia, or Grade 3 AST/ALT elevations in hepatitis co-infected individuals) –should interrupt all study medications. Should an interruption of treatment not require permanent discontinuation or treatment switching, then reintroduction of treatment should be at the same dose. If the subject's adverse events or laboratory abnormalities have not resolved to within one grade (not to exceed Grade 2) of his or her baseline level within eight weeks of study drug interruption, the subject should be discontinued from the study. The subject will be informed that the side effects listed below can be side-effects of the different drug classes. Any permanent discontinuation is considered an end-point in the study, although subjects will be followed as long as they are willing to participate.

E. Specific Management of Laboratory Abnormalities and Clinical Syndromes

Lipase Elevations and Pancreatitis

Pancreatitis will be reported as a clinical finding (i.e., symptomatic pancreatitis). The enzyme abnormality that will be used for making diagnoses is the lipase level. When obtained, lipase determinations will be recorded in the CRF.

Lipase will be obtained for subjects if development of clinical symptoms suggest pancreatitis. If a baseline measurement is needed, it will be performed from stored samples.

For symptomatic (gastrointestinal symptoms, particularly abdominal pain) subjects with elevations in lipase:

Grade <1: Search for other causes of symptoms. If none are found and symptoms persist, repeat lipase within 2 weeks.

Grade 1 or 2: Follow subjects and repeat lipase as soon as possible; within 2 weeks is optimal. If lipase remains elevated, but is Grade <3 and symptoms persist, then subjects should either be considered to have clinical pancreatitis or continue to be followed at frequent intervals, depending on the best available clinical judgment. CT scan of the abdomen, if available, may be helpful in determining whether clinical pancreatitis is present.

Grade ≥3: Exclude other possible diagnoses (e.g., renal insufficiency causing false elevations in lipase). If none is found, diagnose as clinical pancreatitis.

For a diagnosis of pancreatitis (clinical), all study medications should be held.

With complete resolution of the episode in a setting in which other concomitant illness might have reasonably contributed to the development of pancreatitis, rechallenge with study medications may be performed.

Upon rechallenge, lipase determinations should be performed monthly. Any elevation of lipase of Grade $\geq$2 or any recurrence of symptoms during this period will lead to a re-evaluation and permanent discontinuation of the suspected study drugs(s).

CK Elevation

CK measures will not be performed routinely as part of the protocol. CK will be measured subsequently only if subjects develop clinical symptoms consistent with a diagnosis of myositis. If a baseline measurement is needed, it will be performed from stored samples.

For persistent CK elevations >3000 mg/dL (about 20 x ULN) in symptomatic subjects, CK should be redrawn after subjects abstain from exercise for 24 hours before treatment modifications are made.

AST and ALT Elevation

Nearly all the antiretrovirals can cause alterations in liver functions tests. Further, concomitant illness may also alter these laboratory parameters. Therefore, changes in AST or ALT should be evaluated within the clinical context of the abnormalities.

General Considerations: For asymptomatic elevation in AST or ALT < 5-10 × ULN (Grade ≤ 3), medications may be continued at the discretion of the site investigator. Careful assessments should be done to rule out the use of alcohol, non-study medication-related drug toxicity, the lactic acidosis syndrome, and viral hepatitis as the cause of the transaminase elevation. If the AST/ALT elevation is considered most likely to be due to concomitant illness or medication, standard management, including discontinuation of the likely causative agent, should be undertaken.

For asymptomatic elevation 5-10 × ULN and >10 x ULN (Grade 3and 4) believed secondary to study medications, all agents should be withheld until levels are Grade ≤ 2. Recurrence of Symptomatic Grade 3 or any grade 4 elevation of liver enzymes will require permanent discontinuation of the medications.

Hepatitis B or C coinfection: at study entry, hepatitis B surface antigen (HBsAg), will be obtained. Results will be made available to study clinicians. Hepatitis B and/or C seropositivity is an exclusion criterion for this study. Any subjected noted to be Hepatitis B positive during the study will be required to discontinue.

Peripheral Neuropathy:

Subjects should be monitored for the development of peripheral neuropathy, which is usually characterized by numbness, tingling, burning or pain in the feet or hands.

Any subject who develops a treatment emergent Grade 1 or 2 peripheral sensory neuropathy must be carefully monitored with monthly visits initially and must be

educated about the condition and counseled to return to the clinic if the neuropathy is progressing.

Any subject who develops a Grade 3 or 4 peripheral neuropathy should discontinue their current regimen (all drugs) until resolution to grade 2 or less. Reintroduction of treatment should be considered with substitution of AZT for D4T.

Anemia/Neutropenia

Where the primary regimen has been altered to substitute AZT for D4T, bone marrow toxicity due to AZT may occur. For Grade 3/4 anemia, and grade 4 neutropenia, and thrombocytopenia believed secondary to AZT, study treatment should be discontinued until resolution of the adverse event to grade < grade 2. Study treatment should be held until the toxicity event returns to Grade ≤ 2, at which time treatment may be resumed with AZT at level-one dose reduction of AZT (200 mg BID). If the same Grade 3/4 adverse event recurs on a reduced dose study treatment should be discontinued. The investigator in conjunction with the study team may choose to continue AZT at a reduced dose in the setting of Grade 3 anemia if the risks of discontinuing the AZT outweigh the benefits and a switch to an alternative antiretroviral therapy regimen.

Subjects with Grade 4 anemia or neutropenia attributed to AZT will have treatment interrupted until the adverse event has returned to Grade ≤ 2. Once the anemia or neutropenia has returned to Grade ≤3, all ART should be restarted, AZT administered at a reduced dose of 200 mg BID.

Nausea (with or without vomiting)

Although common, nausea following initiation of therapy with antiretroviral medications usually subsides or resolves during the first few weeks of treatment.

Diarrhea

Diarrhea is a common side effect of infection and medication toxicity. If no infectious cause of diarrhea is found and onset is temporally related to new medication, symptomatic management with anti-diarrheal agents is appropriate.

Lactic Acidosis

The relevance of asymptomatic lactic acid elevations is unclear, and lactates are not part of the routine safety evaluations for this study. Routine lactate monitoring is not currently recommended. No baseline lactic acid levels will be obtained.

A sometimes-fatal syndrome of lactic acidosis, often associated with evidence of hepatic steatosis, is a recognized but rare complication of NRTI therapy. This syndrome is felt to be secondary to mitochondrial toxicity induced by the inhibitory effect of NRTIs on DNA polymerase gamma, a key enzyme needed for mitochondrial DNA synthesis. Current knowledge regarding this syndrome is incomplete. Obesity, gender, and prolonged NRTI exposure may be risk factors. Symptoms of lactic acidosis frequently

involve nonspecific symptoms such as fatigue, weakness, and fever, but in the majority of cases also involve symptoms suggestive of hepatic dysfunction such as nausea, vomiting, abdominal or epigastric discomfort, abdominal distension, hepatomegaly, and new onset elevated liver enzymes. A high index of suspicion may be required to diagnose this condition. Alternatively, it is possible that unwarranted concern may be raised by over interpretation of lactic acid levels. NRTI toxicity is only one cause of lactic acidosis. Lactic acid elevations are also seen in the context of diabetes mellitus, uremia, liver disease, infections, malignancies, alkaloses, and drug and toxin ingestion of such substances as ethanol, methanol, ethylene glycol, and salicylates.

The following case definition of lactic acidosis, defined differently for symptomatic versus asymptomatic individuals, will be used:

Symptomatic Hyperlactatemia

New, otherwise unexplained, and persistent (≥2 weeks) occurrence of one or more of the following symptoms:

Nausea and vomiting, abdominal pain or gastric discomfort, abdominal distention

Unexplained fatigue and/or Dyspnea

Plus an elevated lactate measurement and/or increased LFTs.

Table 9. Proposed Strategy for Interpreting Lactate Levels

Lactate	Symptoms	Action
<2mmol/L	No	No intervention
	Yes	Investigate other causes
2-5mmol/L	No	Observe
	Yes	Exclude other causes<br>Consider discontinuation.
>5-10mmol/L	Both Yes or No	Discontinue NRTI<br>Exclude other causes.

VIII. Criteria for Subject Discontinuation from Randomization with retention of follow-up

- Any two viral loads above 5000c/ml on treatment (in the intermittent treatment arm "on treatment" is defined as more than 12 weeks of treatment).
- CD4+ T cell count number 15% below the entry count OR below 350 cells/mm^3 after 20 weeks of therapy
- Drug related toxicity defined as investigator directed treatment permanent discontinuation or investigator directed unscheduled interruption of more than 8 weeks in association with Grade 3 or above toxicity. (See Toxicity Management). Subject requested treatment discontinuation due to toxicity even if this does not meet objective criteria is considered a reason for discontinuation related to toxicity.
- Pregnancy or breast-feeding in the intermittent therapy arm.
- Clinical reasons believed life threatening by the physician, even if not addressed in the toxicity management of the protocol.
- Subject reaches a defined study endpoint, if applicable.

IX. Criteria for Permanent Subject Discontinuation from Study

- Requirement of a prohibited concomitant medications or requirement for exclusionary criteria #4.
- Request by the subject to withdraw consent and terminate monitoring.

X. Statistical Considerations

A. Randomization Procedure

After the introductory period of antiretroviral therapy (-12 to 0 weeks), and with the confirmation of laboratory evaluation of HIV PCR Viral Load <50 copies/ml, in the absence of therapy related toxicity, the patients will be considered in the immune reconstitution period (0 to +24 weeks). During the immune reconstitution period, patients need to complete 3 doses of rabies vaccination. Patients, who retain an undetectable viral load over a period of 24 weeks and have completed the schedule of rabies vaccination, will be eligible for randomization. Subjects will be randomly allocated 1:1 to either the intermittent or continued therapy using permuted block randomization with random block sizes of 6 to 9, to maintain an equal number of subjects in each group.

The randomization list will be generated and kept by the Protocol Biostatistician. She will be the only person in the study who has access to the randomization code. A randomization number for each new patient will be assigned via e-mail to the Study Coordinator by the Biostatistician or her appointed assistant. Given the nature of the design, the study cannot be blinded. Therefore, the varying block sizes, having only the Biostatistician maintain the randomization list, and having the Study Coordinator not discuss the current makeup of the cohort with the pharmacist, providers, or potential study subjects will minimize the possibility of bias in the referrals received. The hardcopy of the randomization will be maintained in the site file.

B. Sample Size and Power Considerations

The primary aim of the study is to determine if the STI strategy results in subjects maintaining CD4 recovery, as would be expected in the continuous therapy arm. When the sample size in each group is 26, a two group 0.025 one-sided t-test will have greater than 85% power to reject the null hypothesis that the test and standard are not equivalent (the difference in means is 10.0 or farther from zero in the same direction) in favor of the alternative hypothesis that the means of the two groups are equivalent. Sample size calculations were done using the statistical software package nQuery (Statistical Solutions, Cork, Ireland.) Based on the Philadelphia cohort of STI subjects, individuals in the continuous therapy and STI arms would be expected to maintain CD4 recovery 98% and 96% of the time-points assessed, respectively. A standard deviation of 7.07 was derived from data on 42 patients (21 on continuous therapy and 21 on structured treatment interruptions) measured over 40 weeks with a minimum of 6 time points per subject.

This sample size will also provide greater than 90% power to determine if the STI strategy results in subjects maintaining the reconstituted immune responsiveness to a neoantigen (rabies), as would be expected in the continuous therapy arm (alpha=0.025, expected difference=0, limit difference=5%, standard deviation=5.01.) An important series of secondary hypotheses of this study is that subjects in the experimental arm, given the decreased drug exposure, will have lesser degrees of treatment associated toxicities (triglyceride levels, HDL and LDL cholesterol levels, waist-to-hip ratios, body mass index, insulin resistance, and bone mineral content) while retaining immune reconstitution changes (increase in CD4 count, retained recall responses) and the ability of the regimen to re-suppress when reinitiated. The ability to detect a difference or lack of difference in these parameters depends upon the sample size, the desired power (80%), the type I error rate (two-sided alpha=0.05) and the standard deviation for each of the variables. A useful method for reporting these detectable differences is by effect size. Effect size is a unitless number that allows comparison between unrelated variables and is defined as the detectable difference divided by the standard deviation; in other words, the effect size reflects the magnitude of detectable effect reported in relation to the standard deviations. With our minimum sample size of 26 per arm, we will be able to determine an effect size for all of the variables of approximately 0.8, which is in the moderate range. This unitless number expresses the magnitude of the detectable difference in terms of the standard deviation of the variable of interest. Therefore, for each individual variable, multiplying the standard deviation by 0.8 will provide the absolute magnitude of the detectable difference. Variables with more variability will be more difficult to demonstrate the effect in given the fixed sample size, and vice versa. For example, we used data generated on 107 South African men and women to determine the standard deviation of the lipids we will be assessing. The table below demonstrates the differences we will be able to detect between the experimental and control groups.

Variable Detectable Difference
Total cholesterol 32 mg/dl
LDL cholesterol 27 mg/dl
HDL cholesterol 17 mg/dl
Triglycerides 48 mg/dl

These differences are in the range found by Hatano et al. in a study of treatment interruptions designed to impact on metabolic parameters (Hatano et al. AIDS 2000). While smaller differences may still be relevant, the primary aim of this study is not simply decreased toxicity, but rather decreased drug exposure overall. Therefore, if we find smaller, non-statistically significant, but potentially clinically significant differences, if this study is found to be immunologically safe, larger studies to determine the potential toxicity benefits more precisely would be warranted. In addition to these lipid changes, we will be assessing for changes in body shape (e.g., waist to hip ratio) and bone mineral density as well, however, these analyses will be more exploratory in nature.

C. Impact of Intermittent Therapy On Outcomes: Analysis Overview

The primary end-point will determine whether this strategy is safe in preserving the CD4 recovery that is the primary benefit of HAART. CD4 recovery will be defined as the proportion of measurements at which an individual's CD4 count is greater than 350. Over time, the percentage of times an individual exhibits this response is a continuous variable between 0% and 100% and will heretofore be referred to as "percent of time responding." Therefore, each individual will contribute a percent response to the final analysis and the mean "percent of time responding" for each arm will be compared. Our primary interest is in the effectiveness of this approach (i.e., whether the strategy works), not the efficacy (whether the biological hypothesis underlying the strategy is correct). Therefore, an intent-to-treat analysis will be primary and therefore will only be relevant if the subjects are able to tolerate the treatment interruption. Individuals who drop out due to treatment-related toxicity (Grade 3 or above) or lack of viral suppression on ART, their remaining time on study will be counted as time in which the immune reconstitution was lost and each intended measurement will be scored as if they had a lack of response. We will address the question of efficacy in several secondary analyses. We will perform instrumental variable analyses using randomized treatment assignment as an instrumental variable to obtain estimates of efficacy while adjusting for treatment non-adherence. Additionally, we will perform a sensitivity analysis for the impact of drop-outs by performing all analyses both with and without drop-outs included, and also just on drop-outs alone. Additionally, to extend the sensitivity analyses for the effect of dropout, we will perform analyses that assume informative dropouts, such as shared parameter models [38].

In addition to analyses of the primary endpoint, percent of time responding, this section describes statistical methods for analyzing safety/toxicity, immune reconstitution, and resistance. In all cases, summary statistics including means, medians, standard deviations and ranges will be calculated. In addition, both cross-sectional and longitudinal analyses will be performed. The latter will allow us to capture information on trends over time while accounting for correlations in the data (arising from multiple measures on each individual over time.) Analyses will be performed using Stata 8.0 (Stata Corporation, College Station, TX) and Splus 6.0 for Windows (Insightful Corporation.)

D. Primary End Point Analysis: Comparison of the Relative Maintenance of Immune Reconstitution via Maintained CD4 Recovery

Description of the study data per group.

The baseline characteristics of the experimental and the control groups at both initiation of antiretroviral therapy ("run-in" period) and the start of the experimental phase (STI trial) will be

compared. Continuous variables such as age, viral load at enrollment, and entry CD4 counts will be summarized by mean, standard deviation, 95% confidence intervals, median, and range. Categorical variables will be summarized by frequencies.

Primary Analysis-Intent to Treat. The primary analysis will compare the average amount of occurrences of CD4 count remaining above 350 between the groups. For each individual, the percent of measurements with response will be defined as the primary outcome. Summary statistics, including the mean and median percent of times with CD4 counts above 350 will be calculated for each group. The distribution of the percent of time responding will be summarized for each group using histograms. Appropriate transformations (e.g. log, square root) will be used in the absence of normality. A one-sided 97.5% confidence interval for the difference between the 2 arms in the mean percentage of measurements with CD4 above 350 will be constructed. If this confidence interval covers 10% then we cannot conclude non-inferiority. If the right hand limit of the confidence interval is less than 10% than we will conclude that the experimental and control arms are equivalent. In order to make less stringent missing-data assumptions and to evaluate trends over time, a non-linear mixed effects model with random person specific intercept and slope terms will be fit to the data. The response variable in this model will be an indicator for positive response criteria. Individuals who drop out due to treatment-related toxicity (Grade 3 or above), therapy failure (lack of suppression on therapy, or study-defined changes in CD4 count) will be assigned a score of "0" or loss of immune reconstitution for the remaining time-points. Individuals who become pregnant and discontinue treatment will lead to censorship of data past date of discontinuation.

Although randomization typically eliminates the need for controlling for potential baseline confounders, if imbalance is found between the groups, controlling for confounding would be an important step in subsequent analyses. Potential confounders will be controlled for in multivariable linear regression models for the primary outcome and in the mixed effects models for the longitudinal analysis. The potential confounding variables will be viral load and CD4 count prior to therapy, change in CD4 count prior to the randomization step, and pregnancy. Each of these variables is plausibly related to percent of time remaining above baseline. Throughout the primary and secondary analysis plan all p-values will be two-sided; the test for the primary outcome will be one-sided based on the non-inferiority hypothesis.

Secondary analyses - Accounting for poor adherence. In further secondary analyses of this primary endpoint, only individuals who have been demonstrated to adhere to ≥95% of all of their prescribed drugs over the course of the study will be included. This cutoff was chosen based on prior data of adherence differences between subjects on protease inhibitors who achieved undetectable viral loads as compared with those who fail to fully suppress [12]. Identical analyses will be performed on this subgroup as described above for the full cohort. To supplement this analysis based on adherence-defined sub-groups and the intent-to-treat analysis, which will provide an estimate of effectiveness of the cyclic intermittent treatment under non-adherence, we will perform a test of efficacy using an instrumental variable technique recently developed for the Cox model [46]. This statistical modeling approach will provide an estimate of the effect of adhering to the prescribed STI treatment.

E. Secondary End Point Analysis: Comparison of the Relative Maintenance of Immune Reconstitution via Response to Neoantigen (Secondary Aims 2 and 6, page 11)

In addition to whether the CD4 count is maintained above 350, the response to a neoantigen will also be tested as a measure of immune recovery. As specified in methods, LPA and neutralizing titers will be measured for each individual and defined to have a response yes/no based on whether they demonstrate an SI >3 against the vaccine antigen and/or maintain a serum titer of neutralizing antibody of 0.5IU. For each individual, the percent of measurements with response will be defined as the primary outcome. Descriptive analysis, testing of non-inferiority, modeling outcomes over time and accounting for adherence and potential confounding will proceed as described above for the primary outcome. Although primary analysis will combine humoral and cellular responses, secondary analysis will be similarly performed for each response individually, as in the case of rabies the humoral response is associated with protection. Individuals who drop out due to treatment-related toxicity (Grade 3 or above) or therapy failure will be assigned a score of "0" or loss of immune reconstitution for the remaining time-points. Following CSR review of our protocol, concern was expressed as to the persistence of the rabies response leading to the introduction of an evaluation of a booster response as a highly complementary secondary aim. Evaluation of the response to a booster stimulation to rabies antigen as a secondary analysis will be approached in the manner as described for the primary analysis of rabies responses yet with frequency of responders in each group evaluated at a single time-point and analyzing for differences in magnitude between groups.

F. Secondary Aim Analysis: Safety/Toxicity Comparisons between groups (Secondary Aims 1, 3 and 9, page 10-11)

Subjects stopping the study by safety protocol criteria in *Section VI. - M. Post-Randomization: Experimental Arm: Safety parameters during ART interruption* and *Section VIII. Study Treatment toxicity Management*" will be used to address formal statistical testing of the proportion of subjects experiencing any adverse events between arms using exact methods [47] for dichotomous variables (e.g., development of pancreatitis, decrease of CD4 count to below 350 cells/mm^3) and two-sample t-tests of the Wilcoxon rank sum test, as appropriate, for continuous variables (elevations in BMI). For the dichotomous variables, we will also test the proportion of subjects experiencing the individual adverse events as well as a combined endpoint of "any" adverse events between the groups using exact methods [47]. In further safety analyses, we will compare the rate of drug AEs as well as the rate of the emergence of resistant virus between the groups, also using exact methods. For the purposes of testing whether the experimental treatment results in equivalent or better outcomes overall, adverse events will be defined as any clinical or laboratory abnormality that arises whether or not it is attributed to the treatment interruption, the medications or both. Since the development of adverse drug effects may be delayed in the experimental arm, but adverse effects of the treatment interruptions may be increased in that arm as well, and because one of the major goals of this project to it increase the duration of both disease and toxicity free survival, it will be important to compare the experimental and control groups with respect to time to adverse events. We will therefore perform standard survival

analyses, including Kaplan-Meier plots and log rank tests, to compare the groups with respect to time to the development of treatment or HIV-associated toxicities [48]. We will compare the actual values as well as the changes in several drug toxicity variables including triglycerides, LDL and HDL cholesterols, waist-to-hip ratio, insulin, proinsulin, C-reactive peptides and anthropometric data such as body mass index (BMI) at the end of the comparative observation period.
In addition, we will assess the effect of treatment interruption on cardiovascular risk factors by comparing the values and change from baseline of proinflammatory and endothelial activation markers (vWF, Apo-B, VCAM-1, Thrombomodulin, tPA, TNF-α, ICAM-1 and IL-6), and assessing the number of circulating endothelial precursor cells, between interrupting and continuous therapy group, using 2-sample t-tests or Wilcoxon rank sum tests as appropriate. Additionally, within group comparisons of suppressed and viremic time points (before and after treatment interruption) based on paired t-tests or Wilcoxon signed rank tests, and Spearman's correlation to viral load, will allow us to determine the effect of viremia on vascular inflammation (please refer to attached schedule of events for time point sampling information).
For example, for continuous variables such as BMI, we will compare the levels of these variables at the end of observation as well the magnitude of change in these variables within and between the groups over time. As before, if the variables are normally distributed or can be transformed to normal distributions, they will be compared using Student's t-test. Otherwise, the Wilcoxon Rank Sum test will be used. Fat redistribution will be analysed using MRI-and DEXA-scans at the beginning and MRI at the end of the study in conjunction with detailed anthropometric measurements taken over the course of the study. Longitudinal analysis of metabolic and anthropometric variables will allow us to assess the effect of treatment duration on these parameters and whether treatment interruption can attenuate the toxic effects of HAART. In addition, it will also be possible to correlate any changes in the metabolic variables with changes in body fat re-distribution. This will allow us to determine whether hyperlipidemia, and insulinaemia are dependent on lipodystrophy or are direct effects of HAART.

G. Secondary Aim Analysis: Immune Reconstitution (Secondary Aim 2, page 11)

We will also compare the change in CD4 count since baseline at each analysis between the groups using either Student's t test or Wilcoxon rank sum test, depending on the distribution of the data. Our sample size will provide us with 80% power to detect a difference from 0.0 to 0.02 in the proportion of people whose CD4 count drops below their baseline level. Activation markers will be expressed as the percent of positive events of the lymphoid population and/or mean fluorescence intensity (MFI). These markers will be summarized with means, medians, standard deviations and percentile ranges for both groups. We will graph changes in these measures over time for visual assessment. Data obtained from both group of patients will be amenable to both cross-sectional and longitudinal analyses. The cross-sectional analysis will compare the percent positive populations between the groups at the final time point using Student's T-tests or the Wilcoxon rank sum test, depending on the distribution of the data [47]. The longitudinal analysis will address the potential dose-response nature of the data. For this analysis, since the amount of percent positive cells might be different while on HAART versus off HAART, we will perform two sets of analyses in pre-randomized groups and in the experimental group. The first analysis will include time points off and on HAART (i.e., pre-randomization) to determine if, over time, the amount of cells in each subset measured is changing in relation to immune reconstitution and decreased viral replication. The second analysis will include only those time points

on/off HAART (i.e., baselines of each STIs). Since a clinically relevant change in markers other than CD4 has not been established, we will simply assess the correlation between levels of percent positive T-cell subsets and viral suppression following each reinitiation of therapy. We will use either the Pearson correlation coefficient or the Spearman rank-correlation coefficient, depending on the distribution of the immune parameters [47]. All analyses will be performed using Stata 8.0 (Stata Corp., College Station, TX) on a Pentium-based PC. All p-values will be two-sided.

The level of recall responses against CMV, Candida and TB antigens (PPD) and HIV-1 will be summarized and compared between baseline, randomization and study end-point. Subsequent follow-up between groups will also be compared. First, we will describe the data with means, standard deviation, 95% confidence intervals, median, and range. In the absence of normality, transformations (e.g., log or square root for skewed data) to achieve a normal distribution will be employed. We will perform two sets of analyses on these data, one cross-sectional and the other longitudinal. We will compare the immune response between the groups in a cross-sectional fashion using Student's T-test or the Wilcoxon Rank Sum test [47], as applicable, to determine if the strategy decreases the frequencies of lymphoproliferative responses (indicators for SI>3 as well as delta cpm) or the frequency of CD4 T cells and CD8 T-cells that proliferate or produce IFN-γ/Ki67 in response to recall antigen stimulation. Each of these analyses will be performed for each of the stimuli tested. In the first set of cross-sectional analyses, the responses at the final time point will be compared between the groups. We will also compare the change in responses for each individual between their initial measurement and final measurement, using paired T-tests or signed-rank test, as applicable. Of course, these cross-sectional analyses are limited by the fact that they ignore the measurements between the initial and final time points. We will address this limitation by doing longitudinal analyses. These will also compare the responses between the groups, but will allow for testing for the presence of trends in the changes over time. These tests have more statistical power to detect a difference in response than the cross-sectional analyses [47] and would lend further biological credibility if showing no difference between arms as hypothesized for non-HIV antigens (as shown in preliminary data, HIV recall responses can temporally increase during therapy interruption episodes). We will use random intercept/random slope mixed models [50] to characterize the change in each stimulus over time while accounting for the correlated nature of the data (i.e., multiple observations over time per individual). Clinical parameters will be used as covariates in these models as appropriate. Transformations will be performed to ensure normality assumptions of the mixed effects models are met. Nonparametric mixed modeling techniques will be employed if necessary [51]. The results of these analyses will be a comparison of the trend in immune response over time between the groups.

H. Secondary Aim Analysis: Resistance Mutations (Secondary Aim 3, page 11)

Data will first be assembled, edited and analyzed using the supplied ViroSeq software. Sequence data from all six primers are combined into a single project and a consensus sequence of PR (aa 1-99) and RT (aa 1-335) is created. A navigation bar displays the entire sequence with a cursor indicating the position under edit. The editing window displays the codon position, amino acid translation, reference nucleotide sequences from HIV-1 pNL4-3 and the sample nucleotide sequence as well as the electropherograms for the forward and reverse primers. Reporting of results in simple FASTA sequence as well as an Antiretroviral Drug Resistance report will be generated. This report converts the sequence data into a

table of genotypic mutations with known and novel variants as well as insertions. Mutations are classified according to their association with decreased drug susceptibility as defined in the interpretation algorithm.

In addition sequence data from the electropherograms will be analyzed using Sequencher software. FASTA formatted sequences will be aligned and analyzed using Clustal X and sent to HIV-SEQ, the Stanford HIV RT and Protease Sequence Database (**http://hivdb.stanford.edu**) which produces a table of resistance-associated mutations. This information will be compared to the data obtained from the ViroSeq report. These tables will serve primarily as descriptive tools. Based on the results of these analyses, treatment failures will be classified according to whether or not they resulted from resistance mutations. In addition all resistance mutations will be compared whether or not these were associated with treatment failure. The proportions of failures due to resistance will be compared between the two arms using a chi-squared test of equivalence between two proportions.

Sequences will also be used to subtype viruses and to examine phylogenetic relationships. For phylogenetic tree construction, aligned sequences from Clustal X will be used to generate a distance file and subjected to a bootstrap analysis using SEQBOOT. The original distance matrix will then be used as input into PHYLIP and TREEVIEW to generate a final phylogenetic tree. Sequences described in this study will be submitted to GenBank.

XI. Data collection and data management

Case report forms (CRFs) will be provided for each subject. Subjects must not be identified by name on any CRFs. Subjects will be identified by the patient identification number (PID) and study identification number (SID) provided by the Protocol Biostatistician upon randomization.

A. Clinical Data Collection: Biological Samples (I. Sanne/W. Stevens/L. Montaner)

Clinical data regarding viral load, CD4 cell count, CBC, metabolic tests bloods and PBMC/serum collection is summarized by visit in both control and experimental arms in blood draw tables 6 and 7. Time-points listed below are derived from the above referred tables. In summary, a minimum of 5 mls (CD4, CBC and flow cytometric analysis) and a maximum of 90 ml (15 mls for CD4, CBC, viral load and liver/kidney tests; 12 mls for insulin and fat metabolism tests, and 64 mls for PBMC isolation) of blood are to be collected at any one time-point with a maximum of 184 mls of blood to be collected within a 3 month interval (12 weeks). All sample analysis will be conducted in a local SANAS certified laboratory. SANAS is the South African National Accreditation body ensuring GLP standards are maintained.

CD4 cell count, dual platform method: Fifty microlitres of whole blood (K_3 EDTA tube) is added to CD8 (FITC)/CD4(PE)/CD3(ECD) Coulter Cytostat trichrome antibody reagent; vortexed and incubated at room temperature in the dark for 15 minutes. Data is acquired on the EPICS XL-MCL flow cytometer (Beckman Coulter, Miami, FL). At least 2000 lymphoid events are collected in an automatic 'lymphoid' gate. All results are reported as percentages and absolute cell counts according to international guidelines. External Quality Assessment (EQA) of these assays will be maintained using the UKNEQAS scheme.

Viral Loads (derived from same 5 ml K_3 EDTA tube used for CD4 cell count): A commercially available assay, the Roche Amplicor HIV-1 monitor (Roche Molecular Systems, California) (Standard or Ultrasensitive) will be used to quantitate HIV RNA in the plasma. The sample will be prepared and analysed using the following Roche instrumentation: the Cobas Ampliprep and Cobas Amplicor analysers. Since genetic variability of HIV has been shown to affect the quantitation of HIV RNA by different techniques, the version 1.5 (v1.5) of the assay will be used since this has demonstrated superior performance for HIV isolates other than subtype B. The laboratory conducting these assays will be certified using the DAIDS VQA Program.

Liver/Kidney Function tests (10 ml red top): the following parameters will be measured: liver/kidney function tests (urea, creatinine, total and direct bilirubin, ALT, AST, ALP, GGT). Amylase, lipase and blood gases will only be tested if needed. These will be performed using the Roche Cobas Integra 400 for Biochemistry according to the following methods on serum samples: ISE (electrolytes), Urease and Dehydrogenase (Urea), Enzymatic colorimetric (Creatinine), Turbidometry (Lipase), Ethylidine-G7-PNP (Amylase), IFCC without pyridoxal phosphate activation (ALT). Full blood count analysis will be conducted on the Coulter series of analysers using impedance technology (FBC). All safety tests will be monitored using the CAP EQA schemes for Chemistry and Haematology.

Female subjects will also be tested for **pregnancy** by the rapid urine test. Clinical data will be analyzed in real time by the clinical investigators. Summary statistics will be calculated as described in analysis section. Clinical data will also be used as time-varying covariates in longitudinal analyses.

Biological sample collection for laboratory analysis of immune and viral outcomes will be derived from a total of 8x 8ml draw venous peripheral blood samples collected in CPT tubes (independent of blood draws for viral load, CBC and CD4 cell count) for an anticipated yield of 70-80x10^6 PBMC in addition to plasma. Citrate-CPT tubes contain PBMC isolation reagents that allow for faster isolation requiring less effort than the standard Ficoll-based procedure. 1 ml of the blood volume collected for viral load and CD4 cell count will be used for flow cytometry as the same laboratory will have excess blood for these. PBMC isolated from the 8 CPT tubes (~64 mls blood, >75 x 10^6 cells) will be cryopreserved in three 25x10^6 aliquots which will be used at designated times to measure rabies lymphoproliferative response (3.6 million), lymphoproliferative responses to recall antigens (9.6 million PBMC), and quantization of T cell expressing cytokine and Ki67 in response to HIV ad CMV antigens (7.5 million). Committed use of PBMC totals 20.7 million, but as recovery from cryopreserved PBMC is around 50-60%, a total of 2 aliquots of cryopreserved PBMC (50 x 10^6 cells) will be required. The remainder aliquot (25 x 10^6 cells) will be stored in Johannesburg for follow-up analysis and not included in shipments to The Wistar Institute in case of an unanticipated loss of samples during shipment or for other causes (note: all shipments will be escorted by a research team member to minimize custom problems associated with unescorted shipments, see administrative travel and shipment budgets). Serum derived from citrate-CPT tubes will be stored as four 2ml aliquots for viral genotype studies.

Soluble factors associated with cardiovascular risk: An aliquot of the cryopreserved serum (see above) will be shipped to the Wistar Institute for analysis. Using commercial ELISA kits, and following the manufacturer recommended protocols, we will evaluate the following factors:

Factor	Manufacturer	Distributor
Von Willebrand	Affinity Biologicals Inc.	DiaPharma Group, Inc.<br>Westchester, OH 45069

Apo-Lipoprotein B	Exocell Inc.<br>Philadelphia, PA 19104	
VCAM-1	BioSource International	Invitrogen Corporation<br>Carlsbad, CA 92008
Thrombomodulin	American Diagnostica<br>Stamford, CT 06902	
Tissue Plasminogen Activator (tPA)	Affinity Biologicals Inc.	DiaPharma Group, Inc.<br>Westchester, OH 45069
TNF-alpha	BioSource International	Invitrogen Corporation<br>Carlsbad, CA 92008
ICAM-1	BioSource International	Invitrogen Corporation<br>Carlsbad, CA 92008
IL-6	Peninsula Laboratories Inc.	Bachem Bioscience Inc.<br>King of Prussia, PA 19406

A Research Assistant with extensive experience with ELISA-based assessment of serum proteins will be responsible for these assessments. Serum from healthy volunteers, available through the Wistar Institute blood donor program, will be used as negative control, as well as for test optimization and QC.

Anticipated problems as encountered in interrupting cohorts studied to date consisted in lower PBMC yields in patients with viral loads > 100,000 copies/ml. In the event that PBMC yield is lower than expected, analysis will be prioritized in the following manner: flow cytometry > rabies recall responses > recall responses.

B. Clinical Data Collection: Measurement of Metabolic Variables, Adipose Tissue Distribution and Bone Mineral Density, and (N.J. Crowther,)

Measurement of skinfold thickness: Skinfold thickness will be measured with calipers at 4 sites: triceps, biceps, subscapular,and suprailiac. Sites will be identified using a tape measure, as follows:

(a) Triceps: a fold on the posterior midline of the upper arm over the triceps muscle, halfway between the acromion process and olecranon process.
(b) Biceps: a fold on the anterior surface of the biceps midway between the anterior axillary? fold and the antecubital fossa.
(c) Subscapular: a fold on the diagonal line coming from the vertebral border to between 1 and 2 cm from the inferior angle of the scapulae.
(d) Suprailiac: a diagonal fold above the crest of the ilium at the position where an imaginary line would come down from the anterior axilliary line.

Measurements will be taken on dry skin on the right side of the body. A minimum of two measurements will be taken at each site and if repeated measures vary by more than 1 mm the measurement will be repeated.

Measurement of plasma glucose levels (2 ml grey top): Glucose levels will be measured using an automated enzymatic colorimetric assay from Roche (Mannheim, Germany). This assay is run on the Roche Integrer autoanalyser. Measurement of serum insulin, proinsulin and C-peptide levels (10 ml red top): The insulin assay will be performed on serum samples using a chemiluminescent immunometri assay kit from DPC. The assays will be performed on the Immulite autoanalyser (DPC). Proinsulin will be measured using a manual enzyme immunoassay supplied by Dako (Ely, England). This assay is specific for intact proinsulin and does not cross react with human insulin. C-peptide will be measured using an automated chemiluminescent enzyme immunoassay and run on the DPC Immulite autoanalyser. Proinsulin is the precursor of insulin and the proinsulin-to-insulin ratio will be used to assess the impact of HAART on the processing of proinsulin to insulin within the pancreatic ß-cells. Studies have shown that HAART leads to increases in serum insulin levels [26-28] but it is not known whether this is due to increased production from the ß-cells or reduced clearance by the liver. The proinsulin-to-insulin ratio will be used to assess whether HAART is affecting insulin production from proinsulin whilst C-peptide measurement and use of the insulin-to-C-peptide ratio can be used to assess hepatic clearance. C-peptide has a longer half life in blood than insulin and is removed from circulation by the kidney. Therefore, the insulin-to-C-peptide ratio can be used as a measure of hepatic insulin extraction and C-peptide is a better marker of ß-cell secretory function than insulin itself [53].

Measurement of serum triglyceride, total cholesterol, LDL-cholesterol and HDL-cholesterol (derived from same 10ml red top used above): Triglyceride, total cholesterol and HDL will be measured using an automated colorimetric enzyme assay from Roche (Mannheim, Germany). The assays will be carried out on the Roche Integrer autoanalyser. LDL-cholesterol levels will be calculated using the Friedwald formula [23].

Assessment of insulin resistance: insulin resistance will be assessed using the HOMA method [24]. This is a mathematical formula that derives insulin resistance from the fasting insulin and glucose levels. The formula is: (fasting insulin X fasting glucose) / 22.5. This method has been shown to give good correlations with other techniques used for assessing insulin resistance [24].

Measurement of whole body fat distribution and bone mineral density: The proposed measurements for lipodystrophy will include changes in anthropometric measurements, DEXA scan for adipose tissue levels of arms and legs and MRI scan specifically measuring visceral abdominal fat. Bone mineral density will be evaluated by DEXA scan. Longitudinal follow-up MRI scans (Philips Gyroscan 1.5T, Netherlands) will occur between week 92-96 weeks +/-7 days. The scanning procedure involves acquisition of a sagittal T1-weighted localizer scan with 5mm thickness to identify L4-L5. An axial T1-weighted single slice (5mm thickness) centered at the L4-L5 intervertebral space is then acquired with an FOV of 500mm. The DEXA and MRI scans are both very safe methods of assessing bone mineral density and body fat distribution. DEXA uses low-dose X-rays which, during a single scan, amount to a radiation dose equivalent to a day's background radiation [53]. MRI involves no radiation exposure at all as this technique uses radiofrequency pulses [56]. MRI can therefore be used safely for longitudinal studies for body fat distribution. Time points for body fat distribution (lipoatrophy/lipodystrophy) and bone mineral density will be at the beginning of ART and at the endpoint visit (or earlier at the time of patients discontinued from protocol due to toxicity after > 24 weeks of treatment and randomization). Anthropometric measurements will include: waist-to-hip ratio, mid-upper arm circumference, mid-thigh circumference, chest circumference, and skinfold thickness of triceps, biceps, subscapular, and suprailiac. All measurements will be carried out by one person to

avoid inter-operator variation. Waist-to-hip ratio will be measured by taking hip circumference at the widest circumference of the buttock and waist circumference as the midpoint between the lower rib margin and the iliac crest. Time points for these measurements will be the same as for the collection of blood samples for assessment of metabolic analytes.

C. Data Collection: T cell Subsets and circulating Endothelial Progenitor cells (W. Stevens/L. Montaner)

T cell subsets.
Analysis of T-cell antigen expression will act as our main indication of general immune activation in vivo apart from changes in CBC values or patients symptoms. The justification for each stainings proposed will be:
CD3/CD4/CD45RA/CD62L – Identify changes in the CD4 naïve subset
CD3/CD8/CD45RA/CD62L– Identify changes in the CD8 naïve subset
CD8/CD28/CD38/HLA-DR – Identify Function vs. activation changes in CD8 cells
CD4/CD28/CD38/HLA-DR - Identify Function vs. activation changes in CD4 cells
These stainings will be particularly useful to monitor additional confounding factors such as co-pathogens that may independently weaken immune function or prevent immune reconstitution changes [58].
Time points: The justification for additional data collection in experimental arm is that in contrast to controls who should remain stable during follow-up, we are collecting data before and after each therapy interruption in experimental subjects.
Procedure Whole blood collected in EDTA anticoagulant will be stained with four staining combinations (see above) using directly conjugated antibodies. All antibodies, as well as the appropriate isotype matched controls, are listed in the budget justification text. Briefly, 100ul of whole blood are stained with the appropriate monoclonal antibody mixture and incubated for 15min at room temperature. Erythrocytes are then lysed with FACSLyse ™ lysis buffer (Becton Dickinson FACSlyse, Becton Dickinson Immunocytometry systems, San Jose, CA) for 10 min at room temperature. After being washed twice with PBS the cell pellets are resuspended in 1ml of 2% paraformaldehyde in PBS and analysed on a Beckton Dickinson FACScalibur flow cytometer using the CellQuest software package for acquisition and analysis. A minimum of 10 000 events in a lymphoid gate for each analysis will b e acquired. All events will be stored as listmode data for follow-up analyses at The Wistar Institute upon data file transfers. Analysis of positive events will be performed on cells within a manually drawn lymphocyte gate based on forward and side scatter properties. Where appropriate, thresholds of positive responses will be set according to isotype matched negative controls. Results will be expressed as percent positive of the lymphoid population and/or Mean fluorescence intensity (MFI) as appropriate. We expect to characterize changes in naïve, activated and CD28 bearing T cell subsets in relation to treatment of naïve subjects and treatment interruption.

Circulating endothelial progenitor cells.

200 μl of peripheral blood will be stained with FITC-anti-CD34 (BD biosciences), PE-anti-CD133 (BD Biosciences) and APC-anti-VEGF-R2 (R&D systems) [38] at room temperature for 15 minutes. Fluorochrome-labeled isotype IgG will be used as controls. After 15 min, the sample will be lysed

with Fix and Perm solution (BD biosciences) for 10 min at room temperature, washed and analyzed using a flow cytometer as above. Cell counts will be calculated in reference to white blood cell counts.

D. Data Collection: Immune Response Against Rabies (H. Ertl/ L.J. Montaner)

The justification to analyze rabies responses rests in the unique opportunity to assess the effects of therapy interruption in maintenance of newly acquired humoral and cellular responses as an indication of the host's potential to maintain and mobilize responsiveness against pathogens between continuous and intermittent therapy strategies.

Neutralizing antibody titers (H. Ertl). Serum samples will be tested for neutralizing activity against rabies in vitro infection with a modified version of the rapid fluorescent focus inhibiting test as described by Zalan et al [59]. Human sera from pre- and post-exposure vaccines will be heat treated at 56 ^{o}C for 30 minutes prior to testing. The rabies W.H.O. standard reference serum will be used to standardize analysis from human sera from vaccinees. The CVS-11 virulent established reference strain will be used for this assay at a pre-determined m.o.i. Results will be expressed as amount of neutralization potential with a titer of 0.5IU as a protective titer and the basis by which patient results will be summarized. Data will be analyzed for frequency of responders between groups by expressing results as categorical outcomes of neutralizing versus non neutralizing responses. Dr Ertl's laboratory will perform these assays under the 5% effort of Dr. Xiang in that laboratory. Dr. Ertl's laboratory serves as a USA rabies reference laboratory and a W.H.O. Center for Research on Rabies Virus so all assays proposed are routinely performed.
Lymphoproliferative Responses (L.J. Montaner). PBMC will be cultured (six replicates, 200,000 cells/well) to include rabies stimulation, one unstimulated control and one positive control [PHA, phytoheamoglutinin (5μg/ml, Sigma)], and one negative control [total of 3.6 million PBMC]. After 5 days in culture and an 18hrs [^{3}H]-thymidine pulse, DNA-associated c.p.m. will be assessed and results expressed as stimulation index (SI=Antigen stimulated mean c.p.m./unstimulated mean c.p.m.) with an SI>3 considered positive and as delta c.p.m. (mean c.p.m. antigen stimulated – mean c.p.m. unstimulated control). Dr. Montaner's laboratory will perform these assays as the laboratory has over 4 years experience on weekly assay performance within Wistar's BSL-3 laboratory (Wistar policy requires BSL-3 condition for HIV-infected material).

E. Data Collection: Pre-existing T-cell Recall Responses (L.J. Montaner)

The rationale for collecting data on the maintenance of T-cell responses against additional recall antigens is similar to that pursued with the rabies antigen but extended to pre-existing memory responses. The analysis of de novo rabies and recall responses against pre-existing antigens such as CMV, PPD, *Candida albicans* and HIV-1 will allow for an assessment of the effects of continuous or intermittent therapy on the degree of cellular immune reconstitution present at randomization. We also expect to document the effects of therapy on immune reconstitution between the interval of initiation of therapy and randomization. .

Lymphoproliferative Responses. Cryopreserved PBMC (6 replicates, 200,000 cells/well) will be stimulated with 1 and 5ug/ml of *Candida albicans* lysate, PPD antigen (TB antigen), CMV lysate, and HIV p24 antigen. Negative controls will include medium (Candida and PPD), baculovirus antigen (for HIV-1 P24), uninfected lysates (for CMV). Positive control will be PHA, phytoheamoglutinin (5μg/ml,

Sigma) [total of 8 conditions using 9.6 million PBMC]. After 5 days in culture and an 18hrs thymidine pulse, DNA-associated c.p.m. will be assessed and results expressed as stimulation index (SI=Antigen stimulated mean c.p.m./unstimulated mean c.p.m.) and as delta c.p.m. (mean c.p.m. antigen stimulated – mean c.p.m. unstimulated control). A positive response will be defined as an SI>3. Dr. Montaner's laboratory will perform these assays as the laboratory has over 4 years experience on weekly assay performance with fresh or cryopreserved PBMC within Wistar's BSL-3 laboratory (Wistar policy requires BSL-3 condition for unactivated HIV-infected material).

Cytokine and $Ki67^+$ CD4 and CD8 Recall Frequencies Our ability to monitor antigen –specific frequencies of CD4 and CD8 cytokine- and Ki67-expressing subsets (See Figure 5) in cryopreserved PBMC will be used to analyze responses against HIV-1 p55 and CMV p65 recall antigens. Cryopreserved PBMC will be divided into three groups of 2.5 million cultured with either HIV-1 p55-derived peptides, CMV pp65-derived peptides or medium [total 7.5 million PBMC]. Peptide preparations are 15-mer overlapping by 10. Stimlations will be performed over 72 hours in the presence of anti-CD49d and anti-CD28 monoclonal antibodies followed by a 16 hours re-stimulation in the presence of Brefeldin A (10 ug/ml). Expression of Ki67 (a G1-S marker [93a,]) and IFN-g will be detected on CD4+ T cells by staining with CD3 APC/CD4 PerCP-Cy5.5/Ki67 PE/IFN-γFITC. Surface staining with CD3 and CD4 mAb is performed for 15 min at room temperature. Cells are then permeabilized, washed, and intracellular staining antibodies are added in a final staining step. Finally, cells are washed and data is collected in a BD FACS Caliber by collecting 20,000 CD3+CD4+ cells while assessing the uniformity of the population by CD3 vs. SSC and gating on CD4 cells and assessing the FSC vs. SSC dot plots. CD8 subset data will be collected by gating on $CD3^+/CD4^-$ events. HIV negative PBMC isotype controls and SEB-stimulated compensation controls will be used at the time of data acquisition using CellQuest sofware. The justification for additional data collection in experimental arm is that in contrast to controls who should remain stable during follow-up, we are collecting data before and after each therapy interruption in experimental subjects.

F. Data Collection: Viral Genotypes (L. Morris)

Samples to be analyzed Genotyping will be performed on specimens taken at baseline (before therapy, n=74), and at the first time-point when viral load > 1000 copies/ml is detected during each of the five therapy interruptions in the experimental arm (n=185) and any viral load >1000 copies/ml at week 12 of reinitiated therapy (185 time-points will fit this description for which we will assign an empirical maximum of 20% may require testing , n=37). It is anticipated that up to 20% of total control patients may fail continuous therapy before randomization (n=14). Note the latter is not budgeted for as this would also result in a reduction of budgeted estimates assuming no failures. Resistance assays will not be performed on patients in the control arm unless there is evidence of virologic failure. Specifically, control patients who show 2 consecutive viral blips >1,000 viral copies/ml will be analyzed (anticipated at 20% or 8 of 37). Overall, it is anticipated that a minimum of 267 sequences will be required after reducing sample size to be analyzed due to anticipated drop-out rate. In subjects that are found to have wild-type virologic failure, subsequent on-therapy specimens will be analyzed to determine whether drug selection subsequently becomes evident. In subjects who virologically fail with novel or unexpected mutational patterns, sequencing of additional time-points will be performed. In such cases samples may also be sent for phenotypic testing. Genotypic changes for each individual will be scored as present or absent during each STI (relative to baseline), and correlated with drug exposure and virological outcomes. Since the experimental group will have many more opportunities

to have mutations detected given the more frequent viral rebounds they experience as compared with the controls, these results will bias the study toward finding the emergence of resistance mutations in the experimental group. However, since safety is one of the important end-points of this study over-sampling of the experimental group is justified.

Methodological approach HIV-1 drug resistance will be performed using the ViroSeqTM HIV-1 Genotyping System, v2 (Applied Biosystems, Foster City, CA). Briefly 500 μl of plasma collected in EDTA is spun at 23,500g for 1 h at 4°C to concentrate viral particles. Viral pellets are lysed using guanidine thiocyanate and the RNA precipitated with isopropanol and ethanol. 10 μl of purified RNA is reverse-transcribed into cDNA with Moloney Murine Leukemia Virus (MuLV) RT at 42°C for 60 min. A 1.8 Kb fragment is then amplified by PCR using pol-specific forward and reverse primers and AmpliTaq Gold DNA polymerase. This fragment spans the entire PR gene (amino acids 1-99) and two-thirds of the RT gene (amino acids 1-335). The PCR conditions are 93°C for 20 sec, 64°C for 45 sec, 66°C for 3 min for 40 cycles followed by 72°C for 10 min and cooling at 4°C. PCR products are purified using spin columns and run on a 1% agarose gel containing ethidium bromide. A semi-quantitative DNA concentration is determined by comparing fluorescence of the sample to a DNA Mass Ladder. A total of six sequencing primers are used (A, B and C - forward primers and E, F and G - reverse primers. Primer D is not used as we have found that it does not amplify subtype C samples) together with premixed BigDye sequencing reagents. 12 μl of each primer is combined with 8 μl of sample in a 96-well plate and placed in a thermocycler for 25 cycles of 96°C for 10 sec, 50°C for 5 sec, 60°C for 4 min. Sequencing reactions are purified using Sigma 96-well spin plates. The plate is loaded directly into the 3100 instrument after heat denaturation and electropohoresed for approximately 6 hours.

Rationale. The advantages of the ViroSeq Genotyping System include the: (a) ability to amplify low copy number, in some cases less than 1,000 copies RNA/ml. This will be important for samples from individuals with low viral loads especially those on ARV treatment; (b) use of a single-cycle PCR rather than a nested PCR which has a high risk of contamination; (c) use of AmpErase UNG to control for contamination with previously amplified material; (d) high throughput of the 16-capillary ABI 3100 machine which allows for 32 patients to be done every 12 hours (32 x 6 reactions per patient = 192 reactions on two 96-well plates); (d) use of primers and PCR reactions that have been tested and shown to function efficiently for local HIV-1 subtype C pol genes (with the exception of primer D which is not used); (e) data from 3 forward and 3 reverse primers gives sufficient coverage to ensure double-stranded sequence data; (f) generation of sequence data from a large segment of the pol gene. This allows for subtype designation (to confirm they cluster with subtype C), viral evolutionary and linkage studies between samples from the same patient as well as for detection of possible contamination; (g) extensive use and familiarity with the ABI 3100 as well as research personnel trained in analyzing sequence information; and (h) use of research personnel trained by Applied BioSystems in the performance of the ViroSeq drug resistance assay.

Quality control. The ViroSeq HIV-1 Genotyping System includes a number of quality checks: (a) the forward and reverse orientations of the DNA sequence are checked for nucleotide base concordance; (b) mismatches are highlighted to allow for manual editing; (c) areas of single-stranded coverage are highlighted for repetition if necessary and; (d) the final report indicates the total number of mismatches before and after editing to give an indication of the quality of the sequence data. Contamination will be rigorously guarded against by performing simultaneous phylogenetic analysis

on all samples as well as other sequences being generated at the same time from other projects. Negative controls will be included in all batches of RNA extractions and PCR's.

G. Data collection: communication and storage

The data collection infrastructure is being developed under the coordination of the Wistar Information Technology Department. A dedicated server will be installed at Wistar Institute to store data files and host SQL tables; this will allow for multi-user access (PIs, Dr. Azzoni, database manager, bioinformatics and biostatistics personnel). SQL tables will be managed using MS Enterprise Manager; all stored data will be backed up automatically based on a fixed schedule.

Site data will be collected in Access database tables (I. Sanne, W. Stevens, L. Montaner) or Excel spreadsheets (N. Crowther, L. Morris). 128-bit encrypted incremental or complete updates of these tables will be sent weekly from the remote sites to the central location via secure ftp, to a dedicated server. From there the tables will be automatically uploaded to the central ODBC database (SQL server) located in a non-remotely accessible server (tables generated at the Wistar Institute will be uploaded directly to the SQL server). The entire database (which will not contain any personal identifier) will be copied daily to a mirror open server, which will be remotely accessible to all study group sites (using regular password protection). By strictly regulating the flow of information, this architecture is expected to protect the central location from unauthorized access.

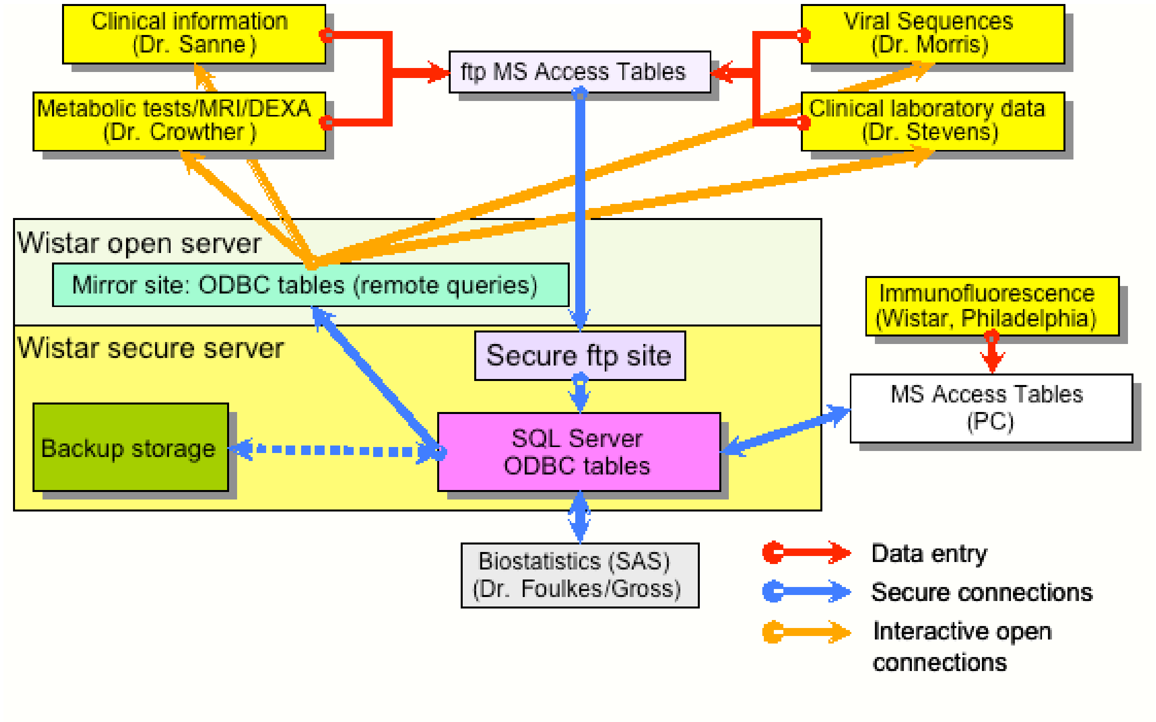

Information management . Schematic representation of the flow of information between th e central site (Wistar Institute, Philadelphia) and remote sites.

Where necessary, specific data (e.g. FACS analysis results) will be processed and analyzed prior to storage. "Raw" data (e.g. machine output files) will be stored locally on solid state media (e.g. CD-RW or tape). Analytical output tables/files will be stored centrally, both in the original format and as uploaded SQL records.

To protect the data from unauthorized access, access to the web server will be restricted by means of encryption, firewalls and password-restriction. Access to the server hosting the SQL tables will be directed via a secure web connection to a front server.

In order to generate the tables that will be used for the statistical analyses, MS Access will be used to query linked SQL tables.

Document Storage

CRFs and other forms/paperwork will be stored in subject-specific binders at each clinic/site. The binders will be stored by each clinic in a secure and hazard free data storage environment, with double lock. Access will be restricted to study personnel authorized to handle research documents.

H. Clinical Site Monitoring and Record Availability

Site monitors will be provided under contract by the National Institute of Allergy and Infectious Diseases (NIAID). Monitors will visit participating clinical research sites to review the individual subject records, including consent forms, CRFs, supporting data, laboratory specimen records, and medical records (physicians' progress notes, nurses' notes, individuals' hospital charts), to ensure protection of study subjects, compliance with the protocol, and accuracy and completeness of records. The monitors also will inspect sites' regulatory files to ensure that regulatory requirements are being followed and sites' pharmacies to review product storage and management.

The investigator will make study documents (e.g., consent forms, drug distribution forms, CRFs) and pertinent hospital or clinic records readily available for inspection by the local IRB, the site monitors, the NIAID, the Office for Human Research Protections (OHRP), the pharmaceutical sponsor(s), or the sponsor's designee for confirmation of the study data.

XII. Safety reporting

Expedited Adverse Event (EAE) Reporting

A "targeted" expedited adverse event (EAE) reporting requirement wil be used oin this study. All guidelines, and definitions for expedited reporting of adverse events (AEs) for this approach as reported to the DAIDS Safety Office through the Regulatory Compliance Center (RCC) are defined in The Manual for Expedited Reporting of Adverse Events to DAIDS (Version May 6, 2004) and December 2004 Toxicity Tables.

These AEs must be documented on the DAIDS EAE Form available at the RCC website at **HTTP://RCC.TECH-RES-INTL.COM/**.

In addition to submitting EAE information to the DAIDS Safety Office through the RCC, the site investigator is required to submit AE information as required by local regulatory agencies or other local authorities.

This study uses the targeted level of expedited AE reporting as defined in the Manual. This level of reporting is required for the entire study duration (from enrollment until the study participant completes or discontinues the study)

The study agents requiring expedited reporting of AEs are:

Verorab®
Stavudine (D4T)
Lamivudine (3TC)
Lopinovir/Ritonovir
Zidovudine (AZT)

In addition to the above events the DAIDS requires that the following events also be reported to the RCC as defined in the EAE manual:

- "Unexpected, serious suspected adverse drug reactions (SADERS) that occur at any time after the protocol-defined expedited reporting period if the study staff become aware of its occurrence, and
- We will be reporting to RCC any targeted unexpected adverse events. All other serious adverse events if not reportable by EAE criteria, including opportunistic infections during the treatment interruption phase if not associated with a clinical SAE, will not be reported but will be captured."

The Division of AIDS December 2004 Table for Grading Severity of Adverse Events must be used for determining and reporting the severity of AEs. [A protocol team developing Adverse Event Grading scheme(s) for parameter(s) NOT found on the DAIDS Table would define the parameter(s) here or cite the location of the parameter in the document here.] The Division of AIDS December 2004 Table for Grading Severity of Adverse Events table **II** is/are available at the RCC website at **HTTP://RCC.TECH-RES-INTL.COM/** or is/are located in the Manual of Operations for this study.

Only adverse events which occur after the initiation of antiretroviral therapy will be documented. Adverse events occurring before treatment initiation will become part of medical history.

Laboratory monitoring procedures will focus on regular complete full blood cell counts, renal function, liver function and enzymes and pancreatic amylase if needed (see below). At each clinical visit doctors will take a full history of any adverse events, perform a clinical examination, monitor for anemia before research blood draws and all drug related toxicities. A record of concomitant medication will be maintained other than over the counter medication unless the investigator feels this was used to treat an adverse event. If the subject experiences any side effects or other symptoms between scheduled study visits, the subject will be instructed to report them immediately to the research physician or research nurse. A 24-hour on-call system is in place to ensure patients can contact one of the investigators at all times.

Procedures for Reporting of Serious Adverse Events:

The principle investigator of the site is responsible for ensuring the timely (within 72 hours after site recognition) reporting of SADERS. An EAE form detailing the unexpected AE must be

completed by the investigator. The completed form may be considered source document where the information is the first record of the patient information. Forms will be faxed as SAE submission reports to the MCC, NIH Compliance Center [see below] and Ethics Committees, and Wistar Institute, reporting the details of each of the EAEs.

All completed DAIDS Expedited Adverse Event Forms are submitted to the DAIDS Safety Office. For questions or other communication, please note the following:

Website:	http://rcc.tech-res-intl.com
Office Phone*:	1-800-537-9979 (US only) or +1-301-897-1709
Office Fax*:	1-800-275-7619 (US only) or +1-301-897-1710
Office Email:	RCCSafetyOffice@tech-res.com
Office Hours:	Monday through Friday, 8:30 AM to 5:00 PM<br>(US Eastern Time)
Mailing Address:	DAIDS Safety Office<br>6500 Rock Spring Drive<br>Suite 650<br>Bethesda, MD 20817

*Office phone and fax are accessible 24 hours per day.

Monthly Summary Adverse Experience Safety Reviews

Both new and cumulative adverse event data should be summarized monthly, by organ system and not by arm, and will be reviewed by the medical officer and blinded to the principle investigators. This report will be prepared at the Clinical site (Data Manager) and posted for medical officer review.

Monitoring Committee Reviews – Data Safety Monitoring Board:

Interim safety monitoring reports will be generated yearly by the study statisticians and Wistar data managers for review by the NIH Data Safety Monitoring Board (DSMB). Reports will include patient files with CD4 count, viral load, clinical HIV disease progression, non-HIV related events that may cloud the endpoint analysis (patient recruitment and drop-outs) and safety data by arm and broken out by organ system, as well as all EAE reports. The DAIDS DSMB primary objective will be to review such data and make independent recommendations to DAIDS concerning possible modifications needed in the conduct of the study, particularly in reference to the safety of the protocol. In addition, DAIDS DSMB will monitor factors (such as rate of accrual and loss to follow-up) that impact the feasibility of timely completion of the study. Aggregate data for SAEs will not be available to study investigators during interim safety monitoring reports.

Specifically, the data to be reviewed will include:

Screening data
Baseline data
Safety data
Clinical data
Quality assurance data
Accrual status including projections
Total number of case report forms that are in-house
Total number of case report forms that have been quality assured
Other data that will help in the assessment of the effectiveness of the clinical trial

DSMB yearly reports by the data manager (Wistar) and the study statisticians and blinded to the principle investigators.

ADDITIONAL PERIODIC SAFETY MONITORING REVIEW

This study will be subject to the annual review policies of: (1) the Medicines Control Council of South Africa and Relevant Ethics Committees, (2) University of Witwatersrand and (3) Wistar IRBs.

XIII. Human Subjects

All the procedures described here will be submitted for approval by the Institutional Review Board of the Wistar Institute following the review/approval by the Human Research and Ethics Committee of the University of the Witwatersrand, Johannesburg South Africa (Federal Assurance # SF, IORG 000862, IRB 00001223).

Recruitment, Institutional Review Board (IRB) Review and Informed consent:

Prior to implementation of this protocol, all sites will have the protocol and consent form approved by their local institutional review board (IRB). Sites will be registered with and approved by the DAIDS/Regulatory Operations Center, Regulatory Compliance Center (RCC) Protocol Registration Office. Site registration will occur before any subjects can be enrolled in this study. Once a candidate for study entry has been identified, details will be carefully discussed with the subject. The subject will be asked to read and sign the consent form that was approved by the two sites IRBs, the Medicines Control Council (MCC) and the DAIDS/Regulatory Operations Center, Regulatory Compliance Center (RCC) Protocol Registration Office.

This protocol and the informed consent document (Appendix A) and any subsequent modifications will be reviewed and approved by the IRB or ethics committee responsible for oversight of the study. Eligible participants will be identified from patients already diagnosed to be HIV positive, and for whom a CD4 cell count is available from the primary care clinic. A signed consent form will be obtained from the subject. A copy of the consent form will be given to the subject, parent, or legal guardian, and this fact will be documented in the subject's record. The consent form will provide details of the purpose of the study, the study procedure, potential risks, right to withdraw consent,

compliance information, and description of the treatment strategy, provision of ART and of the post-trial treatment. Before signing, all subjects will be questioned for comprehension of consent form (it is assumed that subjects will be able to write and read), given the opportunity to ask questions and shown an anticipated visit schedule over the 156 weeks of the study to confirm understanding of the study risks and time commitment involved with participation. During the course of the study, subjects will be asked to reconfirm or decline their consent at each study visit. Documents will be written in English, Zulu, Sesotho, Xhosa and Afrikaans. All translations will be verified according to the current verification statement.

Subject Confidentiality:

All laboratory specimens, evaluation forms, reports, and other records that leave the site will be identified by coded number only to maintain subject confidentiality. All records will be kept locked in a secure location. All computer entry and networking programs will be done with coded numbers only. Clinical information will not be released without written permission of the subject, except as necessary for monitoring by IRB, the FDA, the NIAID, the OHRP, the pharmaceutical supporter(s), or the supporter's designee.

Potential risks & Precautions to minimize risks:

Potential risks identified include the development of ART complications such as drug intolerance during continuous therapy or drug resistance during periods of drug interruption or reintroduction, vaccination in the presence of HAART, blood draw associated complications and breach of confidentiality. The DEXA and MRI scans are both very safe methods of assessing bone mineral density and body fat distribution. DEXA uses low-dose X-rays which, during a single scan amount to a radiation dose equivalent to a day's background radiation [53]. MRI involves no radiation exposure at all as this technique uses radiofrequency pulses [56]. DEXA and MRI can therefore be used safely for longitudinal studies of bone mineral density and body fat distribution with minimal risk to subjects. Both long term and short term side effects of antiretroviral therapy will be described to the patients for self-reporting and monitored by qualified personnel. Laboratory safety blood sampling will include regular testing of haematology and biochemistry testing. Regular clinic visits with doctors assessing the patients will be undertaken with frequent monitoring for identified study risks. Each patient will also be made aware of complications and asked to contact the clinical investigators outside study visits if symptoms develop. Although vaccination in the presence of HAART could result in an increase in viral replication, reports to date on the use of this vaccine under HAART have not supported this potential outcome. The study also includes confirmation of suppression to <50 copies/ml before proceeding with randomization and the first interruption if in experimental arm. Subjects may also feel discomfort during blood draws or experience bruising at the needle site. The clinical investigators will be responsible for oversight of all patients' procedures. Confidentiality will be protected by restricting patient identifiers to the clinical site and assigning a code to samples when shipped. Access to personal information will be restricted by password and stressed through confidentiality agreements with key personnel as to the risks of disclosure. All contact information provided at entry will be reviewed with patients to obtain consent as to how future contacts, (e.g., confirmation of appointments, etc.) should proceed. All identifiers and records will be kept in locked files within the clinic in accordance with strict confidentiality guidelines at the clinical site.

Risk-benefit ratio:

All participants will benefit from access to treatment with a potent combination antiviral therapy regimen before the time they would be elegible to start in the National treatment program and receiving rabies vaccine. All subjects after cessation of study would transition to the National program maintaining drug supply so the benefit is a minimum of 3.5 years of delay in disease progression in all participants that can be successfully suppressed as a result of the therapy and clinical care offered in this study. The risks of treatment failure due to treatment toxicity or viral resistance, is not considered significantly greater in either treatment arm, providing the equipoise for this study. Benefit of having access to earlier therapy may be counterbalanced by the potential of therapy failure due to resistance and toxicity at an earlier stage and the increased risk of resistance in the therapy interruption arm.

Subjects' participation will also provide altruistic benefit for the contribution to exploring new strategies for treatment that are specifically suited for resource poor settings. While the provision of life long therapy is primarily limited by resources, the global expansion of access to antiretroviral therapy inclusive of South African Department of Health provision plans makes the continued treatment of enrolled subjects very likely. Therefore, subjects will have the altruistic benefit that participation on this study will may benefit therapy access programs by assessing the safety and efficacy of sequenced treatment interruptions as a strategy to extend the sustainability and cost of a single regimen.

Study equipoise with regards to risk of therapy interruption strategies tested to date as compared to this study is further expanded upon in Background under Section C.

Uninfected donor standard samples:

a) National Institute for Communicable Diseases

Normal blood donor samples used in the National Institute for Communicable Diseases in Johannesburg are obtained from material collected for non-research purposes by the blood bank and provided without any donor identifiers on a cost-recovery program. These samples will be submitted for exemption of a need for informed consent following the approval by the University of the Witwatersrand IRB.

b) The Wistar Institute

Peripheral blood from healthy donors will be obtained from paid volunteers, who consent and donate up to one pint of blood at the Wistar Institute. Blood is drawn in a designated room by a professional phlebotomist employed by the Wistar Institute. Donors are recruited by the phlebotomist who obtains informed consent on an approved form before each draw following specific IRB-approved procedures. The identity of the donors is not made known to the investigators. All donors are screened for evidence of infection by HIV-1 or Hepatitis B virus before donating blood. Our present volunteer blood donors range in age from 18 to 50 years, with the majority of donors in the 25 to 35 years.

Study Discontinuation

The study may be discontinued at any time by the IRB, investigators, the NIAID, or other government agencies as part of their duties to ensure that research subjects are protected.

Publication of Research Findings

Publication of the results of this trial will be governed by policies of DAIDS as agreed to in the subcontract between the Wistar Institute and the Investigators.

XIV. BIOHAZARD CONTAINMENT

As the transmission of HIV and other blood-borne pathogens can occur through contact with contaminated needles, blood, and blood products, appropriate blood and secretion precautions will be employed by all personnel in the drawing of blood and shipping and handling of all specimens for this study, as currently recommended by the Centers for Disease Control and Prevention and the National Institutes of Health.

All infectious specimens will be transported using packaging mandated in the Code of Federal Regulations, CDC 42 CFR Part 72. Please also refer to individual carrier guidelines, e.g., FedEx, Airborne, for specific instructions.

XV. Department of Health Support

A national roll-out plan for antiretroviral therapy is currently in the implementation phase in South Africa. The clinical research site will engaged in the DOH treatment pilot sites.

Obstacles to treatment implementation include the lack of trained personnel, the cost of antiretroviral therapy, drug distribution mechanisms and laboratory monitoring cost, facilities and distribution. Many of these constraints are addressed in this research program. Of particular concern is the distribution and availability of trained doctors. The Department of Health is targeting the primary and secondary health care setting for the treatment of HIV/AIDS including antiretroviral therapy. As a potential solution, it is believed that training of clinical personnel such as primary health care sisters to monitor treatment can lead to a more rapid escalation of treatment access, wider geographic spread and potentially better treatment adherence. Reducing the cost of HIV treatment both for drugs and laboratory monitoring is considered a priority.

Community Support

This study has been presented to the Community Advisory Board of the ACTG site for discussion. Broad support for this study has been received.

XVI. REFERENCES

1. *Global AIDS Research Initiative Strategic Plan.* Office of AIDS Research, National Institute of Health, 2000.
2. Stephenson, J., *AIDS in South Africa takes center stage.* Journal of American Med Ass, 2000. **284**: p. 165.
3. Williams, B.G., Gouws, E., Colvin, M., Sitas, F. Ramjee, G., Abdool Karim, S.S., *Patterns of Infection: Using age prevalence data to understand the epidemic of HIV is South Africa.* S. African Journal of Science, 2000. **96**.
4. Health, D.., *National HIV sero-prevalence Survey of Women Attending Public Antenatal Clinics in South Africa*. 2000, Health Systems Research and Epidemiology, Dept. of Health Pretoria.
5. Corbett, E.L., *Mycobacterial disease in South Africa gold miners: association with HIV infection and occupational lung disease*. London School of Hygiene and Tropical Medicine. 1999, London.
6. Murphy, R.L., et al., *ABT-378/ritonavir plus stavudine and lamivudine for the treatment of antiretroviral-naive adults with HIV-1 infection: 48-week results.* Aids, 2001. **15**(1): p. F1-9.
7. Walmsley, S., et al., *Lopinavir-ritonavir versus nelfinavir for the initial treatment of HIV infection.* N Engl J Med, 2002. **346**(26): p. 2039-46.
8. Morris, L., Bredell, H., van Harmelen, J., Ping L., Pasqual, A., Ramjee G., Abdool Karim, S., Gray G., McIntyre, J., Maartens, G., Swanstrom, R., and Williamson, C., *No evidence for naturally occurring resistance mutations to HIV-1 reverse-transcriptase inhibitors among South African HIV-1 subtype C isolates.* S. African Journal of Science, 2000. **96**: p. 369-370.
9. Pillay, C., et al., *HIV-1 subtype C reverse transcriptase sequences from drug-naive pregnant women in South Africa.* AIDS Res Hum Retroviruses, 2002. **18**(8): p. 605-10.
10. Morris L, P.C., Dirr H, *Reverse Transcriptase (RT) and Protease (PR) Sequences from Drug-Naive Pregnant Women in South Africa.* Antiviral Research, 2002. **7**: p. S145.
11. Pillay C, G.G., Stevens G, Jivkov B, Violari A, Stevens W, McIntyre J, Morris L, *Emergence of drug resistance mutations in children treated with ddI and d4T after treatment to prevent mother to child transmission.* Antiviral Research, 2002. **7**: p. S61.
12. Paterson, D.L., et al., *Adherence to protease inhibitor therapy and outcomes in patients with HIV infection.* Ann Intern Med, 2000. **133**(1): p. 21-30.
13. Gross, R., et al., *Effect of adherence to newly initiated antiretroviral therapy on plasma viral load.* Aids, 2001. **15**(16): p. 2109-17.
14. Wendel, C.S., et al., *Barriers to use of electronic adherence monitoring in an HIV clinic.* Ann Pharmacother, 2001. **35**(9): p. 1010-5.
15. Orrell, C., et al., *Adherence is not a barrier to successful antiretroviral therapy in South Africa.* Aids, 2003. **17**(9): p. 1369-75.
16. van der Merwe, M.T., et al., *Evidence for insulin resistance in black women from South Africa.* Int J Obes Relat Metab Disord, 2000. **24**(10): p. 1340-6.
17. Buthelezi, E.P., et al., *Ethnic differences in the responsiveness of adipocyte lipolytic activity to insulin.* Obes Res, 2000. **8**(2): p. 171-8.
18. Punyadeera, C., et al., *Ethnic differences in lipid metabolism in two groups of obese South African women.* J Lipid Res, 2001. **42**(5): p. 760-7.
19. van der Merwe, M.T., et al., *Metabolic indices in relation to body composition changes during weight loss on Dexfenfluramine in obese women from two South African ethnic groups.* Int J Obes Relat Metab Disord, 1996. **20**(8): p. 768-76.

20. Punyadeera C, C.N., van der Merwe M-T, Toman M, Immelman AR, Schlaphoff GP, Gray IP, *The influence of visceral adiposity on glucose and lipid metabolism in obese black and white South African Type 2 diabetic patients.* J Endocrinol Metab Diabet S Afr, 2002. **7**(3): p. 99-108.
21. Kissebah, A.H. and G.R. Krakower, *Regional adiposity and morbidity.* Physiol Rev, 1994. **74**(4): p. 761-811.
22. Punyadeera, C., et al., *Weight-related differences in glucose metabolism and free fatty acid production in two South African population groups.* Int J Obes Relat Metab Disord, 2001. **25**(8): p. 1196-205.
23. Friedewald, W.T., R.I. Levy, and D.S. Fredrickson, *Estimation of the concentration of low-density lipoprotein cholesterol in plasma, without use of the preparative ultracentrifuge.* Clin Chem, 1972. **18**(6): p. 499-502.
24. Matthews, D.R., et al., *Homeostasis model assessment: insulin resistance and beta-cell function from fasting plasma glucose and insulin concentrations in man.* Diabetologia, 1985. **28**(7): p. 412-9.
25. Miller, K.D., et al., *Visceral abdominal-fat accumulation associated with use of indinavir.* Lancet, 1998. **351**(9106): p. 871-5.
26. Carr, A., et al., *A syndrome of peripheral lipodystrophy, hyperlipidaemia and insulin resistance in patients receiving HIV protease inhibitors.* Aids, 1998. **12**(7): p. F51-8.
27. Vigouroux, C., et al., *Diabetes, insulin resistance and dyslipidaemia in lipodystrophic HIV-infected patients on highly active antiretroviral therapy (HAART).* Diabetes Metab, 1999. **25**(3): p. 225-32.
28. Hadigan, C., et al., *Metabolic abnormalities and cardiovascular disease risk factors in adults with human immunodeficiency virus infection and lipodystrophy.* Clin Infect Dis, 2001. **32**(1): p. 130-9.
29. Oxenius, A., et al., *Stimulation of HIV-specific cellular immunity by structured treatment interruption fails to enhance viral control in chronic HIV infection.* Proc Natl Acad Sci U S A, 2002. **99**(21): p. 13747-52.
30. Oxenius, A., et al., *Stimulation of HIV-specific cellular immunity by structured treatment interruption fails to enhance viral control in chronic HIV infection.* Proc Natl Acad Sci U S A, 2002. **99**(21): p. 13747-52.
31. Autran, B., et al., *Positive effects of combined antiretroviral therapy on CD4+ T cell homeostasis and function in advanced HIV disease [see comments].* Science, 1997. **277**(5322): p. 112-6.
32. El-Sadr, W. and J. Neaton. *Antiretroviral therapi II: new insights and treatment strategies*. in *13th conference on retroviruses and opportunistic infections*. 2006. Denver, CO.
33. de Larranaga, G.F., et al., *Viral load and disease progression as responsible for endothelial activation and/or injury in human immunodeficiency virus-1-infected patients.* Blood Coagul Fibrinolysis, 2003. **14**(1): p. 15-8.
34. de Larranaga, G.F., et al., *Endothelial markers and HIV infection in the era of highly active antiretroviral treatment.* Thromb Res, 2003. **110**(2-3): p. 93-8.
35. Fisher, S.D., T.L. Miller, and S.E. Lipshultz, *Impact of HIV and highly active antiretroviral therapy on leukocyte adhesion molecules, arterial inflammation, dyslipidemia, and atherosclerosis.* Atherosclerosis, 2006. **185**(1): p. 1-11.
36. Hill, J.M., et al., *Circulating endothelial progenitor cells, vascular function, and cardiovascular risk.* N Engl J Med, 2003. **348**(7): p. 593-600.
37. Vasa, M., et al., *Increase in circulating endothelial progenitor cells by statin therapy in patients with stable coronary artery disease.* Circulation, 2001. **103**(24): p. 2885-90.
38. Werner, N., et al., *Circulating endothelial progenitor cells and cardiovascular outcomes.* N Engl J Med, 2005. **353**(10): p. 999-1007.

39. Hsue, P.Y., et al., *Progression of atherosclerosis as assessed by carotid intima-media thickness in patients with HIV infection.* Circulation, 2004. **109**(13): p. 1603-8.
40. Seftel, H.C., et al., *Selected risk factors for coronary heart disease in male scholars from the major South African population groups.* S Afr Med J, 1993. **83**(12): p. 891-7.
41. Thisyakorn, U., Pancharoen, C., Ruxrungtham, K., Ubolyam, S., Khawplod, P., Tantawichien, T., Phanuphak, P., Wilde, H., *Safety and Immunogenicity of Pre-exposure rabies vaccination in children infected with HIV type 1.* Clin Infect Dis, 2000. **30**: p. 218.
42. Thisyakorn, U., Pancharoen, C., Wilde, H., *Immunologic and virologic evaluation of HIV-1 infected childred after rabies vaccination.* Clin Infect Dis, 2001. **19**: p. 1534-1537.
43. Jaijaroensp, W., Tantawichien, T., Khawplod, P., Tepsumethanon, S., Wilde, H., *Postexposure rabies vaccination in patients infected with human immunodeficiency virus.* Clin Infect Dis, 1999. **28**: p. 913-914.
44. Kositprapa, C., et al., *Immune response to simulated postexposure rabies booster vaccinations in volunteers who received preexposure vaccinations.* Clin Infect Dis, 1997. **25**(3): p. 614-6.
45. Smith, J.S., P.A. Yager, and G.M. Baer, *A rapid reproducible test for determining rabies neutralizing antibody.* Bull World Health Organ, 1973. **48**(5): p. 535-41.
46. Follman, D., *On the effect of treatment among treatment compliers: An analysis of the Multiple Risk Factor Intervention Trial.* Journal of the American Statistical Association, 2000. **95**: p. 1101-1109.
47. Armitage, P. and G. Berry, *Statistical Methods in Medical Research*. 2nd ed ed. 1987, Oxford: Blackwell Scientific Publications.
48. Piantadosi, S., in *Clinical Trials:A Methodological Perspective*, Wiley: New York. p. 276-277.
49. Tebas, P., et al., *Accelerated bone mineral loss in HIV-infected patients receiving potent antiretroviral therapy.* Aids, 2000. **14**(4): p. F63-7.
50. Diggle, P.J., Liang, K.Y., Zeger, S.L., *Analysis of Longitudinal Data*. 1994, New York: Oxford University Press.
51. Zhang, D. and M. Davidian, *Linear mixed models with flexible distributions of random effects for longitudinal data.* Biometrics, 2001. **57**(3): p. 795-802.
52. Vrijens, B., Goetghebeur, E., *Comparing compliance patterns between randomized treatments.* Control Clin Trials, 1997. **18**(3): p. 187-203.
53. Jebb, S., *Measuring body composition:from the laboratory to the clinic*, in *Clinical Obesity*, P. Kopelman and M. Stock, Editors. 1998, Blackwell Science: Oxford. p. 18-49.
54. Carr, A., et al., *Diagnosis, prediction, and natural course of HIV-1 protease-inhibitor-associated lipodystrophy, hyperlipidaemia, and diabetes mellitus: a cohort study.* Lancet, 1999. **353**(9170): p. 2093-9.
55. Carr, A. and D.A. Cooper, *Adverse effects of antiretroviral therapy.* Lancet, 2000. **356**(9239): p. 1423-30.
56. van der Kooy, K. and J.C. Seidell, *Techniques for the measurement of visceral fat: a practical guide.* Int J Obes Relat Metab Disord, 1993. **17**(4): p. 187-96.
57. Chambless, L.E., et al., *Association of coronary heart disease incidence with carotid arterial wall thickness and major risk factors: the Atherosclerosis Risk in Communities (ARIC) Study, 1987-1993.* Am J Epidemiol, 1997. **146**(6): p. 483-94.
58. Clerici, M., et al., *Immune activation in africa is environmentally-driven and is associated with upregulation of CCR5. Italian-Ugandan AIDS Project.* Aids, 2000. **14**(14): p. 2083-92.
59. Zalan, E., C. Wilson, and D. Pukitis, *A microtest for the quantitation of rabies virus neutralizing antibodies.* J Biol Stand, 1979. **7**(3): p. 213-20.

XVII Appendix A
PARTICIPANT INFORMATION LEAFLET AND INFORMED CONSENT FORM

Division of Aids Research
National Institute of Health,(NIH) USA

TRIAL TITLE:

'A Randomised Clinical Trial Assessing Continuous HAART versus Interrupted HAART in a Resource Poor Clinic'

PROTOCOL NUMBER:
DAIDS-ES ID 10428; RO1 AI 51986-01

INTRODUCTION
You are being invited to take part in this research study. The Clinical Investigator is studying the effects of you taking your HIV medication all the time (we will refer to this as continuous treatment) as compared to short periods of time (we will refer to this as intermittent treatment). This means that we are studying the risk and benefits of stopping all your anti-HIV treatment at scheduled time-points over approximately 2.5 years. **Fifty two adults infected with HIV are expected to complete** this study.

Please take as much time as you need to ask any questions and to discuss this study with the doctors. For your safety, you should inform the clinic where you are treated and all the doctors looking after you that you are participating in this clinical trial. Your decision to take part in this study is voluntary, if you decide not to take part in this study your current treatment in the HIV clinic will not be changed or stopped.

BACKGROUND INFORMATION
In this study we are trying to find out if it is safe and useful to take people off their HIV medicines every few weeks, under their doctor's supervision, or if they need to take their medications all the time.
When HIV positive people need treatment, a combination of drugs (we will call it **ART**) is usually given without interrupting. This medicine will keep the levels of the virus in the blood very low, which gives the body a chance to get better. ART has the ability to slow or stop the disease from getting worse, and become AIDS. However, taking ART has also some problems. In the first place,, ART cannot completely wipe out the virus from the body, and if you stop taking them the HIV in your blood will most likely become high again. Second, ART may result in side-effects and this risk may increase if you take the drugs for a long time. Third, the virus can become resistant to ART, and become high again even if you keep taking the drugs. Fourth, ART is expensive and drug supplies may be interrupted.

<u>**Immune Responses:**</u> Your body is normally kept free from infections by many specialized cells, which we call the **immune system**. It is important that your immune system is strong to control infections to which your body is exposed. Based on studies of the few persons infected with HIV that do not develop AIDS, we believe that the better your immune system responds to diseases

the smaller the chance of developing AIDS.

In the past 5 years we have studied people with HIV in Philadelphia (USA): our studies tell us that therapy interruptions, similar to what we plan to do in this study, do not cause a damage of the immune system and do not make our patients develop AIDS . However, in some other studies, long duration treatment interruptions (more than 3 months) have caused infections and in some cases cardiovascular problems such as heart attacks, strokes or chest pain. In this study you may be required to stop taking your medication for up to 8 weeks, which is less than what these other studies have used. **Stopping anti-HIV drugs usually causes HIV levels in the blood to go up and CD4 counts to go down, and your risk of having complications from HIV disease increases. Your risk to infect others also increases during therapy interruption due to the return of the virus.** We will monitor your health closely during this period, and if your doctor feels that the interruption is a threat to your health, he/she will ask you to start taking your medications immediately. You are also free to decide not to go ahead with an interruption, or to resume your medication at any time during the interruption. If you decide not to interrupt your medication, you will not be part of the study, but your medications will be provided anyway.

This study will test whether interruptions in therapy can still maintain the same level of immune function as if therapy was taken all the time. In order to study your immune systems ability to respond to disease while on this study, we will vaccinate you against rabies, as described further below, and study your body response to the vaccine. Rabies is an infection, which can occur after an animal bite: in South Africa veterinarians and handlers of wild animals are usually vaccinated against rabies. This vaccination has been shown to be safe for HIV positive people.

DURATION OF THE TRIAL

The study is planned to continue for *two years and six months, 21 visits for the continuous arm, 25 visits for the interrupted arm.* Each study visit should be approximately 45-60 minutes duration.

Screening visit
(Are you eligible for the study, do you meet the criteria of the study?)
⇓
Baseline visit
(if you are eligible then treatment will begin)
⇓
36+ weeks of ART treatment
(24 weeks need to be with HIV Viral load <50 c/ml; Rabies vaccination given at weeks 16,17,22)
⇓
Randomization
(you are assigned randomly to either a group of participants on interrupted ART or a group on continuous ART if HIV viral load is <50 c/ml and CD4 count is >450)
⇓
Treatment
(either continuous or interrupted, described in detail below)
⇓
End of the trial

If you cannot attend the frequency of visits due to your life-style or other commitments, **you should not participate in this study**.

TRIAL PROCEDURES

If you agree to participate, you will receive the following drugs against HIV (ART): Lopinavir / Ritonavir , Lamivudine and Stavudine. You will also be requested to complete a series of questions each clinical visit to find out how the therapy is affecting your ability to work and daily living. Participants who are being treated on the study with Lopinovir/Ritonavir and who develop Tuberculosis, will use Rifibutin to treat the TB instead of Rifampicin. Rifibutin will be supplied by the trial. If you experience side effects from taking Stavudine, you will be able to receive Zidovudine instead.

Screening Visit: For your first visit on the study, you will be asked to attend the Clinic at the Research Centre. The doctors working with Dr Sanne will then go through your medical history, and do a physical examination to make sure that it is safe for you to participate in the study, and that you qualify for the study. If you qualify you will be asked to complete tests to look at the fat and bone composition of your body before you start ART by completing an MRI and DEXA scan as described below, so that later, we can evaluate the effects anti-HIV therapy had on your body.

Evaluating how anti-HIV drugs may affect your fat and bone:
Anti-HIV medication (ART) is associated with changes in the fat in your body and loss of minerals from your bones (making bones easier to break). You could also have problems regulating the sugars in your blood (diabetes), or and have higher blood pressure or higher fats in your blood, all of which are bad for your heart and your circulation. To see if these changes are happening to you, we will do whole body tests. These tests are called Magnetic Resonance Imaging (**MRI**) and **DEXA scan**, and are used to measure total fat distribution and bone mineral density. Both procedures expose your body to a very small amount of radiation, and it is safe for you to have such tests over 2.5 years.

What will happen to me during an MRI?
MRI analysis will be done at baseline and study completion. Your study doctor will make an appointment and we will provide transport to and from the Rosebank Clinic where the MRI is done with a special machine (a similar procedure to having an X-ray) that analyze the internal organs and structures of the body **without exposing you to any radiation**. This is an external examination only, and should not cause any harm to you. For the test you will be asked to lay on your back on a bed. This will slide into a special chamber similar to a tube, and you will be required to hold your breath for a few seconds several times during the exam. You will be able to talk with the operator of the machine during the entire scan, even if you cannot see him/her, using a microphone. The test is painless, but you will hear some loud noises. To protect your ears, you will be given special ear plugs that do not block out the operator's voice. The test will last approximately 15 minutes, and can be interrupted at any time if you feel any discomfort and/or fear.. The data (photos) that we collect from this test will help us see if your drugs (ART) have caused changes in fat over time. Possible disadvantages and risks of MRI scans are discussed in detail below. Before the testing, more detailed information as to how to prepare for this test will be handed to you in a letter.

Dual Energy X-Ray Absorptiometry (DEXA)
DEXA analysis will be done at baseline analysis only. At the same time that you have your first MRI, you will be asked to do a DEXA scan, which allows us to evaluate your bone density. This is necessary because the MRI cannot measure your bone density. The DEXA scan is similar to an X-ray, and causes your body to absorb a small dose of radiation. Radiologists consider safe to have one DEXA scan in 2.5 years.

What will happen to me during a DEXA?

The test is safe, painless and takes more or less 15 minutes. As with the MRI, you will be asked to lie on your back on a table without moving while a special probe passes over an area of your body without touching you. A beam of very low intensity x-ray passes through that area of your body, and will not feel anything during the exam. The scan is repeated for different parts of your body. Possible complications and adverse events to DEXA scans are discussed in detail below

Evaluating your risk for cardiovascular disease (blood tests)
As part of this study, we will evaluate your risk of developing cardiovascular disease (heart attacks, strokes and chest angina) by testing your blood as part of your clinical works, and this does not require any more blood drawing.

Drug Initiation Visit and Initial Treatment Period: If you qualify for the study, you will be given all the HIV medicines (ART) and will be taught how to take them. You will initially need to take these medicines for an period of 36 weeks or more, and in that time you should show a response. We will see if you respond by looking at your viral load (HIV in the blood) which must remain at a very low level (less than 50 copies/ml) during the first 24 weeks or 6 months. During this period, if your viral load is >400 copies/ml on more than two visits you cannot continue the study, but we will keep giving you the anti-HIV drugs. For you to continue on the study at the end of the 6 months you need to have both a $CD4^+$ count greater than 450 and a viral load under 50 copies/ml. Should you not achieve suppression and a CD4 count above 450 an additional 8 weeks of follow-up will be allowed. If that added time did not allow for randomization to occur, you will be removed from the study and your study doctor will discuss your continued care and treatment through the National Roll-Out Plan for Antiretroviral therapy. If your virus is controlled but your CD4 count is less than 450, your same drugs will continue but your direct follow-up may be coordinated through the National Roll-Out Plan for Antiretroviral therapy

Initial Rabies Vaccination: While you are taking your antiretroviral therapy, you will be given a complete vaccination against rabies which requires three injections of the vaccine (Verorab®). The purpose of this vaccination is to measure how well your immune system responds. This vaccination is given by 3 injections into the muscle of the upper arm. An additional dose of the Rabies Vaccine or Booster will be given to you at the end of the study period to determine how well your immune system can “remember” the first vaccination based on how well it responds to this when measured 4 weeks later. Possible complications and adverse events to the Rabies vaccination are discussed in detail below.

Study Randomization: All participants for whom the HIV medicines worked well (have suppressed the virus to under 50 copies/ml and a CD4 count above 450) will be assigned to either continue their treatment **OR** interrupt their treatment according to a schedule listed below. This decision is not made by the study personnel, but is made using a process similar to flipping a coin, called **randomization**. There will be a 50:50 chance of you entering one or the other arm of the study.

Continuous Treatment Study Arm: In this arm of the study you will be asked to come to the clinic at regular times for blood testing to check the safety of the treatment and whether it is effective for you. These visits will happen approximately every 8 weeks until week 92 where you will be given a final rabies vaccine (booster) and evaluated 4 weeks later. Pregnancy tests will be performed on all female participants at each visit. Blood testing will be performed at each visit (see below). To measure the effect of treatment on your body fat distribution and bone mineral density, whole body imaging will be performed at the end of the study to compare with the same type of information collected at the start.

Treatment failure in this arm is defined as toxicity to the medication (drugs make you sick) or viral load failure (>5000 c/ml: drugs do not work properly for your virus) on multiple viral loads measurements or CD4 count below 350 cells/mm^3 (your immune system does not improve sufficiently).
Should your treatment under this program fail, you will be discontinued from this treatment regimen and referred for evaluation and antiretroviral therapy through alternative programs of the National Roll-Out Plan for Antiretroviral therapy. Discontinuation of study drug will not affect your study visits unless you indicate otherwise.

Treatment Interruption Arm: In this arm of the study you will be asked to come to the clinic after 4 weeks to interrupt your treatment. Three such interruptions have been planned, each followed by a fixed period of 16 weeks on continuous therapy. The first two interruption periods are shorter than the last one in order to monitor you closely before longer therapy interruptions. Before each interruption you will need to show a minimum of 400 CD4 cells/mm^3 and a viral load <5,000 copies/ml. If your treatment fails (CD4 count does not rise above 400 or goes below entry levels, or your viral load is consistently above 5,000 copies/ml), you will be monitored for a maximum of 12 weeks to allow you met criteria before discontinued from this treatment regimen and study and evaluated for antiretroviral therapy through alternative programs of the National Roll-Out Plan for Antiretroviral therapy. Discontinuation of study drug will not affect your study visits unless you indicate otherwise.

The following summarizes the interruption schedule:

Stop treatment	2 weeks
Resume treatment	16 weeks
Stop treatment	4 weeks
Resume treatment	16 weeks
Stop treatment	8 weeks
Resume treatment	16 weeks
End	4 weeks

The investigator will do tests on your blood to determine if the interruptions are harmful to you. These test will be blood tests of the liver, full blood count, glucose, blood fat testing and kidney function at the beginning and end of each therapy interruption, and monitoring CD4+ count and HIV viral load at these times and 3 months into each re-treatment period to improve your safety during the treatment and interruption periods of the study. Pregnancy tests will be performed on all female participants at each visit.

Blood testing and storage
Regardless of which group you are assigned, we will monitor your blood for the following: 1) the amount of virus in your blood (viral load), 2) your CD4+ T-cell count (a measure of how your immune system has been effected by HIV), 3) resistance of the HIV virus in your blood to ART medications, 4) toxic effects of the medications, and 5) how your immune system responds to particles of HIV in the laboratory. A minimum of 6 ml of blood (less than one tablespoon) will be collected at every visit (2ml for CD4 count, 2 ml for CBC, 2 ml for viral load) with a maximum of 96 ml (less than 5 table spoons) of blood to be collected as specified (2ml for CD4 count, 2 ml for CBC, 2 ml for viral load, 10 ml for metabolic tests, and 64 ml for research).
The results of the clinical tests will be communicated to your doctor, who may use them to better follow how you respond to your treatment.
The amount of blood withdrawn in any 3-month period will be 184 mls, in accordance with Red Cross blood donation guidelines. Please notice that part of the blood will be preserved and then shipped to the Wistar Institute in Philadelphia, USA, where investigators will do more testing.

With this consent, you will be asked to give extra blood (maximum of 10mls) for HIV-related research. You may decline to provide this extra blood and still allow you to participate on this protocol. Even if you do agree to provide this blood, you can withdraw consent at any time without jeopardizing your care. This blood will be stored with protection of your identity and may be used for future research at The University of Witwatersand, Wistar Institute or affiliated investigators.

ELIGIBILITY CRITERIA

To ensure the safe conduct of this study, and to ensure that we can compare the results of the two study arms, a list of inclusion and exclusion criteria have been established. These are listed below and will be discussed in more detail by your study doctor.

Inclusion and Exclusion Criteria (Do I qualify for this study?)

To participate in the study you must be 18 years or older , willing and able to sign this informed consent as an adult. If you need help reading this consent form, or if you are unable to write, you should have a friend or family member with you to witness that you have been informed of all the details in this form.

You must have a CD4 count between 200 cells/mm^3 and 350 cells/mm^3 to be included in the study. The doctors will interpret these results and explain them to you. You should not have had antiretroviral medication in the past.

Your study doctor will check your treatment history to look for medicines that are not allowed on the study and with your HIV treatment. Some may be stopped with alternative treatments suggested, others may prevent you from starting the study. Please check with your study doctor before starting any new medicine to ensure that they do not interfere with the HIV treatment.

The use of alcohol and other drugs is not restricted in this study. However, it is recommended that you do not consume more than 2 beers, or 2 glasses of wine (or equivalent) daily while you are on this study. If you have already received a vaccination against rabies or have a positive test for a hepatitis virus at screening you will not be able to participate in this study.

Pregnancy and contraception

Both male and female participants can participate in this study; however, because it is not known what effect intermittent therapy might have on the developing fetus, women that are pregnant or currently breastfeeding are excluded from the study. If you are a female participant you must have a negative pregnancy test (βHCG) within 72 hours of treatment initiation visit AND you must agree to a pregnancy test at each visit and use appropriate contraceptive methods while on study. Should you become pregnant during the study, you may be discontinued from your study arm and will be offered a pregnancy consent form with the option of continuing treatment throughout pregnancy and continued monitoring for the study duration.

All participants will receive further counseling on the importance of using barrier contraceptive (condoms) to avoid pregnancy and should continue after stopping study drugs . When you are on medication for HIV, including the medication used during this trial you are at risk of giving HIV to your partner. Condoms are proven to reduce the risk of transmission of HIV to a sexual partner and should always be worn. In addition, men should use condoms to prevent their partners from becoming pregnant. Condoms are available from the clinic free of charge. In South Africa we recommend male or female condoms. Other medically proven birth control methods allowed for this study include Depo Provera®, and the intrauterine contraceptive device. However, these methods do not reduce the risk of infecting your partner.You should discuss your preferred

prevention method with your doctor to ensure that you are safely preventing pregnancy throughout the study.

If you or your wife/partner/girlfriend become pregnant or suspect a pregnancy while in the study, you must notify your study doctor immediately.

Medicines other than those supplied by the study site:
All the medicines supplied by your doctors, pharmacists or traditional healers must be shown to your doctor. There are medicines which you cannot take on this study, because they could react with the HIV treatment and cause treatment failure. Please call the 24 hour on call telephone number if you have any questions regarding other medicines you may wish to take. Please check with your study doctor before starting any new medicine to make sure that they do not interfere with the HIV treatment.

You will be provided with a safety card listing the following prohibited medications and the contact details of the doctors at the research clinic. Please keep this with you at all times and show it to any doctor you need to see while on the study.
The prohibited medications include:

- Sedative/Hypnotics such as: midazolam (Dormicum®) or triazolam (Halcion®)
- Antimicrobials used for the treatment of TB such as: rifampin (Rifadin®, Rifcin®, Myrin®, Pyrifrin®, Rifafour®, Rifamate® or Rifater®)
- Neuroleptics such as: pimozide (Opra®)
- Antinauseants/Antidiarrheals such as: cisapride (Propulsid®)
- Antiretrovirals such as: ritonavir (Norvir®) or delavirdine (Rescriptor®)

STUDY ETHICAL APPROVAL:
This clinical trial has been approved by the **University of the Witwatersrand, Human Research Ethics Committee: (Medical-**the body overseeing research at this institution). The study has been designed in accordance with the World Medical Association's Declaration of Helsinki (last updated: October 1996) that deals with the recommendations guiding doctors in biomedical research involving human subjects. A copy of this document may be obtained from the Investigator should you wish to review it. Should you have any complaints to make about the conduct of the study please contact the chairperson of the committee, Prof. Cleaton-Jones at (Tel) 011 717 2229

YOUR RIGHTS AS A PARTICIPANT IN THIS TRIAL
- Your participation in this trial is entirely voluntary and you can refuse to participate or stop your participation at any time without stating a reason.
- If you decide to stop being in this study, this will not affect your access to other medical care in the clinic where your progress is now being followed.
- If you decide to stop being in this study, we offer you to continue your monitoring visits until the end of the study visits in our clinic.

ALTERNATIVE TREATMENT
An alternative to participating in this study is simply to remain on your current treatment and continue to be followed by your doctor over time. The National ARV Roll-out Plan being developed by the Department of Health will provide individuals with antiretroviral therapy if their CD4+ count falls below 200 cell/mm^3. The interrupted therapy proposed in this study however, is experimental and part of a research investigation.

DISCOMFORTS AND RISKS

Research Related Medications
The major risks of the study are associated with antiretroviral therapy (ART). You should understand that you are being asked to take antiretroviral agents which in many cases have well-documented side effects associated with their use as listed below. Since the interaction of the study medications with other drugs is unknown, you should take other drugs only after informing the study doctor. Experimental therapies are not permitted during the study without the approval of the Principal Investigator. Your study team can describe the side effects associated with some of the common medications you may have to take as part of this study. If other drugs are necessary during the course of the study, the side effects will be explained to you before you begin taking them.

HIV related risks – CD4+ count decrease and Drug Resistance
Current treatment guidelines recommend that treatment should be continued for life. The main reasons for this recommendation are to ensure that the HIV virus does not change and become resistant to treatment, and to maintain the CD4+ count as high as possible. The risk of this study include the development of resistance (HIV is not controlled by some drugs) and/or a decrease of the CD4+ count both on treatment and off treatment. The schedule of visits during the study has been designed to monitor carefully the safety of the regimen. If you develop resistance to the drugs provided in the study, you should understand that some other drugs included the National Rollout Plan may also not work. If you develop resistance, the investigators will work with the National Roll-out site to design a treatment combination based on your resistance results.

We have chosen a treatment combination, which is very potent (effective) and has a low risk of mutations. If you choose to participate in this study, you will be monitored to see if the virus present in your blood has mutations that might result in resistance to ART. In case such mutation is found in your blood, and your virus stops responding to the medications you are taking as defined above, you will be notified and referred for evaluation for antiretroviral therapy in the National Roll-Out Plan for Antiretroviral therapy. Discontinuation of study drug will not affect your study visits unless you indicate otherwise.

ART side-effects

The drugs used in this study may have side effects, some of which are listed below. Please note that these lists do not include all the side effects seen with these drugs. These lists include the more serious or common side effects with a known, or possible relationship. If you have questions concerning the additional study drug side effects please ask the medical staff at the site.

Use of Combination Antiretroviral Drugs

The use of potent antiretroviral drug combinations may be associated with an abnormal placement of body fat and wasting. Some of the body changes include:
- Increase in fat around the waist and stomach area
- Increase in fat on the back of the neck
- Thinning of the face, legs, and arms
- Breast enlargement

The drugs you will be given are called: stavudine (also known as d4T or Zerit), lamivudine (also known as 3TC or Epivir) and a combination of lopinavir/ritonavir You may be switched to Zidovidine (Retrovir, AZT) on the study if you can not tolerate or at risk for side –effects of Stavudine.

Stavudine, Retrovir, and **lamivudine** belongs to a family of drugs called nucleoside reverse transcriptase inhibitors (NRTI). Lactic acidosis and severe hepatomegaly (enlarged liver) with steatosis (fatty liver) that may result in liver failure, other complications and death have been reported with the use of antiretroviral nucleoside analogues alone or in combination. The liver complications and death have been seen more often in women on these drug regimens. Some nonspecific symptoms that might indicate lactic acidosis include: unexplained weight loss, stomach discomfort, nausea, vomiting, fatigue, weakness, and shortness of breath.

The following side effects have been associated with the use of **stavudine**:

- Deaths from liver failure have been reported in pregnant women receiving the combination of stavudine and didanosine with other anti-HIV drugs.
- People who take stavudine together with didanosine, with or without hydroxyurea, may be at greater risk for pancreatitis or liver problems or both. These conditions may result in death.
- Numbness, tingling, and pain in your hands or feet
- Pancreatitis (inflammation of the pancreas), which may cause death. If you develop pancreatitis, you may have one or more of the following: stomach pain, nausea, and vomiting.
- Rash
- Dizziness, muscle pain, cramps (nonspecific lactic acidosis symptoms)
- Upset stomach, vomiting and loose or watery stools
- Abdominal pain
- Abnormal liver function blood tests or abnormal pancreatic function blood tests
- Rare cases of muscle weakness, which may progress to paralysis and inability to breathe (This may be associated with elevation of lactic acid in the blood).

When stavudine is used with other medicines with similar side effects, these side effects may be seen more often, and may be more severe, than when stavudine is used alone.

The following side effects have also been associated with use of **lamivudine**:

- Headache
- Feeling of vague overall discomfort
- Feeling tired
- Dizziness
- Depression
- Upset stomach
- Vomiting

- Loose or watery stools
- Decrease in appetite
- Abdominal cramps
- Sleeplessness
- Rash
- Numbness, tingling, and pain in the hands or feet
- Decrease in the number of white blood cells that help fight infection
- An increase in a substance in the blood (a type of a pancreatic enzyme) which could mean a problem with the pancreas
- Increased liver function tests, which could mean liver damage
- If you are infected with both Hepatitis B and HIV, you should be aware that your liver function tests may increase, and symptoms associated with hepatitis (an acute inflammation of the liver) may worsen if lamivudine is stopped. Although most of these cases have resolved without treatment, some deaths have been reported.
- Pancreatitis (inflammation of the pancreas), which may cause death. If you develop pancreatitis, you may have one or more of the following: stomach pain, nausea, and vomiting.

The following side effects can occur with **zidovudine**;

- Headache
- Fatigue
- wasting
- Upset stomach
- Decrease in appetite, vague overall feeling of discomfort, and heartburn
- Insomnia
- Nausea/vomiting
- Finger nail discoloration
- Leg muscle weakness
- Numbness, tingling, and pain in your hands or feet
- Decrease in the number of white blood cells that help fight infection
- Decrease the number of red blood cells in your body that carry oxygen to your body
- Rare cases of muscle weakness, which may progress to paralysis and inability to breathe (This may be associated with elevation of lactic acid in the blood).

Lopinavir and **ritonavir** belong to a class of drugs called protease inhibitors: the use of these drugs may be associated with the following:

- Increases in the amount of triglycerides and/or cholesterol in the blood
- Development of diabetes or the worsening of high blood sugar.

There have been reports of increased bleeding in HIV-infected persons with hemophilia who were treated with protease inhibitors. It is not known if protease inhibitors were the cause of these bleeding episodes.

The following side effects are also associated with the use of **lopinavir/ritonavir:**

- Pancreatitis (inflammation of the pancreas), which may cause death. If you develop pancreatitis, you may have one or more of the following: stomach pain, nausea, vomiting or abnormal pancreatic function blood tests
- Abnormal bowel movements (stools), including loose or watery stools, upset stomach and stomach pain
- Large increases in triglycerides and cholesterol in the blood
- Liver problems and worsening liver disease, which may result in death. People with these conditions may have abnormal liver function blood tests
- Feeling weak and tired
- Headache
- Rash (seen in children)

For your safety, you will be monitored throughout the study for any of the above side effects.

The Rabies vaccine (Verorab®) will be given to you on three occasions in the first 34-week treatment period and once at the next-to-last study visit. You may feel a slight discomfort (pain), and may have redness and slight hardening of the area at the injection site. The vaccination will be used with care in cases of true allergy to streptomycin and/or neomycin as there may be very small amounts of these drugs present in the vaccine. There have also been rare events of fever associated with this vaccination.

Blood Sampling

Blood drawing from a vein may cause some discomfort (pain), and occasionally some bleeding or bruising at the site. On rare occasions, people faint while having blood drawn. There is a small risk of infection. Blood drawing will not exceed 184 mls over a 3 month period, which is within the safety guidelines for blood drawing practiced at the study clinic site.
Part of your blood sample will be shipped to the Wistar Institute in Philadelphia, USA for testing of inflammatory markers and other parameters as part of the evaluation of your immune system.

Possible disadvantages and risks of MRI scans

This is a relatively safe procedure but contraindicated in persons with permanent metal devises or history of psychiatric depression or severe fear of confined spaces as specified below. However, if you suffer from the following conditions, we strongly recommend that you inform us, as these will exclude you from this test:

- previous brain surgery with metal clips
- a heart pace maker
- psychiatric depression or any form of claustrophobia
- bilateral hip replacements or other bone and joint procedures
- spinal surgery
- uncontrolled/ untreated epilepsy

Possible disadvantages and risks of DEXA scans

DEXA scans use low energy X-rays, and are considered very safe. However, you should not have this test if you are pregnant or think you might be pregnant.

Can I give HIV to my partner while on this study?
We do not know if transmission can be stopped even if taking therapy (ART). There will be a greater risk for transmission in participants in the ART interruption group while not taking the therapy. If you develop drug resistance your partner, if infected, may get a resistant virus.

COSTS AND BENEFITS
The benefits of the study are that all your blood tests, study drugs and visits will be at no cost to you. There is no direct compensation for participation in this study. Antiretroviral drugs allow HIV infected people to live longer. Also, your health will be followed more closely than usual while you are on the study, which may help you feel better. The study site will compensate you for transport and food for your scheduled clinic visit at. R150.00 per visit

DISCONTINUATION OF THE TRIAL TREATMENT
You may voluntarily withdraw from this study at any time. You will be discontinued from the study and followed-up) if you develop severe side effects, therapy has failed to suppress HIV-1 or increase your CD4 T cell count, a malignancy other than Kaposi's sarcoma, or a life-threatening infection. In addition, you may have to leave the study if it is felt by the Principal Investigator to be in your best medical interest, if you permanently discontinue antiretroviral therapy for any reason, if you do not comply with the study rules, or if the study ends.

NEW FINDINGS
The investigators will tell you of any significant new finding that might affect your willingness to continue participating in this study. You should feel free to discuss these findings with them, and to decide whether or not you want to continue in the study.

SAFETY STUDIES
In recent months two Structured Interrupted studies have discontinued the interruption arm due to more complication in the STI arm. Both studies are different than this study in that theses studies allowed patients who had CD4 counts below 200 on the trial and patients who had other diseases from their HIV. We will use a different method to determine when treatment interruptions occur, and will monitor you closely to see if you develop any complications; however, there is no proof that our method is safer than the methods used in previous studies.

POST-STUDY DRUG ACCESS
Continued access to anti-HIV drugs (ART) will be through the National ART Roll-out plan also administered at the study site.

INSURANCE AND FINANCIAL ARRANGEMENTS

This clinical study is sponsored by the USA National Institutes of Health, The Wistar Institute and The Wits Health Consortium who will support all trial procedures. Reasonable medical expenses that may be incurred as a direct result of this trial will be provided as determined by Dr Sanne and the Investigators.
If you are injured as a result of these research drugs or procedures, medical care will be provided to you. **Neither you nor your medical aid will be expected to pay for these procedures.** You will not be provided with money if you are injured for other reasons or develop medical or surgical problems not related to the study. This treatment will be provided to you in the Helen Joseph Hospital by the Department of Health. There is no program for compensation either through this institution or the National Institutes of Health (NIH). Should the Helen Joseph Hospital not be able to provide you with the appropriate care for your medical situation, an insurance policy has been taken out for all participants in the study. This policy will only pay for the medical needs of someone who is injured during the study, and the injury is directly related to the study.

SOURCES OF ADDITIONAL INFORMATION
For the duration of the trial, you will be under the care of **………………………..** If at any time between your visits you feel that any of your symptoms are causing you any problems, or you have any questions during the trial, please do not hesitate to contact him/her. You may ask any questions without this affecting your future medical care.
The contact details of your doctors at the clinic include the following:

Office telephone number 011276 88 00
Pager for doctor on call 012 333 6000 code 8398

If you have additional questions regarding your rights as a research subject, you may discuss them with a member of the **University of the Witwatersrand, Human Research Ethics Committee: (Medical)** (our local ethics committee) at telephone number 011 717 2229 (Prof. Cleaton-Jones) or the Medical Control Council at:
The registrar of Medicines
Department of Health
Private Bag X828
Pretoria
0001

CONFIDENTIALITY
All information obtained during the course of this trial is strictly confidential. Data that may be reported in scientific journals will not include any information, which identifies you as a participant in this trial. All records are maintained by the clinical research site under the supervision of Dr Sanne, and all records or stored blood samples outside of the clinic provided to USA coinvestigators (The Wistar Institute, University of Massachusetts, University of Pennsylvania) will only identify you by your study initials and study number. Information identifying you will only be available to the site personnel.

In connection with this trial, it might be important for domestic and/or foreign health authorities, the **University of the Witwatersrand, Human Research Ethics**

Committee: (Medical) as well as your personal doctor, to be able to review your medical records pertaining to this trial. Therefore you hereby authorize your investigator to release your medical records to the National Institutes of Health, University of the Witwatersrand and The Wistar Institute, its employees or agents, domestic and/or foreign health authorities (i.e. Medicines Control Council, etc.) and the **University of the Witwatersrand, Human Research Ethics Committee: (Medical)**. You understand that these records will be utilized by them only in connection with carrying out their obligations relating to the clinical trial. You will not be identified by your name but only by your study number.

INFORMED CONSENT

I have been informed by the investigator, Dr. ………………… about the nature, conduct, benefits and the risks of this clinical trial 'A Randomized Clinical Trial Assessing Continuous HAART versus Interrupted HAART in a Resource Poor Clinic**. I have also received, read and understood the written information (Participant Information Leaflet and Consent form) regarding the clinical trial.**

I am aware that the results of the trial, including personal details regarding my sex, age, date of birth, initials and diagnosis will be anonymously processed into the trial report.

I may, at any stage, without prejudice, withdraw my consent and participation in the trial.

I have had sufficient opportunity to ask questions and (of my own free will) declare myself prepared to participate in this trial.

Participant's Name: ______________________________
(please print)

Participant's Signature: ______________________________ **Date/Time** ________________________

I agree ____ or disagree_____ to the collection of an additional 10cc for HIV-1 related Research.

Participant's Signature: ______________________________ **Date/Time** ________________________

I, ___________________________ (please print) hereby confirm that the above participant has been informed fully about the, nature, conduct and risks of the above trial.

Person Obtaining Consent Signature: ______________________________ **Date/Time** ________________________

Witness' Name: (If applicable) ______________________________
(please print)

Witness' Signature: (If applicable) ____________________________ **Date/Time**

Social Worker Name:

Signature ______________________________ **Date/Time** ______________________________

Initial Study Period Diagram

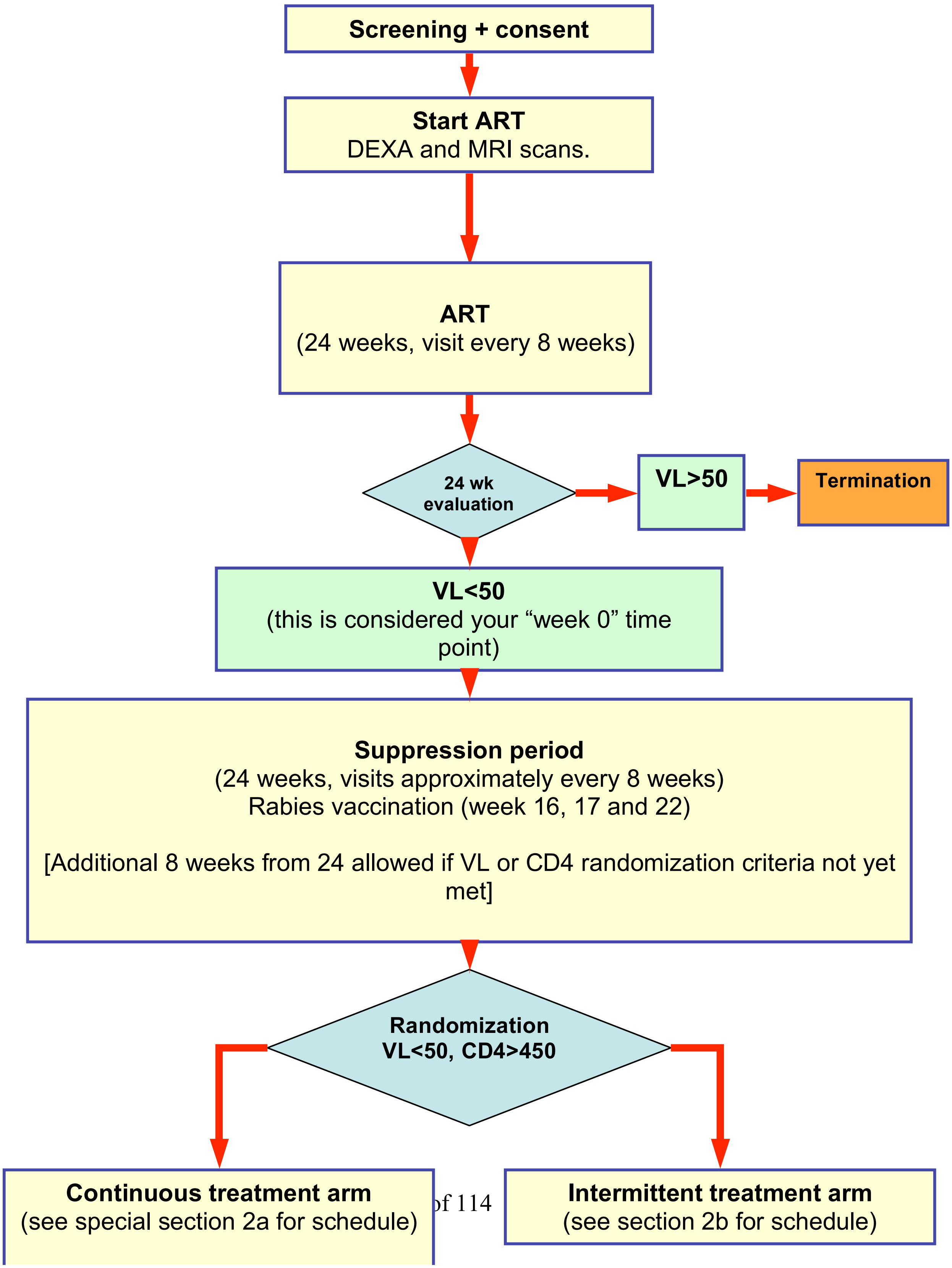

Continuous Arm Diagram

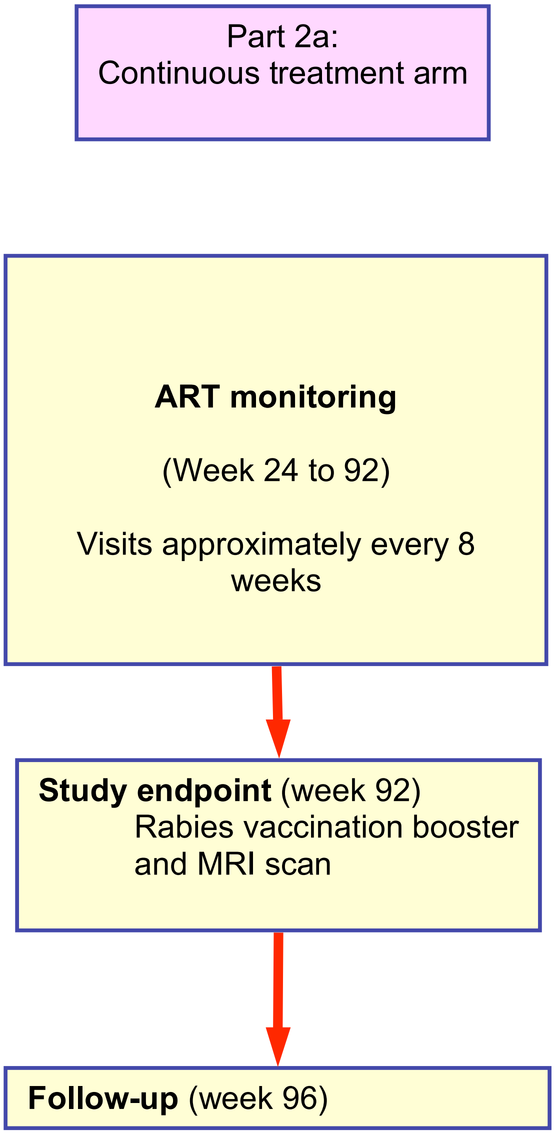

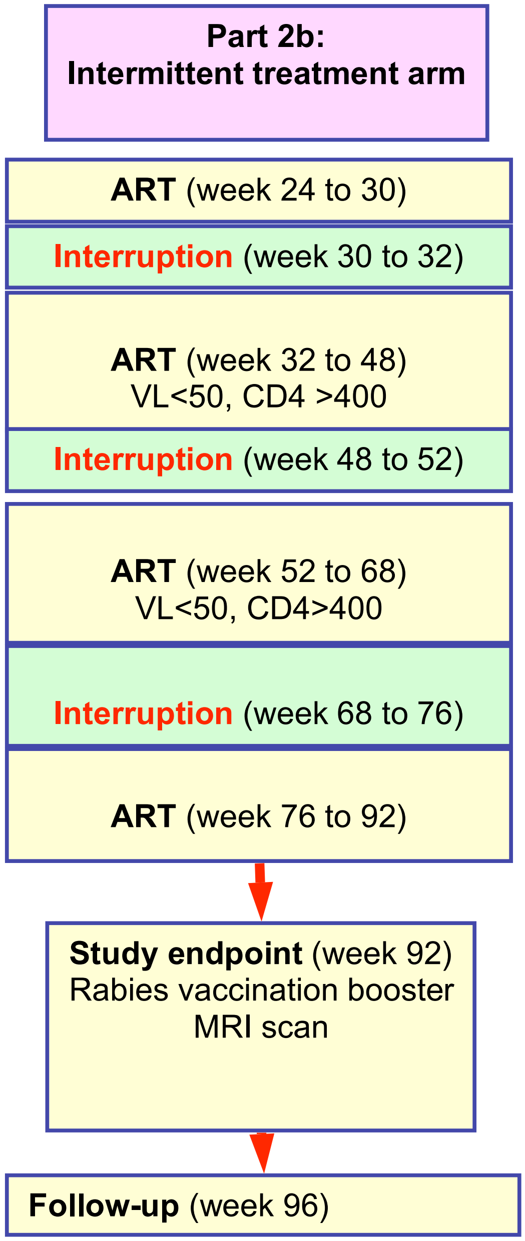
Part 2b:
Intermittent treatment arm
ART (week 24 to 30)
Interruption (week 30 to 32)
ART (week 32 to 48)
VL<50, CD4 >400
Interruption (week 48 to 52)
ART (week 52 to 68)
VL<50, CD4>400
Interruption (week 68 to 76)
ART (week 76 to 92)
Study endpoint (week 92)
Rabies vaccination booster
MRI scan
Follow-up (week 96)

XV. PREGNANCY INFORMED CONSENT

Division Of Aids Research
National Institute of Health,(NIH) USA

Women Who Become Pregnant While On Study

TRIAL TITLE:
'A Randomised Clinical Trial Assessing Continuous HAART versus Interrupted HAART in a Resource Poor Clinic' (RO1 AI 51986-01)

PROTOCOL NUMBER:
RO1 AI 51986-01

INTRODUCTION

Because you are now pregnant, you are being asked if you want to continue taking part in this research study. The main study is comparing "treatment interruption and continuous HAART in a Resource Poor Clinic". This study was designed so that women who are pregnant before the randomization visit will not be part of the study. If you are pregnant on the study, then you are now given options for further treatment.

WHAT ARE MY ANTI-HIV DRUG TREATMENT OPTIONS DURING PREGNANCY?
Treatment with anti-HIV drugs during pregnancy is recommended to reduce the risk of HIV transmission to infants. The safety of the medicines you are taking has not been fully established. There is however no known risk to babies born of mothers taking the treatment you are taking. Your doctor is able to explain this in more detail to you.

You have three options for treatment during your pregnancy:

1. To continue taking the treatment regimen throughout the pregnancy, and be monitored at the study site.
2. To stop your study treatment for the first three months of the pregnancy, restart in the second three months, and be monitored at the study site.
3. To discontinue my study participation and anti-HIV medicines and seek treatment provided by the Department of Health at a later date upon meeting therapy guidelines. Should you not wish to be followed at the research clinic then you will need to receive your treatment at one of the Department of Health clinics.

If you are pregnant during the period before randomization, you may decide to continue treatment for the remaining months of study. The study will provide treatment to you for the duration of your pregnancy, however you will not be included in the study. When your baby is born you will be referred to the HIV clinic of the Helen Joseph Hospital for further care.

WHAT SHOULD I DO IF I AM IN THE INTERUPTING ARM OF THE STUDY
It is not safe for you to be part of the study arm which is interrupting treatment while you are pregnant as the increases in viral load may lead to your baby becoming HIV positive. You can therefore no longer be in the in interrupting arm of the study. You may continue taking the treatment and be monitored at the site.

WHAT SHOULD I DO IF I AM IN THE CONTINUOUS TREATMENT ARM?
If you are in the continuous treatment arm of the study you may be continued in the study on your study treatment

WHAT DO I HAVE TO DO IF I STAY IN THIS STUDY?

If you decide to continue to participate in this study while you are pregnant, you will continue to come to the clinic for study visits as described below.

This study will not provide care related to your pregnancy, the delivery of your baby or the care of your baby. You must arrange for your care and your baby's care outside of this study.

Long-term follow-up is recommended for a baby whose mother is HIV-positive during pregnancy. The study staff will talk with you about long-term follow-up and the possibility of enrolling your baby in a long-term follow-up study.

You will come to the clinic for the scheduled study evaluations described in the consent form you signed earlier until you advice otherwise.

WHAT ARE THE RISKS RELATED TO STAYING IN THE STUDY?

If you have questions concerning any additional study drug side effects, now that you are pregnant, please ask the medical staff at your site. Being pregnant may increase the likelihood of toxicities to drugs.

ARE THERE BENEFITS TO STAYING IN THIS STUDY?

Apart from benefits already stated in study consent, if you elect to continue your medication and it maintains viral replication down, the risks for transmission of HIV to your baby are decreased at the time of delivery.

BREAST-FEEDING

After your pregnancy, you may not re-start taking study drugs, but you will be asked to continue study visits. It is important for you to know that HIV can pass through breast-milk and taking anti-HIV drug(s) has not been proven to decrease the chance of passing HIV through your breast-milk to your baby. You should discuss this with your doctor.

WHAT ABOUT CONFIDENTIALITY?

We will do everything we can to protect your privacy.. Also, any publication of this study will not use your name or identify you personally.

People who may review your records include: the Ethics Committee (EC), namely University of the Witwatersrand, Human Research Ethics Committee Medical, Wistar Institute Ethics Committee, National Institutes of Health (NIH), study staff, and study monitors.

WHAT ARE THE COSTS TO ME?

In addition to any costs that are described in the study consent you already signed; this study will not cover any cost related to your pregnancy, delivery of your baby or care of your baby.

WHAT HAPPENS IF MY BABY OR I IS/AM INJURED?

If your baby or you are injured as a result of these research drugs or procedures, you will both be given immediate medical care for your injuries. Neither you nor your medical aid will be expected to pay for these procedures. You will not be provided with money if you are injured for other reasons or develop medical or surgical problems not related to the study. This treatment will be provided to you in the Helen Joseph Hospital by the Department of Health. There is no program for compensation either through this institution or the National Institutes of Health (NIH). You will not be giving up any of your legal rights by signing this consent form.

WHAT ARE MY RIGHTS AS A RESEARCH SUBJECT?

Continuing to take part in this study is completely voluntary. You may choose not to continue in this study or leave this study at any time. You will be treated the same no matter what you decide.

We will tell you about new information from this or other studies that may affect your health, welfare or willingness to stay in this study. If you want the results of the study, let the study staff know.

WHAT DO I DO IF I HAVE QUESTIONS OR PROBLEMS?

For questions about this study or a research-related injury, contact:

- Emergency pager: (012) 333-6000 code 8398
- Tel: (011) 276 8800

If you want any further information regarding your rights as a research participant, or complaints regarding this research study, you may contact Prof Cleaton-Jones, Chairperson of the University of the Witwatersrand , Human Research Ethics Committee (HREC), which is an independent Committee established to help protect the rights of research Participants at 011 717-2229.

You may also contact the Medicines Control Council:
The Registrar of Medicines, Department of Health, Private Bag X828, PRETORIA, 0001

SIGNATURE PAGE

If you have read this consent form (or had it explained to you), all your questions have been answered and you agree to continue to take part in this study, please sign your name below.

______________________________	______________________________
Participant's Name (print)	Participant's Signature, Date and Time
______________________________	______________________________
Participant's Legal Guardian (print)<br>(As appropriate)	Legal Guardian's Signature, Date and Time
______________________________	______________________________
Study Staff Conducting<br>Consent Discussion (print)	Study Staff Signature, Date and Time
______________________________	______________________________
Witness's Name (print)	Witness's Signature, Date and Time

XVIII. Appendix B:

Manual for Expedited Reporting of Adverse Events to DAIDS.

Final May 6, 2004

Appendix B: Table of Contents

1.0 PURPOSE OF MANUAL 102
1.1 Purpose 102
1.2 Scope 102
1.3 Introduction 102
2.0 DESCRIBING AN ADVERSE EVENT BY SERIOUSNESS, SEVERITY, RELATIONSHIP TO STUDY AGENT, AND EXPECTEDNESS 103
2.1 Seriousness 103
2.2 Severity (Intensity) 103
2.3 Seriousness vs. Severity (Intensity) of Adverse Events and Reporting Criteria 103
2.4 Relationship to Study Agent 104
2.5 Expectedness (Expected vs. Unexpected) 104
3.0 ADVERSE EVENTS REQUIRING EXPEDITED REPORTING AND THE STUDY/TRIAL REPORTING PERIOD 105
3.1 Levels of Adverse Event Reporting 105
3.2 Additional Protocol-Required Expedited Reporting Requirements 106
3.3 Additional Adverse Events That Should Be Reported for Any Study/Trial Requiring Expedited Reporting to DAIDS 106
3.4 Protocol-Defined Expedited Adverse Event Reporting Period 107
4.0 METHOD AND TIMEFRAME FOR EXPEDITED REPORTING OF INDIVIDUAL ADVERSE EVENTS 108
5.0 ADDITIONAL EXPEDITED REPORTING REQUIREMENTS 109
5.1 Follow-up Reporting of Adverse Events 109
5.2 Reporting Recurrent Adverse Events 109
5.3 Reporting Change in Severity of Adverse Events 109
5.4 Study Physician Assessment and Signature 110
6.0 APPENDICES 111
6.1 Appendix A: Definition of Terms 111
6.2 Appendix B: Contact Information for DAIDS Safety Office 113
6.3 Appendix C: Summary Chart for Expedited Reporting of Adverse Events to DAIDS for Protocol-Specified Study Agents 114

Purpose of Manual

1.1 **Purpose**

The purpose of this Manual is to describe the criteria and method for expedited reporting of certain serious and other reportable adverse events to the Division of AIDS (DAIDS), National Institute of Allergy and Infectious Diseases (NIAID), through the DAIDS Safety Office.

1.1 Scope

This Manual applies only to those clinical studies/trials requiring expedited reporting of adverse events to the DAIDS Safety Office as stated in the protocol.

This Manual applies to all study agents specified in the protocol as requiring expedited reporting to DAIDS. Although not covered under this Manual, note that DAIDS may require MedWatch reporting (using e.g., Form FDA 3500A or CIOMS I Form) to the Food and Drug Administration (FDA) and/or DAIDS for some studies. MedWatch reporting may only be applied to studies/trials of US FDA-approved study agents. Any requirements for MedWatch reporting will be identified in the study/trial protocol.

2.1 **Introduction**

For adverse events requiring expedited reporting to DAIDS, sites must follow the general reporting requirements and procedures described in this Manual. In order to fully define the expedited adverse event reporting requirements that apply to an individual study/trial, the protocol will specify:

- One of three Levels of Adverse Event Reporting (Section 3.1) and any other adverse events to be reported on an expedited basis (Section 3.2).
- The duration of the protocol-defined expedited reporting period.
- The name or category of each study agent (US FDA-approved or investigational) that requires expedited reporting of adverse events to DAIDS. This may include study agents in addition to those provided by the study/trial.

Describing an Adverse Event by Seriousness, Severity, Relationship to Study Agent, AND EXPECTEDNESS

The criteria for expedited reporting of adverse events to the DAIDS Safety Office include the seriousness of the outcome of the event, the severity (intensity) of the event, its relationship to study agent, and (only for the Targeted Level) expectedness, i.e., whether the adverse event is expected or unexpected.

3.1 Seriousness

The first consideration for expedited reporting of adverse events to DAIDS is the seriousness of the outcome of the event. The April 1996 International Conference on Harmonisation (ICH) guidance, "Good Clinical Practice: Consolidated Guidance," (ICH E6) defined a serious adverse event (SAE) as "any untoward medical occurrence that at any dose:

- Results in death,
- Is life-threatening,
- Requires inpatient hospitalization or prolongation of existing hospitalization,
- Results in persistent or significant disability/incapacity, or
- Is a congenital anomaly/birth defect."

"Important medical events that may not be immediately life-threatening or result in death or hospitalization but may jeopardize the patient or may require intervention to prevent one of the outcomes listed in the definition above" may also be considered to be serious. (October 1994 ICH guidance (E2A), "Clinical Safety Data Management: Definitions and Standards for Expedited Reporting.")

4.1 Severity (Intensity)

The second consideration for expedited reporting of adverse events to DAIDS is the severity (intensity) of the event. In order to maintain consistency among studies/trials and sites, DAIDS has developed a list of common clinical and laboratory adverse events and defined grade 1 – 5 severity parameters to generate the Division of AIDS Tables for Grading Adult and Pediatric Adverse Experiences (also known as "the toxicity tables"). These tables are located on the DAIDS Safety Office website at http://rcc.tech-res-intl.com.

Unless stated otherwise in the protocol, study staff is required to use the Division of AIDS Tables for Grading Adult and Pediatric Adverse Experiences to determine the intensity of adverse events in order to establish consistency in adverse event reporting to DAIDS. Specific protocols may include additional or modified criteria for grading adverse events that are not included in the current versions of the Division of AIDS Tables for Grading Adult and Pediatric Adverse Experiences.

5.1 Seriousness vs. Severity (Intensity) of Adverse Events and Reporting Criteria

For expedited reporting to DAIDS, the term "severity" (or "intensity") is described as the grade for a specific event, i.e., mild (Grade 1), moderate (Grade 2), severe (Grade 3), or life-threatening (Grade 4). This is *not* the same as "serious," which is based on subject/event *outcome or action* criteria usually associated with events that pose a threat to a subject's life or functioning (ICH E2A).

6.1 Relationship to Study Agent

The third consideration for expedited reporting of adverse events to DAIDS is the judgment of causal association (relationship) between an adverse event and the study agent. The protocol must specify by name or category each study agent (either approved or investigational) that requires expedited reporting of adverse events to DAIDS. The study physician makes the site's final assessment of the causal association based upon the temporal relationship to administration of the study agent(s), the pharmacology of the study agent(s), and his/her clinical judgment.

The terms used in DAIDS studies/trials to assess relationship of an event to study agent are:

- **Definitely Related**. The adverse event and administration of study agent are related in time, and a direct association can be demonstrated.
- **Probably Related**. The adverse event and administration of study agent are reasonably related in time, and the adverse event is more likely explained by study agent than other causes.
- **Possibly Related**. The adverse event and administration of study agent are reasonably related in time, and the adverse event can be explained equally well by causes other than study agent.
- **Probably Not Related**. A potential relationship between study agent and the adverse event could exist (i.e., the possibility cannot be excluded), but the adverse event is most likely explained by causes other than the study agent.
- **Not Related**. The adverse event is clearly explained by another cause not related to the study agent.
- **Pending.** Pending may be used as a temporary relationship assessment only for death and only if data necessary to determine relationship to study agent are being collected. The site is required to submit a final assessment within 3 business days after reporting the death. If no final assessment is made within 3 business days after the date of submission, the event will be assessed as possibly related to study agent. Any additional information received at a later time, including an autopsy report, should be submitted as a Follow-up Report.

A **suspected adverse drug reaction (SADR)** is an adverse event that could potentially have a causal relationship to the study agent (definitely, probably, possibly, probably not related, or for deaths, pending).

7.1 Expectedness (Expected vs. Unexpected)

Expected refers to the perspective of events previously observed, *not* on the basis of what might be anticipated from the pharmacological properties of the study agent. (ICH E2A)

Unexpected refers to events whose nature or severity (intensity) is not consistent with those included in the package insert/summary of study agents that have been approved by the US FDA or in the Investigator's Brochure. (ICH E2A)

Adverse Events Requiring Expedited Reporting And the study/TRIAL Reporting Period

8.1 Levels of Adverse Event Reporting

The protocol will specify one of three Levels of Adverse Event Reporting. The Level of Adverse Event Reporting chosen for expedited reporting is based primarily upon the degree of risk that may be associated with the study agent.

Standard Level

Report all adverse events following any exposure to study agent that:

- Result in death **regardless** of relationship to study agent.
- Are congenital anomalies, birth defects, or fetal losses **regardless** of relationship to study agent.
- Result in persistent or significant disabilities or incapacities **regardless** of relationship to study agent.
- Are a **suspected adverse drug reaction**, i.e., definitely, probably, possibly, and probably not related, to a study agent that requires or prolongs existing hospitalization, or requires intervention to prevent significant/permanent disability or death.
- Are life-threatening (including all Grade 4 adverse events) **suspected adverse drug reactions**, i.e., definitely, probably, possibly, and probably not related to a study agent.

Intensive Level

In addition to all adverse events reported for the Standard Level, also report all Grade 3 suspected adverse drug reactions, i.e., definitely, probably, possibly, and probably not related to a study agent. (The Intensive Level includes reporting Grades 3 and 4 SADRs.)

Targeted Level

Use of the Targeted Level of reporting is limited to non-IND studies/trials of US FDA-approved agents and doses for approved indications and populations. Report **only** the following adverse events:

- All events that result in death **regardless of relationship** to study agent.
- All congenital anomalies, birth defects, or fetal losses **regardless of relationship** to study agent.
- All persistent or significant disability or incapacity **regardless of relationship** to study agent.
- **Unexpected* suspected adverse drug reactions**, i.e., definitely, probably, possibly, and probably not related to a study agent, that require or prolong existing hospitalization, or require intervention to prevent death or significant/permanent disability.
- **Unexpected*** life-threatening <u>clinical</u> **suspected adverse drug reactions**, i.e., definitely, probably, possibly, and probably not related to a study agent. **DO NOT report** Grade 4 laboratory values that are not associated with a life-threatening clinical event.

*Unexpected events are events whose nature or severity is not consistent with the package insert/summary of product characteristics for a US FDA-approved study agent.

9.1 Additional Protocol-Required Expedited Reporting Requirements

In addition to specifying one of the reporting levels above, a protocol may require other adverse events to be reported on an expedited basis. In this case, the protocol will explicitly state the additional adverse events to be reported to DAIDS. For example, in rare instances a protocol may specify use of the Intensive Level and also require Grades 1 and 2 SADRs to be reported, or a protocol may require reporting of a specific type of adverse event regardless of grade.

Additional Adverse Events That Should Be Reported for Any Study/Trial Requiring Expedited Reporting to DAIDS

In addition to the reporting requirements described above, sites should report any of the following adverse events on an expedited basis:

- Suspected adverse drug reactions, i.e., definitely, probably, possibly, and probably not related to a study agent, that **do not meet the protocol-required reporting criteria**, but the Investigator believes are of sufficient concern to be reported on an expedited basis to DAIDS. This includes adverse events that, based upon appropriate medical judgment, may jeopardize the subject and may require medical or surgical intervention to prevent a serious adverse event. Examples of such events are intensive treatment in an emergency room or at home for allergic bronchospasm or blood dyscrasias or convulsions that do not result in hospitalization.

- Unexpected, serious suspected adverse drug reactions, i.e., definitely, probably, possibly, and probably not related to a study agent, that occur at any time **after the protocol-defined expedited reporting period** if the study staff become aware of its occurrence. These events include deaths, permanent disabilities, congenital anomalies, hospitalizations, and life-threatening clinical events. (Do not report Grade 4 laboratory values unless associated with a life-threatening clinical event.)
- Serious adverse events that are not related to a study agent, but could be associated with **study participation or procedure** (e.g., pulmonary embolism secondary to an intravenous catheter placed for study agent administration).

10.1 Protocol-Defined Expedited Adverse Event Reporting Period

The protocol-specified reporting level continues throughout the study/trial period (from enrollment of a subject through the end of study follow-up visits for that subject). The protocol may also require the same level of adverse event reporting to be continued beyond the end of study follow-up for each subject, and if so, the protocol must specify the duration of this additional reporting period.

Method and Timeframe for Expedited Reporting of INDIVIDUAL Adverse Events

All information requested on the DAIDS Expedited Adverse Event Reporting Form must be provided and the form submitted to the DAIDS Safety Office. This form can be found at the web site for the DAIDS Safety Office. Contact information for the DAIDS Safety Office is provided in Appendix B.
The timeframe for expedited reporting of individual adverse events begins when the site recognizes that an event fulfills the criteria outlined in this Manual for expedited reporting to DAIDS. Sites must submit adverse events requiring expedited reporting to the DAIDS Safety Office as soon as possible, **but no later than 3 business days**, after the site's recognition that the event fulfills the criteria for expedited reporting.

Additional Expedited Reporting Requirements

11.1 **Follow-up Reporting of Adverse Events**

Submitting Follow-Up Information on Adverse Events

For the circumstances listed below, the site is required to submit follow-up information when it becomes available on a new Expedited Adverse Event Form as a Follow-up Report.

- Requests by DAIDS for additional information.
- A change in the relationship between the adverse event and study agent by the study physician.
- Additional significant information that becomes available for a previously reported adverse event. This is particularly important for new information addressing cause of death if the initial assignment was "pending."
- Results of rechallenge with the study agent(s), if performed.

Outcome of Adverse Events

The site **must** follow each reported adverse event and record eventual outcomes in the source documentation. However, report of the outcome of a reported adverse event to the DAIDS Safety Office is not required unless specifically requested by DAIDS.

12.1 **Reporting Recurrent Adverse Events**

For events that have been previously reported to the DAIDS Safety Office, if the event has fully resolved and then re-occurs to a level requiring expedited reporting, the adverse event must be reported as a New Report to the DAIDS Safety Office.

13.1 **Reporting Change in Severity of Adverse Events**

Any ongoing event that increases in severity to a higher grade than previously reported must be reported again as a New Report on a new DAIDS Expedited Adverse Event Reporting Form.

Ongoing events that improve, but are not resolved, and then increase in severity to the same or lower severity grade than previously reported do not have to be reported again to the DAIDS Safety Office. Resolution is the normalization or return to baseline (i.e., prior to study agent exposure) of laboratory values, signs, or symptoms related to the event.

14.1 Study Physician Assessment and Signature

A study physician listed on the Form FDA 1572 for IND studies or the DAIDS Investigator of Record Agreement (IoR) for non-IND studies must review and verify the data on the DAIDS Expedited Adverse Event Reporting Form for accuracy and completeness. This physician also makes the site's final assessment of the relationship between the study agent and the adverse event. This physician must sign the completed DAIDS Expedited Adverse Event Reporting Form. If necessary to meet timely reporting requirements, sites can submit an expedited adverse event report without a completed signature page. However, the completed signature page, and necessary corrections or additions, must be submitted within the next 3 business days.

appendices

15.1 EAE Manual Appendix A: Definition of Terms

Adverse Event (AE): An adverse event (AE) is any untoward medical occurrence in a patient or clinical investigation subject administered a pharmaceutical product and which does not necessarily have a casual relationship with this treatment. An AE can therefore be any unfavorable and unintended sign (including an abnormal laboratory finding), symptom, or disease temporally associated with the use of a medicinal (investigational) product, whether or not related to the medicinal (investigational) product. (ICH E6) (Synonym: Adverse Experience)
DAIDS Safety Office: The Office to which adverse events requiring expedited reporting are submitted. (DAIDS)
Division of AIDS Tables for Grading Adult and Pediatric Adverse Experiences (Toxicity Tables): Lists of common terms and severity (intensity) parameters used to describe adverse events occurring in DAIDS-sponsored clinical studies/trials. (DAIDS)
IND: An investigational new drug application. (21 CFR 312.3)

Investigator's Brochure: A compilation of the clinical and nonclinical data on the investigational product(s) that is relevant to the study of the investigational product(s) in human subjects. (ICH E6)

Non-IND Study/Trial: A study/trial for which there is no IND filed with the US FDA.

Package Insert: The approved package circular in marketed drug packaging containing the drug description, clinical pharmacology, indications and usage, contraindications, warnings, precautions, adverse reactions, drug abuse and dependence, dosage and administration, how drug is supplied, "clinical studies," and "references." (21 CFR 201.57)

Serious Adverse Event (SAE): Any untoward medical occurrence that at any dose results in death, is life-threatening, requires inpatient hospitalization or prolongation of existing hospitalization, results in persistent or significant disability/incapacity, or is a congenital anomaly/birth defect. This includes important medical events that may not be immediately life-threatening or result in death or hospitalization but may jeopardize the patient or may require intervention to prevent one of the outcomes listed in the definition above. (ICH E6 and E2A)

Study Agent: Drugs, biological products, or combination of drugs and biological products (approved or investigational) defined in the protocol as requiring expedited reporting to DAIDS. (DAIDS)
Study Physician: A physician listed on the Form FDA 1572 for IND studies or on the DAIDS Investigator of Record Agreement (IOR) for non-IND studies. (DAIDS)
Suspected Adverse Drug Reaction (SADR): An adverse event that could potentially have a causal relationship to a study agent (definitely, probably, possibly, probably not related or for deaths, pending). (DAIDS)

Toxicity: An adverse event that has an attribution of possibly, probably, or definitely related to a study agent. (DAIDS) NOTE: This term should not be used for expedited reporting of adverse events to DAIDS.

Unexpected Event: An adverse event, the nature or severity (intensity) of which is not consistent with the applicable product information (Investigator's Brochure, package insert, or summary of product characteristics for a US FDA-approved study agent. (DAIDS)

16.1 EAE Manual Appendix B: Contact Information for DAIDS Safety Office

All completed DAIDS Expedited Adverse Event Forms are submitted to the DAIDS Safety Office.
For questions or other communication, please note the following:

Website:	http://rcc.tech-res-intl.com
Office Phone*:	1-800-537-9979 (US only) or +1-301-897-1709
Office Fax*:	1-800-275-7619 (US only) or +1-301-897-1710
Office Email:	SAE@tech-res.com
Office Hours:	Monday through Friday, 8:30 AM to 5:00 PM<br>(US Eastern Time)
Mailing Address:	DAIDS Safety Office<br>6500 Rock Spring Drive<br>Suite 650<br>Bethesda, MD 20817

*Office phone and fax are accessible 24 hours per day.

17.1 EAE Manual Appendix C: Summary Chart for Expedited Reporting of Adverse Events to DAIDS for Protocol-Specified Study Agents

	Standard Level	Intensive Level	Targeted Level
**Deaths**	All Events	All Events	All Events
**Congenital anomalies, birth defects, fetal losses**	All Events	All Events	All Events
**Disabilities/Incapacities**	All Events	All Events	All Events
**Hospitalization**[1]	All Suspected Adverse Drug Reactions[2]	All Suspected Adverse Drug Reactions[2]	Unexpected Suspected Adverse Drug Reactions [2,3]
**Other events**	All Grade 4 Suspected Adverse Drug Reactions[2]	All Grades 3 and 4 Suspected Adverse Drug Reactions[2]	Unexpected Life-Threatening Clinical Suspected Adverse Drug Reactions [2,3]

[1]This category includes hospitalization, prolongation of hospitalization or requirement of intervention to prevent permanent disabilities or death.

[2]Suspected adverse drug reactions are adverse events that are assessed as definitely, probably, possibly, probably not related to a study agent (or for deaths, pending).

[3]Unexpected events are adverse events, of a nature or severity (intensity) that is not consistent with the applicable product information (package insert/summary of product characteristics) for a US FDA-approved study agent.

Timeframe for Expedited Reporting of Individual Adverse Events:

Adverse events requiring expedited reporting are to be reported to the DAIDS Safety Office **no later than 3 business days** after the site's recognition that the event fulfills the criteria for expedited reporting.

Protocol-Defined Expedited Adverse Event Reporting Period

The protocol-specified reporting level continues throughout the study/trial period (from enrollment of a subject through the end of study follow-up visits for that subject). The protocol may also require the same level of adverse event reporting to be continued beyond the end of study follow-up for each subject.
